# Gastric Cancer: Epidemiology, Risk Factors, Pathogenesis, Clinical Features, Diagnosis, and Management

**DOI:** 10.1002/mco2.71100

**Published:** 2026-10-29

**Authors:** Yixuan Xin, Xingya Wang, Yangtao Wu, Qian Liu, Qian Ren, Hui Wang, Jingbo Zhai

**Affiliations:** ^1^ School of Basic Medical Sciences Inner Mongolia Minzu University Tongliao China; ^2^ Mongolian Medical College Inner Mongolia Minzu University Tongliao China; ^3^ Key Laboratory of Zoonose Prevention and Control at Universities of Inner Mongolia Autonomous Region Tongliao China; ^4^ Brucellosis Prevention and Treatment Engineering Research Center of Inner Mongolia Autonomous Region Tongliao China

**Keywords:** diagnosis, epidemiology, gastric cancer, management, pathogenesis

## Abstract

Gastric cancer is a multifactorial disease shaped by diverse risk factors and a complex pathogenesis spanning from chronic atrophic gastritis to invasive malignancy. Its global incidence shows marked geographic disparities, and advances in understanding its pathogenic mechanisms have improved diagnosis and treatment. Although the relationship between gastric cancer and macrophages has been extensively investigated, a systematic review integrating the entire clinical and biological continuum of gastric cancer, from epidemiology to management, with a focus on macrophages remains lacking. This review summarizes the epidemiology and multidimensional risk factors for gastric cancer, including infectious, dietary, genetic, microbiome, and metabolite‐related factors. We describe the evolution of precancerous conditions, molecular alterations such as miRNA expression, and the bidirectional interactions between gastric cancer cells and macrophages that drive tumor progression within the tumor microenvironment. We also review clinical features, diagnostic approaches based on screening and endoscopy, and current management strategies, including surgery, systemic therapies, targeted therapies, immunotherapy, macrophage‐targeted therapies, macrophage metabolic reprogramming, and recent preclinical and clinical advances. Overall, this review integrates evidence from epidemiology, molecular biology, immunology, and pathology to provide a comprehensive framework for understanding gastric cancer pathogenesis and current as well as emerging therapeutic strategies.

## Graphical abstract

This comprehensive review summarizes gastric cancer epidemiology, multifactorial risk factors (like infectious, dietary, genetic), pathogenesis such as precancerous evolution, molecular alterations, and a focus on the bidirectional interactions between gastric cancer cells and macrophages in the tumor microenvironment. It then synthesizes features, clinical diagnosis based on screening and endoscopy, and current therapies including surgery, systemic therapies, targeted therapies, immunotherapy, macrophage‐targeted therapies, macrophage metabolic reprogramming, and recent preclinical and clinical advances. Overall, this review integrates evidence from epidemiology, molecular biology, immunology, and pathology to provide a comprehensive framework for understanding gastric cancer pathogenesis and current as well as emerging therapeutic strategies.

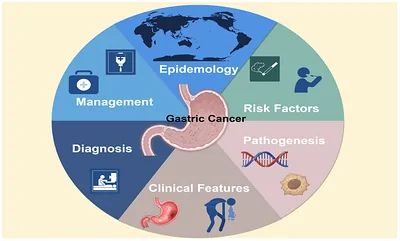

## 1 Introduction

Gastric cancer (GC) is the fifth most common cancer and the third leading cause of cancer‐related deaths worldwide. In 2020, an estimated 1.69 million new cases and 1.31 million GC‐related deaths were recorded globally [1]. According to the GLOBOCAN 2022 estimates, there are more than 1 million new cases of GC every year [2]. The GBD prediction model indicates that the global ASIR of GC is expected to decrease to approximately 10.89/100,000 by 2040 [3]. An IARC study based on GLOBOCAN 2022 data estimated that 15.6 million GC cases will occur among individuals born between 2008 and 2017, with 76% attributable to chronic *Helicobacter pylori* (*HP*) infection, making them potentially preventable through screen‐and‐treat programs [4]. In addition to *HP*, an umbrella review of 245 meta‐analyses confirmed that high‐salt intake, processed meat, and red meat increase GC risk, whereas cruciferous vegetables and other dietary protective factors reduce risk [5]. Over the past century, advances in epidemiology, molecular pathology, and endoscopic techniques have substantially improved our understanding of the disease. The development of therapeutic methods has also led to significant advances in the management of GC.

In recent years, the remarkable plasticity and diverse functions of macrophages based on their different subtypes have attracted increasing attention and become a major focus of solid tumor research. Throughout gastric carcinogenesis, macrophages undergo a dynamic polarization shift from an early M1‐dominant state that supports immune surveillance to a progressive M2‐dominant state that establishes an immunosuppressive microenvironment, which is tightly coupled with phenotypic repolarization and metabolic reprogramming [6]. For instance, CLEVER‐1^+^ tumor‐associated macrophages (TAMs) with an M2‐like phenotype accumulate in advanced GC, correlate with poor prognosis, and mediate chemoimmunotherapy resistance [7]. Similarly, GC cell‐derived exosomal PKM2 promotes the protumoral M2 phenotype polarization by enhancing lipid synthesis, which drives GC proliferation, migration, and invasion [8].

In this context, the present review aims to provide a systematic and integrated overview of GC, spanning epidemiology, risk factors, pathogenesis, clinical features, diagnosis, and management. We first systematically elaborate on the epidemiology and multidimensional risk factors, including infection, dietary, and genetic factors, as well as microbiome‐ and metabolite‐related influences. Mechanistically, we cover the evolution of precancerous conditions, molecular mechanisms such as miRNA expression, and the bidirectional interactions between GC cells and macrophages that perpetuate tumor progression within the tumor microenvironment (TME). We then focus on the clinical features of GC, including symptoms and various classification systems, its diagnosis through screening and endoscopy, and a summary of management strategies, including surgery, systemic therapies such as chemotherapy and radiotherapy (RT), targeted therapy, immunotherapy, macrophage‐targeted therapies, and recent preclinical and clinical advances. Specifically, ctDNA methylation biomarkers have been reported to achieve 100% specificity and 94% accuracy in predicting GC and distinguishing it from colorectal cancer and hepatocellular carcinoma [9]. In the NCT04130763 trial, nivolumab combined with fecal microbiota transplantation was well tolerated and achieved an objective response rate (ORR) of 25% and a disease control rate of 50% in patients with GC [10]. By integrating topics including epidemiology, immunology, physiology, molecular biology, and pharmacology, together with current intervention strategies and their clinical translation, this review provides a comprehensive and systematic perspective that may facilitate future research and therapeutic advances.

## 2 Epidemiology of GC

Having established the clinical significance and research backdrop of GC, we first quantify its global prevalence and demographic distribution to provide an epidemiological baseline for subsequent discussions.

### 2.1 Global Burden and Incidence

GC is a malignant tumor that seriously threatens human health, imposing a substantial disease burden worldwide [2]. According to the GLOBOCAN 2022 estimates, there are more than 1 million new cases of GC every year, with age‐standardized incidence rates (ASIRs) of 19.1 per 100,000 men and 7.2 per 100,000 women [2]. In the same year, there were 968,350 new cases of GC worldwide, ranking fifth among all malignant tumors [11]. According to data from 2020, the annual incidence of GC worldwide was approximately 1,089,000 (ASIR, 11.1/100,000), also ranking fifth among all cancers [12]. According to the Global Burden of Disease (GBD) 2021 data, the number of GC cases worldwide further increased to 1,230,233 (95% uncertainty interval: 1,052,350–1,409,970) in 2021, and the ASIR was 14.33/100,000 [3, 13, 14]. The absolute increase in the number of GC cases worldwide suggests that population growth and aging continue to drive up the total number of new cases despite a decline in age‐standardized rates [3, 13].

In terms of anatomical subtypes, GC can be divided into cardia and noncardia cancers. Approximately 85% of GC cases worldwide occur in the stomach body or antrum (noncardia), whereas only 15% occur in the cardia [11]. Noncardia GC predominates worldwide, especially in East Asia and other high‐incidence regions [15]. From a histological perspective, gastric adenocarcinoma accounts for more than 90% of cases, whereas other histological subtypes, such as neuroendocrine tumors, gastrointestinal stromal tumors, and lymphoid malignancies, are relatively rare [11]. Intestinal‐type GC is the most common subtype in high‐incidence regions worldwide and follows the Correa cascade, progressing from chronic gastritis to atrophic gastritis, intestinal metaplasia (IM), dysplasia, and finally invasive carcinoma [12, 16].

### 2.2 Mortality and Regional Variation

GC is one of the leading causes of cancer‐related death worldwide. According to the GLOBOCAN 2022 data, approximately 770,000 patients die from GC each year worldwide, ranking fourth among cancer‐related causes of death [2, 16]. The number of GC deaths in the same year was 659,853 [11]. In 2020, there were approximately 769,000 deaths from GC, with an age‐standardized mortality rate (ASMR) of 7.7/100,000. It ranked fourth among cancer‐related causes of death, after lung, colorectal, and liver cancers [12]. GBD 2021 data showed that the total number of GC deaths worldwide had reached 954,374 (95% uncertainty interval: 821,751–1,089,577) in 2021, and the ASMR was 11.20/100,000 [3, 13, 14]. The disability‐adjusted life years (DALYs) reached 22,786,633.10, and the age‐standardized DALY rate was 262.75/100,000 [14].

The incidence and mortality of GC show marked geographical variation worldwide, with incidence rates in high‐incidence regions being 5‐ to 10‐fold higher than those in low‐incidence regions [2, 16]. East Asia has the highest incidence of GC. In 2021, the ASIR reached 28.6/100,000, and the ASMR was 21.3/100,000 [3]. Among the regions classified by the United Nations, the ASIR in East Asia is much higher than that in other regions, followed by Eastern Europe, South America, and West Asia. Within East Asia, Mongolia has the highest incidence of GC in the world, followed by Japan and South Korea [15]. In 2021, the countries with the highest ASIRs were Mongolia (36.8/100,000), Afghanistan (32.9/100,000), and Bolivia (30.8/100,000). The ASIR was 29.1/100,000 in China, 25.8/100,000 in South Korea, and 25.54/100,000 in Japan [3, 13]. In terms of the absolute number of cases, there were approximately 612,000 new cases in mainland China in 2021 (accounting for 49.8% of the global total) and about 445,000 deaths (accounting for 46.6% of the global total), both of which were the highest worldwide [3].

There are also substantial differences in survival rates among regions. In the global cancer survival monitoring program CONCORD‐3, the 5‐year relative survival rate for GC from 2010 to 2014 was 20%–40% in most countries. However, South Korea (68.9%) and Japan (60.3%) had significantly higher survival rates than other countries, mainly because of organized endoscopic screening programs for GC and the resulting early diagnosis [15]. In the United States, the relative 5‐year survival rate (weighted average across all stages) for GC increased from 15% in 1977 to 36% in 2020 [11]. However, 35%–65% of patients in the United States still have metastatic disease at the time of initial diagnosis, and their 5‐year survival rate is less than 10% [11].

### 2.3 Demographic and Temporal Trends

In terms of sex distribution, both the incidence and mortality rates of GC show a significant male predominance. The incidence of GC in men is approximately two to three times higher than that in women worldwide [11, 12]. In 2020, the incidence of GC ranked fourth in men (15.8/100,000) and seventh in women (7.0/100,000) [12]. Data from 2021 showed that the ASIR in China was 44.48/100,000 in men and 15.23/100,000 in women. In Japan, the ASIR was 38.77/100,000 in men and 13.20/100,000 in women. In South Korea, the ASIR was 38.98/100,000 in men and 15.57/100,000 in women [13]. The male‐to‐female ratio of cardia cancer (4.2) was much higher than that of noncardia GC (1.6), suggesting that sex differences vary across anatomical sites [12].

In terms of age distribution, the incidence of GC increases significantly with age. The global incidence of GC peaked in the 65–69‐year age group in 2021, with an incidence rate of nearly 400/100,000 in men and approximately 150/100,000 in women [13]. In China, the peak incidence occurred in the 65–69‐year age group (approximately 450/100,000 in men) [13]. In Japan, the incidence among men aged 65–69 years reached 500/100,000 [13]. In South Korea, the incidence rate among men aged 60–64 years was 750/100,000 [13]. The median age at diagnosis of GC in the United States was 68 years [11]. It is worth noting that the disease burden of GC in women of childbearing age peaked in the 45–49‐year age group [17, 18].

In terms of temporal trends, the age‐standardized incidence of GC has generally decreased over the past four decades. From 1990 to 2021, the global ASIR of GC decreased from 24.76/100,000 to 14.33/100,000 (EAPC, −1.81), and the ASMR decreased from 22.01/100,000 to 11.20/100,000 (EAPC, −2.26) [13]. However, the rate of decline varied significantly across countries and regions: South Korea had the fastest decline in ASIR (EAPC, −3.54), followed by Japan (−2.98) and China (−1.64) [13]. This downward trend is mainly attributed to the declining prevalence of *HP* infection, improved hygiene conditions, the replacement of salt preservation with refrigeration, and increased consumption of fresh fruits and vegetables [2, 15]. However, one concerning trend is the rising incidence of early‐onset GC, particularly noncardia GC, in individuals younger than 50 years in the United States and other Western countries [11, 12, 16]. Between 2013 and 2022, the ASIR of GC in the United States increased from 1.2 to 1.8 per 100,000 among women younger than 50 years (4.0% annual change) and from 1.5 to 1.6 per 100,000 among men younger than 50 years (1.7% annual change) [11].

Regarding future trends, because of global population aging and the growth of high‐risk populations, the absolute number of new GC cases worldwide is projected to increase by 62% by 2040, reaching approximately 1.77 million cases [12]. Although the ASIR is expected to continue declining in most countries, the continued increase in the absolute number of cases poses a major challenge to GC prevention and control worldwide [3, 15, 18]. The GBD prediction model indicates that the global ASIR of GC is expected to decrease to approximately 10.89/100,000 by 2040 [3], and the ASIR among women of childbearing age (15–49 years) is projected to be 2.81/100,000 by 2050 [18]. These predictions suggest that while maintaining the downward trend in age‐standardized rates, strengthening primary prevention and early screening programs in high‐risk regions and populations remains a core strategy for reducing the overall burden of GC [2, 14].

## 3 Risk Factors for Gastric Cancer

The pronounced geographic disparities in epidemiological data suggest that GC is driven by specific risk factors.

### 3.1 Infectious Factors

The virulence factors of *HP* have a profound influence on GC. Vacuolating cytotoxin (VacA) can induce apoptosis of human gastric epithelial cells and inhibit the proliferation of T and B lymphocytes [19]. Cytotoxin‐associated gene A (CagA) protein can induce excessive reactive oxygen species (ROS) production in gastric epithelial cells, leading to DNA damage and thereby promoting the development of GC [20]. CagA translocates into polarized epithelial cells and disrupts the epithelial barrier [21]. CagA interacts with tumor necrosis factor (TNF) receptor‐associated factor 6 (TRAF6) and transforming growth factor‐β (TGF‐β)‐activated kinase 1 (TAK1) to activate the nuclear factor κB (NF‐κB) pathway and induce proinflammatory responses [22, 23]. CagA inhibits the expression of Let‐7b in gastric epithelial cells and promotes the growth and metastasis of GC cells [24]. The *HP*/NF‐κB/uridine phosphorylase 1 (UPP1) axis promotes the growth and invasion of precancerous lesions and GC cells [25]. *HP* increases programmed death ligand 1 (PD‐L1) expression in gastric epithelial cells, promotes T‐cell apoptosis, and contributes to the immune escape of GC cells [26]. *HP* infection regulates macrophage glucocorticoid receptor (NR3C1) signaling, ultimately regulating macrophage polarization and promoting the proliferation of GC cells [27] (Figure 1).

**FIGURE 1:**
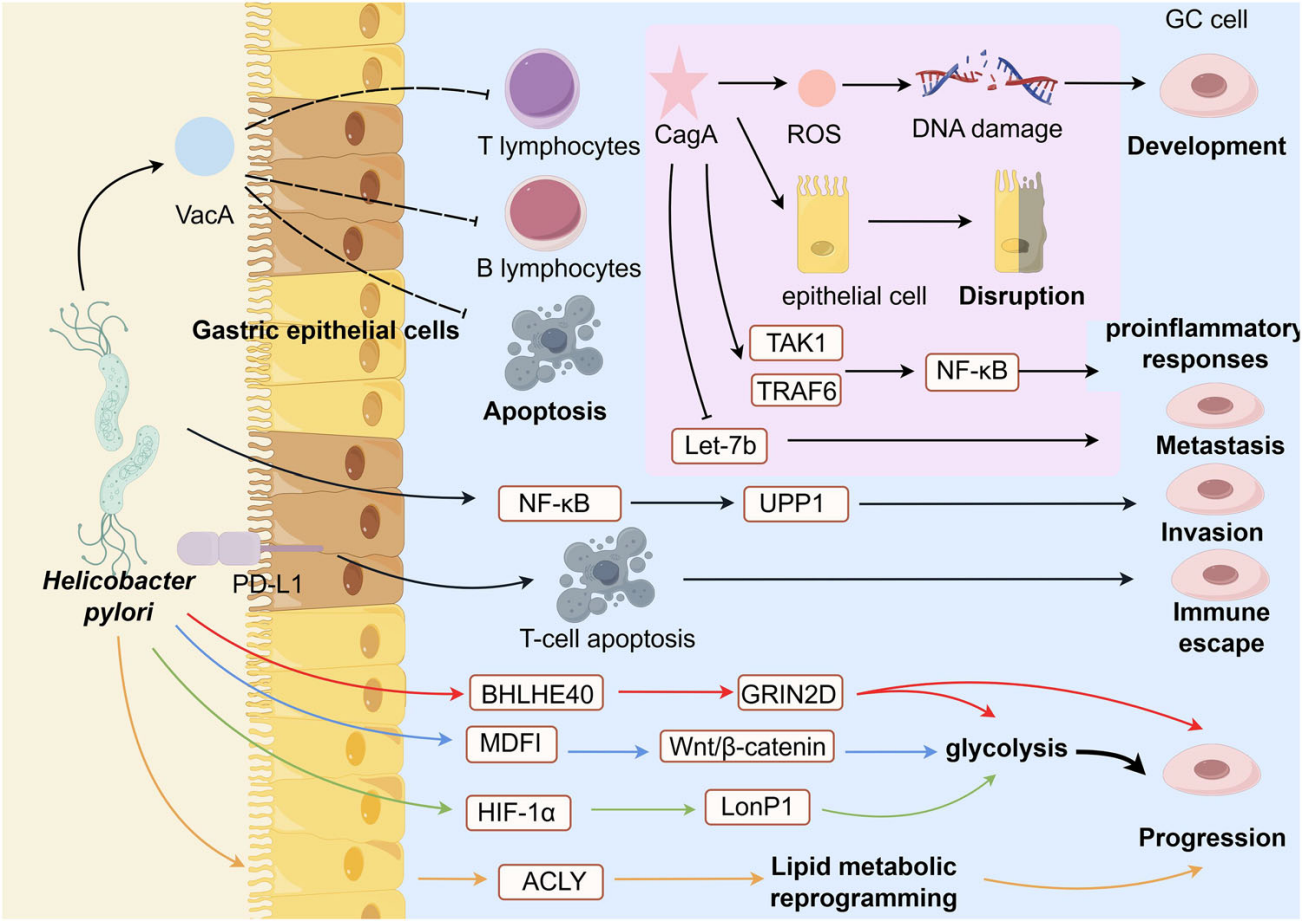
The Influence of *HP* in GC. *HP* can directly induce apoptosis of gastric epithelial cells and inhibit the proliferation of T and B lymphocytes through its virulence factor vacuolar cytotoxin (VacA). At the same time, the cytotoxin‐associated gene A (CagA) protein enters epithelial cells, induces excessive production of reactive oxygen species (ROS) to cause DNA damage and destroys the epithelial barrier. CagA can also interact with tumor necrosis factor receptor‐associated factor 6 (TRAF6) and transforming growth factor β activated kinase 1 (TAK1), which activates the NF‐κB pathway and triggers proinflammatory responses. CagA can downregulate the expression of Let‐7b in gastric epithelial cells and promote the growth and metastasis of GC cells. In addition, *HP* drives the invasive growth of precancerous lesions and GC cells through the NF‐κB/uridine phosphorylase 1 (UPP1) axis (pink arrow), and induces T‐cell apoptosis by upregulating programmed death ligand 1 (PD‐L1) to achieve immune escape (purple arrow). At the metabolic level, *HP*‐induced BHLHE40 upregulates GRIN2D expression and promotes glycolysis and GC progression (red arrow). *HP*‐induced MDFI enhances glycolysis through the Wnt/β‐catenin pathway (blue arrow). *HP* also accelerates glycolysis by upregulating LonP1 through HIF‐1α (green arrow). Glycolysis further promotes GC development. ACLY derived from gastric epithelial cells enhances the proliferation and malignant evolution of GC cells through lipid metabolic reprogramming (orange arrow). ACLY, ATP‐citrate lyase; HIF‐1α, hypoxia‐inducible factor 1‐alpha; LonP1, Lon protease 1; MDFI, MyoD family inhibitor. We used colored arrows to label the metabolic side of the responses to distinguish them from others.

*HP* also promotes GC through metabolic reprogramming. BHLHE40 in gastric epithelial cells, upregulated by *HP*, drives GRIN2D expression, contributing to glycolysis and the malignant progression of GC cells [28]. MyoD family inhibitor (MDFI), induced by *HP*, promotes glycolysis in GC cells through the Wnt/β‐catenin pathway [29]. *HP* accelerates glycolysis by upregulating Lon protease 1 (LonP1) through the action of hypoxia‐inducible factor 1α (HIF‐1α) [30]. UPP1, secreted by *HP*‐induced GC cells, promotes glycolysis through uridine metabolism [31]. Through glycolytic reprogramming, *HP* increases energy production and promotes GC progression.

*HP* also enhances lipid metabolic reprogramming. *HP* promotes lipid metabolism by stimulating ATP‐citrate lyase (ACLY) secretion in gastric epithelial cells [32]. By promoting the expression and demethylation of the glucocerebrosidase gene (*GBA1*), *HP* inhibits ferroptosis in GC cells [33]. Lipid metabolic reprogramming further enhances the proliferation and progression of GC cells.

Other bacteria also influence GC. *Streptococcus anginosus* induces gastritis, disrupts gastric barrier function, and promotes gastric tumor formation in mice [34]. Succinate produced by *S. anginosus* promotes GC progression through the succinate/SUCNR1/ABRAXAS1 axis [35]. *Lactobacillus paracasei* ZJUZ2‐3 releases indole‐3‐acetic acid (IAA), which inhibits the NF‐κB signaling pathway and ultimately suppresses GC development [36]. Lactic acid bacteria (LAB) are one of the major bacterial groups associated with GC. Lactic acid produced by LAB metabolism promotes GC cell growth and angiogenesis [37]. LAB can promote the production of ROS in vivo and induce DNA damage in colon cells [38]. In addition, LAB reduce nitrate to nitrite, induce angiogenesis, and inhibit apoptosis [38]. *Methylobacterium* inhibits CD8^+^ tissue‐resident memory T (TRM) cells and TGF‐β, thereby promoting GC development [39]. The binding of short‐chain fatty acids (SCFAs) to G‐protein‐coupled receptor 41 (GPR41) causes Th1 cells to secrete interleukin (IL)‐10 and aggravates inflammation [40].

### 3.2 Dietary and Lifestyle Factors

Diet and lifestyle are associated with GC risk. High intake of red meat, processed meat, and salt‐preserved foods may increase the risk of GC [41, 42]. Higher alcohol consumption, longer smoking duration, and a greater amount of tobacco consumed induce gastric inflammation and increase the risk of GC [43]. Individuals with depression have a higher risk of GC [5]. By contrast, high intake of allium vegetables is associated with a lower risk of GC [44]. Intake of vitamins A, C, and E is inversely associated with GC risk [5].

Diet is also associated with chronic atrophic gastritis (CAG). N‐nitroso compounds (NOCs) in food promote CAG development [45]. For example, long‐term exposure to low doses of 1‐methyl‐3‐nitro‐1‐nitrosoguanidine (MNNG) reduces the expression of inhibitor of growth protein 4 (ING4) in human gastric epithelial cells. It promotes phosphorylation of NF‐κB p65 and induces angiogenesis [46]. High‐salt diets also increase the risk of CAG. Mice fed a high‐salt diet show markedly increased expression of inflammatory cytokines and cytochrome P450 in gastric epithelial cells, accompanied by lipid peroxidation and malignant cell proliferation [47]. Alcohol consumption is also closely associated with CAG, affecting the degree of gastric atrophy and inducing inflammatory cell infiltration [48].

### 3.3 Chronic Gastric Inflammation and Precancerous Conditions

Refer to Section 4.1 for further details.

### 3.4 Genetic and Familial Risk

Familial syndromes are closely associated with GC. Juvenile polyposis syndrome, caused by mutations in the BMPR1A or SMAD4 tumor suppressor genes, is associated with an increased risk of gastric polyposis and GC [49]. Familial adenomatous polyposis, characterized by mutations in the APC tumor suppressor gene, also increases the risk of gastric polyposis and GC [49].

### 3.5 Microbiome and Metabolite Influences

Gut microbiota and their metabolites are a major focus of current research and play important roles in the occurrence and development of GC.

Gut microbiota and their metabolites can also inhibit the occurrence and development of GC. SCFAs produced by the gut microbiota inhibit inflammation by binding to GPR41 and inhibiting histone deacetylase (HDAC), thereby promoting IL‐22 secretion by CD4^+^ T cells and innate lymphoid cells [50]. SCFAs also increase mTOR activity in CD8^+^ T cells and promote antitumor immune responses [51]. *Bifidobacterium* can target the PD‐1/PD‐L1 axis and promote antitumor immunity [52].

Studies have shown that gut microbiota‐derived metabolites also regulate macrophage polarization. Most metabolites, such as butyrate and ursodeoxycholic acid, promote M2 macrophage polarization [53, 54]. However, only a few metabolites, such as cholesterol and indoxyl sulfate, promote M1 macrophage polarization [55, 56].

Epstein–Barr virus (EBV)‐encoded proteins promote the development of EBV‐associated GC (EBVaGC). Epstein–Barr nuclear antigen 1 (EBNA1) promotes the proliferation and metastasis of EBVaGC cells by inducing DNA methyltransferase 3A (DNMT3A) expression [57]. Latent membrane protein 1 (LMP1) in extracellular vesicles (EVs) promotes the expression of extracellular matrix (ECM)‐associated proteins, thereby enhancing GC cell invasion, migration, and ECM remodeling [58]. Latent membrane protein 2A (LMP2A) activates the expression of ETS homologous factor (EHF) and DNA methyltransferase 1 (DNMT1) through STAT3 phosphorylation. It also promotes the growth of EBVaGC cells [59], mediates the PI3K/Akt signaling pathway, increases survivin expression, and inhibits the apoptosis of EBVaGC cells [60].

EBV‐encoded miRNAs also play important roles in the development of EBVaGC. miR‐BART1‐3p promotes the growth and invasion of EBVaGC cells by targeting and inhibiting disabled homolog 2 (DAB2) [61]. EBV‐miR‐BART4‐5p inhibits the expression of BH3‐interacting domain death agonist (Bid) and suppresses apoptosis in EBVaGC cells [62]. miR‐BART5‐5p regulates PD‐L1 expression through the PIAS3/pSTAT3 pathway, thereby inhibiting apoptosis and promoting invasion of EBVaGC cells [63]. miR‐BART20‐5p targets and inhibits BAD expression, promoting the proliferation of EBVaGC cells [64]. miR‐BART11 and miR‐BART17‐3p target and inhibit FOXP1 and PBRM1 expression, respectively, thereby promoting PD‐L1 expression and immune escape in EBVaGC cells [65]. CircLMP2A under hypoxic conditions activates the KHSRP/VHL/HIF‐1α/vascular endothelial growth factor A (VEGFA) pathway and induces angiogenesis [66]. By contrast, miR‐BART6‐3p inhibits the proliferation of EBVaGC cells by regulating the LOC553103/STMN1 axis [67].

## 4 Pathogenesis of GC

The occurrence and development of GC is a gradual process involving multiple stages and factors.

### 4.1 Precancerous Lesions and Progression

#### 4.1.1 CAG

There are several major causes of CAG. *HP* not only injects CagA into gastric epithelial cells through the type IV secretion system but also activates β‐ADP‐heptose/ALPK1/TIFA/NF‐κB signaling, leading to DNA damage [68]. CagA interacts with E‐cadherin and promotes the translocation of β‐catenin into the nucleus [69]. The virulence factor VacA released by *HP* also induces apoptosis of gastric epithelial cells [70]. *HP* activates NOD‐like receptor thermal protein domain associated protein 3 (NLRP3) through the TNF/TNFR1 axis to mediate M1 macrophage polarization and promote the development of CAG [71]. *HP* also stimulates macrophages to express chemokine ligand 3 (CCL3) through the Janus kinase 1 (JAK1)/ signal transducer and activator of transcription 1 (STAT1) signaling pathway, thereby disrupting the gastric mucosal barrier [72].

Programmed cell death is closely associated with CAG. Autophagy protects gastric cells. Autophagy mediated by the mTOR signaling pathway protects the gastric mucosal epithelium from ethanol‐induced oxidative damage [73]. Aspirin inhibits autophagy in gastric epithelial cells and induces mucosal injury and apoptosis [74]. Abnormal autophagy leads to abnormal proliferation of gastric cells [75]. Autophagy‐related genes, such as *ATG7*, are associated with CAG progression [76]. Pyroptosis mediated by the NF‐κB/NLRP3/GSDMD pathway promotes the development of CAG [77]. NLRP3 mediates pyroptosis by activating caspase‐1 and cleaving GSDMD, thereby promoting the development of CAG [78]. The ferroptosis‐related gene *YWHAE* is involved in the development of CAG [79]. Hepcidin mediates ferroptosis in gastric cells through the IL‐6/STAT3 signaling pathway [79].

#### 4.1.2 Intestinal Metaplasia

*HP* infection causes parietal cell loss. The loss of parietal cells prevents the normal secretion of multiple signaling factors, reprograms the chief cell transcriptome, and ultimately leads to IM or spasmolytic polypeptide‐expressing metaplasia (SPEM) [80, 81, 82].

Bile acids (BAs) are closely associated with IM. High BA concentrations in the stomach are associated with an increased risk of IM and GC [83, 84]. The combined effects of BAs and IM biomarkers promote IM development [85]. Caudal‐type homeobox transcription factor 2 (CDX2) is expressed only in IM. Mucin 2 (MUC2) induces IM in CDX2 transgenic mice, whereas CDX2 knockout inactivates the expression of IM‐related immunohistochemical markers [86]. Sex‐determining region Y‐box 2 (SOX2) can be used as a molecular marker of IM [87]. During IM progression, SOX2 expression gradually decreases and is negatively correlated with the expression of CDX2 and other molecules in normal gastric and intestinal epithelial tissues [88]. BA‐induced miR‐21 directly binds to SOX2 and inhibits its expression, thereby promoting CDX2 transcription and IM development [89]. P1 promoter‐mediated hepatocyte nuclear factor‐4α (HNF4α) is expressed in IM tissues [90]. BAs activate the TGR5‐extracellular regulated protein kinase (ERK)1/2 pathway and induce HNF4α expression, thereby regulating Krüppel‐like factor 4 (KLF4) and CDX2 in gastric epithelial cells and promoting IM development [91]. BAs also reduce miR‐1 expression, inhibit the HDAC6/HNF4α/CDX2 pathway, and promote IM [92].

BA receptors are closely associated with IM development. Farnesoid X receptor (FXR) is a key regulator of BA homeostasis [93]. High BA concentrations induce HDC expression through FXR, and HDC may contribute to IM development [94]. Chenodeoxycholic acid activates FXR and increases CDX2 and MUC2 expression [95]. Deoxycholic acid activates the FXR/NF‐κB pathway, increasing CDX2 and MUC2 expression [96]. Activation of the TGR5‐ERK1/2 pathway also promotes HNF4α expression [91].

One study showed that deoxycholic acid stimulates the M2 muscarinic acetylcholine receptor (M2‐mAChR) in intestinal macrophages, driving TLR2 transcription by targeting AP‐1. Consequently, the NF‐κB/ERK/JNK signaling pathway is activated, inducing M1 macrophage polarization [97]. Gastric macrophage polarization may also be affected by BAs.

#### 4.1.3 SPEM

Macrophages play an important role in the development of SPEM. The number of M2 macrophages is significantly increased in the gastric mucosa with SPEM [98]. In late SPEM, IL‐33 expression in M2 macrophages is significantly increased, promoting the expression of Th2‐related cytokines such as IL‐13 and the development of SPEM through the IL‐33/IL‐33 receptor (ST2) signaling pathway [99]. Deoxycholic acid induces M2 macrophage‐derived exosomes and promotes SPEM development [100].

The IL family is closely associated with SPEM development. IL‐2 has been implicated in *HP*‐related gastric atrophy [101]. IL‐17A induces parietal cell apoptosis and promotes parietal cell atrophy and SPEM [101].

### 4.2 Molecular Pathways and Genetic Alterations

miRNAs are associated with the occurrence and development of GC. Most miRNAs promote GC cell invasion and progression. Decreased expression of miR‐204 in GC cells activates the SIRT1/LKB1 pathway and promotes GC cell invasion [102]. Overexpression of miR‐106a in GC cells is associated with TNM stage, whereas miR‐106b induces cell cycle arrest and apoptosis in TGF‐β‐dependent cells [103, 104]. miR‐296‐5p inhibits CDX1 expression through ERK1/2, thereby mediating the transformation of IM into GC [105]. miR‐21 targets RECK, PTEN, tropomyosin 1, and other tumor suppressors, thereby promoting GC development [106, 107]. miR‐222 is overexpressed in *HP*‐associated GC and promotes GC cell proliferation by targeting RECK [108]. Both miR‐130b and overexpressed miR‐301a inhibit the expression of the tumor suppressor gene *RUNX3*, thereby enhancing GC cell survival and promoting GC cell invasion and progression [109, 110]. Overexpressed miR‐181b in GC cells promotes GC cell invasion and proliferation by targeting and methylating TIMP3 [111]. miR‐223 is significantly overexpressed in patients with *HP*‐associated GC and promotes GC cell proliferation and metastasis [112]. Decreased miR‐218 expression in *HP*‐associated GC activates NF‐κB and promotes COX‐2 transcription, ultimately promoting GC cell proliferation [113]. Reduced miR‐141 expression in *HP*‐associated GC promotes GC cell proliferation [114]. Lipopolysaccharide (LPS) from *HP* inhibits miR‐375 expression, activates the JAK2/STAT3 signaling pathway, and promotes the occurrence and development of *HP*‐induced GC [115]. The miR‐106b‐25 cluster (miR‐25, miR‐93, and miR‐106b) is significantly upregulated in GC and inhibits apoptosis by suppressing p21 and Bim [116]. Decreased miR‐7 expression in *HP*‐associated GC increases IL‐1β and tumor necrosis factor α (TNF‐α) levels, thereby accelerating GC development [117]. Decreased miR‐328 expression in *HP*‐associated GC induces CD44 and CD44v overexpression, promoting the proliferation of SPEM cells [117]. miR‐27a targets prohibitin and promotes GC cell proliferation [118].

Some miRNAs inhibit GC development. miR‐146a expression is increased in *HP*‐associated GC, and miR‐146a inhibits cell apoptosis by regulating SMAD4 [119]. However, other studies have found that miR‐146a inhibits GC cell proliferation, promotes apoptosis, and suppresses the release of protumor cytokines and growth factors by inhibiting NF‐κB activation [120, 121]. miR‐146a also targets the L1 cell adhesion molecule and inhibits GC cell invasion and metastasis [122]. miR‐449 expression is decreased in *HP*‐associated GC and targets cell cycle control genes, inducing p53 pathway activation, inhibiting GC development, and promoting apoptosis [123]. miR‐203 expression is decreased in *HP*‐associated GC and inhibits GC cell growth and invasion through CASK. miR‐200b is overexpressed in patients with GC and inhibits GC cell proliferation and invasion by targeting ZEB2 [124].

miRNAs can enhance ADR resistance in GC cells. miR‐27a may induce ADR resistance by regulating P‐glycoprotein (P‐gp) in GC cells [125]. miR‐135a‐5p enhances ADR resistance through the AP‐2α/Bcl‐2 pathway [126]. miR‐19a and miR‐19b inhibit PTEN, thereby enhancing ADR resistance, and promote P‐gp expression, thereby inhibiting ADR‐induced apoptosis of GC cells. miR‐20a inhibits the expression of early growth response 2 (EGR2), thereby enhancing ADR resistance in GC cells [127]. miR‐21‐5p inhibits PTEN and TIMP3, thereby increasing ADR resistance in GC cells [128]. miR‐633 enhances ADR resistance by inhibiting Fas‐associated death domain (FADD) [129].

Some miRNAs act as tumor suppressors and inhibit ADR resistance in GC cells. Hypermethylation of the CpG island of miR‐129‐5p targets the ATP‐binding cassette (ABC) transporter family and inhibits ADR resistance in GC cells. Overexpression of miR‐508‐5p targets and inhibits the expression of ABCB1 and zinc ribbon domain‐containing 1 (ZNRD1), thereby reducing ADR resistance [130]. miR‐27b, miR‐107, and miR‐140 regulate CCNG1, SOX4, and Lin28, respectively, to reverse ADR resistance in GC cells and promote ADR‐induced apoptosis [131]. The miR‐200bc/429 cluster inhibits the expression of Bcl‐2 and X‐linked inhibitor of apoptosis (XIAP), thereby reducing ADR resistance in GC cells [132]. miR‐16, miR‐497, miR‐15b, and miR‐181b target Bcl‐2 expression in GC cells, inhibit ADR resistance, and promote ADR‐induced apoptosis [133]. Overexpression of miR‐103/107 inhibits P‐gp expression in GC cells by targeting Cav‐1, thereby increasing GC cell sensitivity to ADR [134].

Multiple miRNAs promote resistance of GC cells to platinum drugs. miR‐106a mediates the PTEN/AKT signaling pathway and targets RUNX3 to enhance resistance of GC cells to DDP [135, 136]. miR‐21 also enhances DDP resistance by inhibiting the PTEN/AKT pathway [137]. miR‐20a inhibits cylindromatosis (CYLD) expression, thereby inhibiting DDP‐induced apoptosis in GC cells [138]. miR‐223, miR‐141, and miR‐25 target F‐box and WD repeat domain‐containing 7 (FBXW7), kelch‐like ECH‐associated protein 1 (KEAP1), and forkhead box O3a (FOXO3a), respectively, thereby promoting DDP resistance [139]. Overexpression of miR‐491 and miR‐99a targets and inhibits calpain small subunit 1 (CAPNS1), thereby inhibiting DDP‐induced apoptosis in GC cells [140]. miR‐135b, miR‐4295, miR‐17‐5p, and miR‐193a‐3p inhibit mammalian STE20‐like kinase 1 (MST1), leucine‐rich repeats and immunoglobulin‐like domains 1 (LRIG1), p21, and SRSF2, respectively, thereby enhancing DDP resistance in GC cells [141]. miR‐421 enhances DDP resistance in GC cells by inhibiting the expression of E‐cadherin and caspase‐3 [142].

MiRNAs can also inhibit DDP resistance in GC. miR‐129, miR‐129‐5p, miR‐27b, and miR‐508‐5p enhance the sensitivity of GC cells to DDP by targeting ABC transporter family members, including P‐gp and ABCB1, respectively [143, 144, 145]. miR‐497, miR‐449a, miR‐181b, miR‐16, miR‐15b, and the miR‐200bc/429 cluster inhibit DDP resistance in GC cells by suppressing the Bcl‐2 pathway [132, 133, 146, 147, 148]. miR‐503, miR‐1217, and miR‐143 target and activate the IGF1R/IRS1 signaling pathway, thereby enhancing the sensitivity of GC cells to DDP [149, 150, 151]. miR‐30, miR‐148a‐3p, and miR‐181a inhibit LC3II, Ras‐related protein Rab‐12, A‐kinase anchoring protein 1 (AKAP1), and autophagy‐related 5 (ATG5), thereby promoting DDP‐induced apoptosis in GC cells [152, 153, 154]. Both miR‐192‐5p and miR‐138‐5p regulate the expression of excision repair cross‐complementation (ERCC), thereby inhibiting DDP resistance in GC cells [155, 156]. miR‐149 inhibits DDP resistance in GC cells by suppressing forkhead box M1 (FOXM1) expression [157]. Overexpression of miR‐34a inhibits mesenchymal–epithelial transition factor (MET) expression and improves the sensitivity of GC cells to DDP [158]. Overexpression of miR‐524‐5p targets SOX9 and promotes DDP‐induced apoptosis in GC cells [159].

MiRNAs are also associated with oxaliplatin resistance in GC. Inhibition of E2F transcription factor 1 (E2F1) and death‐associated protein kinase 2 (DAPK2) expression by miR‐135a enhances resistance of GC cells to oxaliplatin [160]. miR‐107 regulates Lin28 expression through the miR‐107/Lin28 pathway, improves the sensitivity of GC cells to oxaliplatin, and promotes oxaliplatin‐induced apoptosis of GC cells [131].

MiRNAs can enhance 5‐FU resistance in GC cells. miR‐17 inhibits the expression of death effector domain‐containing (DEDD) protein and enhances 5‐FU resistance in GC cells [161]. Overexpression of miR‐193‐3p reduces the sensitivity of GC cells to 5‐FU by targeting PTEN [162]. miR‐BART20‐5p targets and inhibits the expression of Bcl‐2‐associated death promoter (BAD), thereby inhibiting 5‐FU‐induced apoptosis of EBVaGC cells [64]. Conversely, miRNAs are also involved in suppressing 5‐FU resistance in GC cells. miR‐508‐5p, miR‐107, miR‐27b, and miR‐129‐5p inhibit the expression of ABC transporter family members and enhance the apoptotic effects of 5‐FU in GC cells [130, 131, 144, 145]. Both miR‐181b and miR‐429 inhibit Bcl‐2 expression, thereby improving the sensitivity of GC cells to 5‐FU [146, 163]. miR‐31 inhibits 5‐FU resistance in GC cells by targeting RhoA, E2F6, SMUG1, and enhancer of zeste homolog 2 (EZH2) [164, 165, 166].

*HP* also influences the effects of miRNAs. *HP* inhibits miR‐320 expression in a CagA‐dependent manner, thereby inducing Mcl‐1 expression, inhibiting apoptosis, and increasing the risk of GC [167]. miR‐101 targets SOCS2 in GC cells and inhibits GC cell growth, whereas *HP* CagA inhibits miR‐101 expression and promotes GC development [168]. miR‐370 inhibits GC cell proliferation by suppressing FOXM1 expression, whereas *HP* promotes GC cell proliferation by inhibiting miR‐370 through CagA, thereby promoting GC development [169]. miR‐490‐3p expression is decreased in *HP*‐induced GC [170]. CagA promotes IM by mediating the overexpression of miR‐584 and miR‐1290 through the NF‐κB and ERK1/2 pathways, respectively [171].

### 4.3 TME and Immune Context

GC cells exist within the TME, where macrophages influence GC through a variety of mediators. Macrophages and GC cells interact bidirectionally. On the one hand, GC cells influence macrophage phenotypes by secreting chemokines, enzymes, and other mediators. On the other hand, macrophages profoundly affect GC cell proliferation, invasion, and metastasis.

Activated by IFN‐γ from Th1 cells and LPS from bacteria, M1 macrophages exhibit strong antigen‐presenting and antitumor activities, secreting large amounts of proinflammatory cytokines (e.g., IL‐1β, IL‐6, and TNF‐α) that kill tumor cells [172]. and suppress tumor growth, thereby facilitating positive feedback [173]. In the NF‐κB pathway, p65–p50 dimers translocate from the cytoplasm to the nucleus, inducing M1 macrophages to express inducible nitric oxide synthase (iNOS), which metabolizes arginine into nitric oxide (NO) and citrulline. NO can further generate reactive nitrogen species (RNS), whereas citrulline supports NO synthesis through the citrulline–NO cycle [174]. High NO levels (> 500 nM) induce phosphorylation of the tumor suppressor protein p53 at Ser15 (p‐Ser15) [175], leading to tumor cell growth arrest or death. NO can also enter tumor cells and react with oxygen radicals to produce large amounts of peroxynitrite, resulting in protein nitrosylation and ultimately tumor cell apoptosis [175]. Additionally, M1 macrophages directly secrete NO through iNOS upregulation to kill tumor cells [175]. M1 macrophage‐derived ROS accumulation triggers the mitogen‐activated protein kinase (MAPK) and NF‐κB pathways, promoting tumor cell apoptosis [176], whereas ROS also activate the AKT and STAT3 pathways, inducing G2/M arrest and leading to tumor cell apoptosis [176]. M1 macrophages recruit Th1 cells to enhance antitumor immune responses [177]. Notably, M1 macrophages can directly phagocytose tumor cells and possess strong antigen‐presenting capabilities [178] (Figure 2).

**FIGURE 2:**
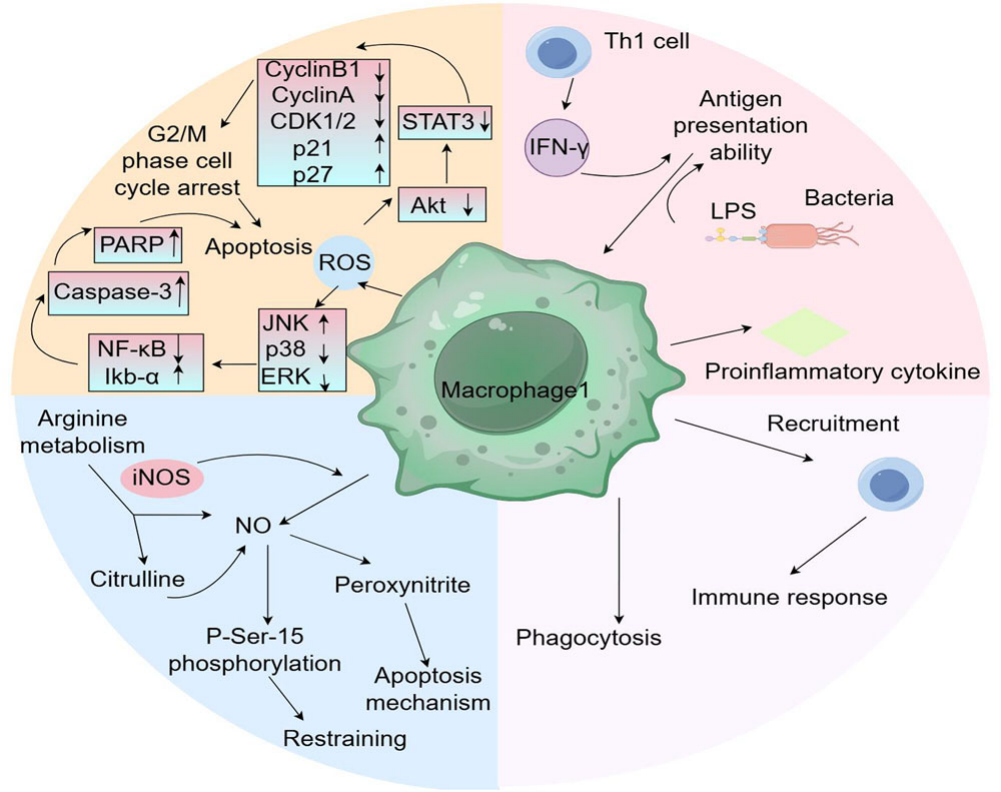
M1 macrophage function. IFN‐γ from Type 1 helper T cells (Th1) and LPS from bacteria enhance the antigen presentation and antitumor capabilities of M1 macrophages, enabling them to secrete large amounts of proinflammatory factors to kill tumor cells. M1 macrophage‐expressed iNOS metabolizes arginine into NO and citrulline, with citrulline supporting further NO synthesis via the citrulline–NO cycle. By contrast, NO can be converted into RNS, which enter tumor cells and induce apoptosis. High NO levels (> 400 nM) trigger P‐Ser15 phosphorylation, leading to tumor cell growth arrest or death. M1 macrophages also directly produce NO. Additionally, M1 macrophage‐generated ROS activates the MAPK and NF‐κB pathways to promote tumor cell apoptosis, while also triggering the AKT and signal transducer and activator of transcription 1 (STAT3) pathways, inducing G2/M arrest and leading to tumor cell apoptosis. M1 macrophage‐recruited Th1 cells further bolster the body's immune response. Finally, M1 macrophages can directly phagocytose tumor cells. CDK1/2, cyclin‐dependent kinase 1/2; ERKs, extracellular regulated protein kinases; IκB‐α, nuclear factor of kappa light polypeptide gene enhancer in B‐cell inhibitor α; JNK, c‐Jun N‐terminal kinase; PARP, poly ADP‐ribose polymerase.

Compared with M1 macrophages, M2 macrophages facilitate angiogenesis and tissue remodeling while suppressing helper T‐cell immune responses [179]. They also suppress tumor‐specific adaptive immunity, thereby promoting tumor growth and metastasis [180] (Figure 3).

**FIGURE 3:**
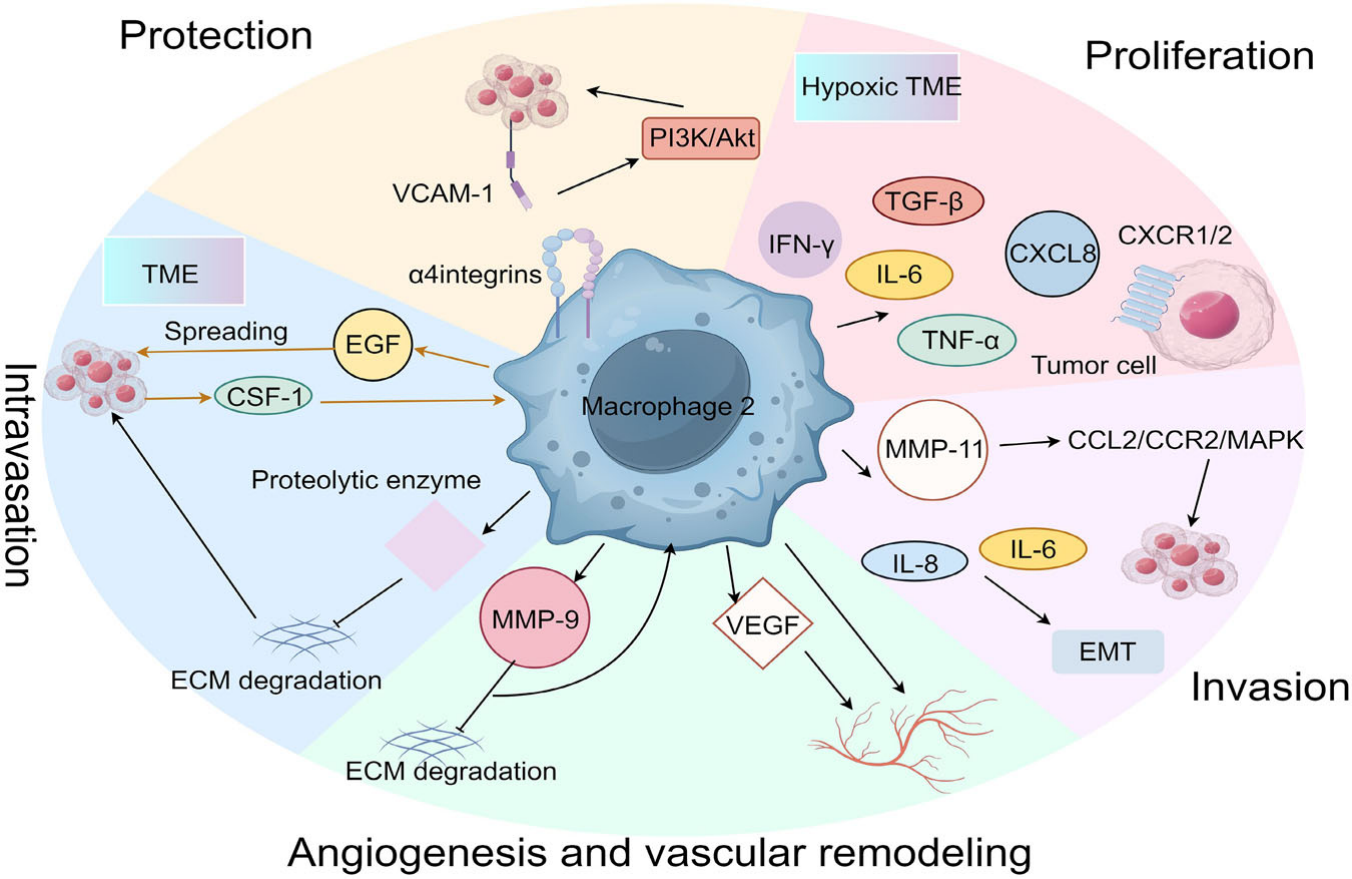
M2 macrophage function. M2 macrophage‐secreted signaling molecules (e.g., IL‐6) foster tumor cell growth. Under hypoxic conditions, M2 macrophage‐secreted CXCL8 binds to CXCR1/2 on tumor cells, stimulating their proliferation. M2 macrophage‐secreted matrix metalloproteinase‐11 (MMP‐11) enhances tumor cell invasion via the CCL2/CCR2/MAPK pathway. As tumor metastasis progresses, M2 macrophage‐secreted IL‐6 and IL‐8 induce epithelial–mesenchymal transition (EMT), facilitating tumor cell invasion. M2 macrophage‐secreted MMP‐9 degrades the extracellular matrix (ECM) and promotes the secretion of proangiogenic factors (e.g., vascular endothelial growth factor [VEGF]), with M2 macrophages also directly reshaping vasculature. Tumor cell‐derived colony‐stimulating factor 1 (CSF‐1) induces M2 macrophages to secrete epidermal growth factor (EGF), promoting tumor cell intravasation, while M2 macrophage‐secreted proteases degrade the ECM, aiding tumor cell intravasation. The α4 integrin on the M2 macrophage surface interacts with vascular cell adhesion molecule‐1 (VCAM‐1) on tumor cells, activating the PI3K/AKT pathway to protect tumor cells.

In the TME, signaling molecules secreted by M2 macrophages, including TNF‐α, IFN‐γ, IL‐6, and TGF‐β, can directly act on tumor cells and stimulate tumor growth [181]. Under hypoxic conditions, M2 macrophage‐derived CXCL8 activates the CXCR1/2‐JAK/STAT1 pathway, promoting GC cell proliferation [182].

The ECM facilitates tumor cell invasion through integrins, whereas integrin deficiency can lead to tumor cell dormancy [183]. M2 macrophage‐secreted chitinase 3‐like 1 (CHI3L1) binds to IL‐13Rα2 on GC cells, triggering marked upregulation of matrix metalloproteinases (MMPs), which activate the MAPK pathway and ultimately promote GC cell invasion [184].

ECM remodeling facilitates malignant tumor cell invasion. M2 macrophage‐secreted proteolytic enzymes, such as MMPs, play a key role in ECM remodeling [185]. For example, overexpression of MMP‐11 facilitates breast cancer cell metastasis through the CCL2/CCR2/MAPK pathway [186].

Epithelial–mesenchymal transition (EMT) is common in highly aggressive tumor cells. During tumor cell dissemination, M2 macrophages induce EMT by secreting cytokines and inflammatory mediators (e.g., IL‐6 and IL‐8), thereby facilitating tumor cell invasion [187]. Malignant tumors primarily metastasize through the tumor vasculature. As tumors grow, M2 macrophages actively regulate the “angiogenic switch” by secreting MMPs, which degrade the ECM and promote the release of proangiogenic factors such as VEGF, platelet‐derived growth factor, thymidine phosphorylase, and angiogenesis‐related chemokines [188]. Notably, MMP‐9 also modulates the bioavailability of VEGF [189]. Together, these effects contribute to the formation of high‐density tumor vasculature [190]. In addition, M2 macrophages remodel existing vasculature to facilitate tumor metastasis [191].

Tie2‐expressing monocytes, a subset of M2 TAMs, strongly promote angiogenesis. The tyrosine kinase receptor Tie2, expressed by these monocytes, binds to angiogenic factors and plays a crucial role in tumor vascularization [192].

Tumor cells enter the tumor vasculature through intravasation. In breast cancer, M2 TAMs, tumor cells, and endothelial cells collectively shape the TME, where tumor cell‐secreted colony‐stimulating factor 1 (CSF‐1) stimulates M2 macrophages to produce epidermal growth factor (EGF), facilitating tumor cell dissemination into the vasculature [193]. In addition, M2 macrophages release proteolytic enzymes that degrade the ECM surrounding endothelial cells [185]. These cascades ultimately enable tumor cell intravasation.

M2 macrophages support the survival of tumor cells in the vasculature and protect them from immune clearance [194]. The α4 integrin receptor on the macrophage surface interacts with VCAM‐1 expressed by intravasated tumor cells, activating the PI3K/AKT pathway and protecting tumor cells from proapoptotic cytokines [195].

CAG, a key precancerous stage in patients who eventually develop GC [196], is primarily induced by *HP*. At this stage, macrophages predominantly polarize toward the M1 phenotype, secreting abundant IL‐8 and some IL‐6, whereas only a small subset of M2 macrophages secretes little or no IL‐10 [197].

In the early stage of GC, activated M1 macrophages, together with other immune cells, generate ROS, RNS, and various growth factors, which activate oncogenes in adjacent gastric epithelial cells [198] (Figure 4).

**FIGURE 4:**
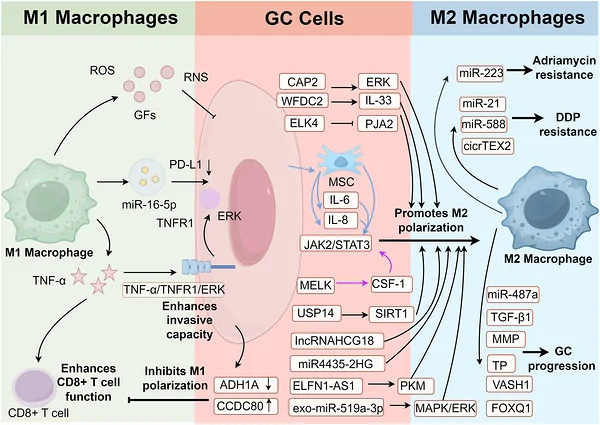
Interactions between macrophage phenotypes and GC. M1 macrophages promote GC development through ROS, RNS, and growth factors, while M1‐derived exosomes carrying miR‐16‐5p can inhibit the occurrence and progression of gastric cancer. M1‐overexpressed TNF‐α promotes GC development via the TNF‐α/TNFR1/ERK pathway while also enhancing the immune response of CD8^+^ T cells. M2‐derived exosomes contain miR‐223, miR‐21, circTEX2, and miR‐588. Among these, miR‐223 promotes adriamycin resistance in GC cells, while miR‐21, circTEX2, and miR‐588 contribute to cisplatin (DDP) resistance in GC cells, and miR‐487a promotes GC cell proliferation by targeting TIA1. GC cells express various proteins that regulate macrophage polarization. Overexpression of CAP2 induces M2 polarization through ERK signaling activation. WFDC2 stimulates IL‐33 release, promoting M2 polarization. GC‐derived mesenchymal stromal cells (MSCs) activate the JAK2/STAT3 pathway, enhancing IL‐6 and IL‐8 secretion and promoting M2 polarization (blue arrow). ELK4 inhibits PJA2 expression, further promoting M2 polarization. M2‐secreted enzymes, including TP, MMP, and VASH1, facilitate GC progression. Additionally, M2‐expressed cytokines such as FOXQ1, TGF‐β1, and various chemokines support GC progression. In GC cells, low expression of ADH1A and overexpression of CCDC80 inhibit M1 polarization. MELK, secreted by GC cells, promotes M2 polarization via the CSF‐1/JAK2/STAT3 pathway (purple arrow). USP14 stabilizes SIRT1, inducing M2 polarization. GC‐derived exosomes contribute to M2 polarization. ELFN1‐AS1 regulates pyruvate kinase M (PKM), enhancing M2 polarization. Enriched lncRNA HCG18 promotes M2 polarization. miR4435‐2HG facilitates M2 polarization. We used colored arrows to label responses related to the JAK2/STAT3 pathway to distinguish them from others.

GC arises within the chronic inflammatory environment of the gastric epithelium. Regulator of G‐protein signaling 1 has been identified as a potential target in the transition from chronic gastritis to GC [199]. It plays a role in M1 polarization and indirectly modulates M2 polarization through tumor cells.

Protein arginine methyltransferase 1 (PRMT1), expressed by GC cells, is associated with M1 macrophages. PRMT1 downregulation promotes M1 polarization by inhibiting the cGAS/STING axis [200].

M1 macrophages enhance CD8^+^ T‐cell function and eliminate GC cells through TNF‐α overexpression. However, TNF‐α also increases integrin αV expression in GC cells through the TNF‐α/TNFR1/ERK axis, which is regulated by VGLL1, thereby enhancing the invasive capacity of GC cells [201]. Additionally, M1 macrophage‐derived exosomes carrying miR‐16‐5p suppress tumor growth and may slow GC progression by triggering a T‐cell immune response [202].

GC progression leads to the accumulation of monocytes at the tumor site, where they polarize into M2 macrophages. Reduced alcohol dehydrogenase 1A (ADH1A) expression in GC cells inhibits M1 polarization [203], whereas CCDC80 overexpression in GC cells further suppresses M1 polarization and promotes M2 polarization [204]. Owing to hypoxia within the TME, M1 macrophage polarization and function are suppressed [205], and *HP* further impairs the M1 phenotype while promoting M2 polarization [206]. The conversion from M1 to M2 macrophages is a continuous process influenced by the accumulation of macrophage‐regulating factors, such as VEGF and endothelin‐2, in the hypoxic microenvironment, which drive M1 macrophages toward the M2 phenotype [207]. Binding of adenosine to Adora2a, an adenosine receptor in the TME, inhibits M1 polarization, whereas binding to A2B, another adenosine receptor, promotes M2 polarization [208]. Lactate induces the expression of VEGF and M2 polarization markers, such as Arg1 and Fizz1, while activating Gpr132, a G‐protein‐coupled receptor on macrophages, thereby further promoting M2 polarization [209, 210].

Proteins expressed by GC cells are involved in M2 TAM polarization. GC cells overexpress cyclase‐associated protein 2 (CAP2), which binds to receptor for activated C kinase 1 (RACK1) through its N‐terminus and promotes M2 macrophage polarization through activation of the ERK pathway. TGF‐β1 secreted by M2 macrophages induces the formation of premetastatic niches, facilitates GC cell migration and invasion, and stimulates CAP2 overexpression, thereby forming a positive feedback loop [211]. GC cell‐derived WFDC2 triggers IL‐33 release from epithelial cells, promoting M2 polarization [212]. GC‐derived mesenchymal stromal cells (MSCs) activate the JAK2/STAT3 pathway, enhancing IL‐6 and IL‐8 secretion and promoting M2 polarization [213]. Additionally, MSCs markedly enhance glycolysis in GC cells, leading to substantial lactate production and activation of the MCT/HIF‐1α axis in macrophages, thereby driving M2 polarization [214]. ELK4 derived from GC cells binds to the KDM5A promoter, suppresses PJA2 expression, modulates KSR1, and promotes M2 macrophage polarization [215].

Enzymes expressed by GC cells also mediate M2 TAM polarization. MELK overexpression in GC cells activates the CSF‐1/JAK2/STAT3 pathway, driving M2 polarization while also enhancing 5‐FU resistance [216]. Low AKR1B10 expression in GC cells induces M2 macrophage polarization [217]. Additionally, the deubiquitinating enzyme USP14 in the TME stabilizes SIRT1, which activates PGC‐1α‐mediated M2 polarization in macrophages [218].

Cytokines expressed by GC cells also influence M2 TAM polarization. IL‐6 and IL‐8 overexpressed by GC‐derived MSCs activate the JAK/STAT3 pathway, thereby promoting M2 polarization [213, 219]. In mouse models, TGF‐β1 oversecretion by mouse forestomach cells triggered M2 polarization [220]. In the gastric TME, elevated levels of chemokines such as CXCL5, CXCL12, and CCL5 further promote M2 macrophage polarization [221, 222, 223].

GC‐derived exosomes are closely linked to M2 polarization. GC‐derived exosome‐enriched ELFN1‐AS1 enhances the viability and stemness of GC cells, promoting M2 polarization through HIF‐1α‐dependent PKM regulation [224]. Enriched lncRNA HCG18 accelerates GC progression by inducing M2 polarization [225]. GC‐expressed miR4435‐2HG, upon exosomal delivery to macrophages, triggers M2 polarization [226], while miR‐541‐5p activates the DUSP3‐JAK2/STAT3 pathway, further promoting M2 polarization [227]. CircATP8A1 derived from GC cells induces M2 polarization and GC development via the miR‐1‐3p/STAT6 axis [228]. Additionally, serum exosome‐derived miR‐519a‐3p in patients with GC liver metastasis activates the MAPK/ERK pathway, leading to M2 macrophage polarization, which in turn accelerates GC liver metastasis through angiogenesis and premetastatic niche formation [229]. Let‐7g‐5p derived from GC cells induces M2 polarization and accelerates GC progression via *SERPINE1* [230]. Pyruvate kinase isoenzyme type M2 (PKM2) derived from GC cells enhances lipid synthesis in TAMs by inhibiting SCAP polyubiquitination and triggering SREBP1 nuclear accumulation, thereby promoting the protumoral M2 phenotype polarization and driving GC proliferation, migration, and invasion [8].

Cell surface receptors also contribute to M2 TAM polarization. Estrogen‐related receptor–activated GPR35 promotes macrophage infiltration and induces M2 polarization [231]. Macrophage scavenger receptor 1 enhances M2 polarization by accelerating arginine and proline metabolism in macrophages [231]. Additionally, PI3K/AKT pathway activation and noncoding RNAs such as LINC00665 drive M2 polarization [222, 232, 233].

Cancer‐associated fibroblasts (CAFs) are the predominant stromal cells in the GC TME. They express nicotinamide N‐methyltransferase (NNMT) and promote M2 polarization. Fat mass and obesity‐associated protein (FTO), derived from GC cells, promotes NNMT expression in CAFs through m6A modification, thereby enhancing M2 polarization [234].

M2 macrophage‐derived exosomes facilitate GC progression. M2‐derived exosomes transfer miR‐487a to GC cells, promoting proliferation by targeting TIA1 [235]. Exosome‐mediated transfer of functional apolipoproteins from M2 TAMs to tumor cells significantly enhances GC cell migration [236]. Additionally, M2‐derived exosomes deliver miR‐21, miR‐588, and circTEX2 to GC cells, increasing resistance to cisplatin [237, 238, 239]. Plasma exosomes enriched with miR‐223 enhance doxorubicin resistance in patients with GC [240].

M2 macrophage‐expressed enzymes contribute to GC development. M2 TAMs enhance GC vascularization through thymidine phosphorylase secretion and vasohibin‐1 upregulation [241]. M2 macrophage‐derived proteases, such as MMPs, promote GC cell proliferation, and migration. Additionally, M2 macrophages secrete legumain into both GC cells and the TME. In GC cells, legumain inhibits miR‐3978 transcription, facilitating tumor dissemination [242]. In M2 macrophages, inhibition of legumain suppresses GC cell growth and angiogenesis [242].

Cytokines expressed by M2 macrophages influence GC progression. M2 TAMs promote the transformation, invasion, and dissemination of EMT‐type GC cells by inducing transcription factors such as FOXQ1 and TGF‐β1 [243]. Additionally, CCL8 and other chemokines expressed by M2 macrophages contribute to GC cell proliferation, migration, drug resistance, and disease progression [244].

Sialic acid‐binding immunoglobulin‐like lectins (SIGLECs) expressed by M2 macrophages induce M2 polarization by activating the AKT/mTOR pathway [245].

Different M2 macrophage subtypes play distinct roles in GC. M2c macrophage polarization induces TGF‐β expression and inhibits ferroptosis in GC cells, thereby contributing to EMT and GC progression [246].

In the TME, the coexistence of M1 and M2 macrophages stimulates IL‐1β secretion, triggers the transformation of noncancerous fibroblasts, and upregulates IL‐6, IL‐24, and leukemia inhibitory factor, thereby supporting GC cell growth [247].

In advanced and metastatic GC, M2 macrophages suppress T‐cell immunity and secrete IL‐10 together with large amounts of anti‐inflammatory cytokines (e.g., TGF‐β), creating an immunosuppressive microenvironment that impairs antitumor immunity, facilitates immune evasion by GC cells, and directly enhances drug resistance [248, 249]. In patients with GC liver metastasis, glial cell line‐derived neurotrophic factor (GDNF) secreted by M2 macrophages enhances tumor cell resistance to apoptosis and promotes liver metastasis through the GDNF–GDNF family receptor α1 signaling axis [250]. A subset of CLEVER‐1^+^ TAMs with an M2‐like phenotype accumulates in advanced GC, associates with poor prognosis, and contributes to chemoimmunotherapy resistance through a PPARγ‐dependent lipid metabolism program [7].

Based on the role of macrophages in the occurrence and development of GC, macrophages have gradually become promising therapeutic targets for GC.

### 4.4 Metabolic Reprogramming and Metabolic Dependencies

In the metabolic reprogramming of GC, numerous metabolites and enzymes regulate cellular metabolism, thereby affecting the occurrence and development of GC.

#### 4.4.1 Carbohydrate Metabolism

Overexpression of the hexokinase 2 (HK2) isoform in GC enhances glycolysis and promotes GC cell proliferation and invasion [251]. In trastuzumab‐resistant human epidermal growth factor receptor 2 (HER2)‐positive GC, HK2 regulates glycolysis through protein kinase R‐like endoplasmic reticulum kinase (PERK)‐mediated circadian rhythm signaling [252]. The lactate dehydrogenase A (LDHA) isoform is overexpressed in GC cells through FOXM1, enhancing glycolysis and promoting GC cell proliferation and invasion [253]. Overexpression of α‐enolase (ENO1) promotes GC cell proliferation and metastasis [254]. Phosphoglycerate kinase 1 (PGK1) is overexpressed in GC cells, promoting GC cell invasion and potentially contributing to the peritoneal dissemination of GC [255]. PKM2 promotes GC cell growth by regulating p65 expression and thereby promoting Bcl‐xL transcription [256]. Glucose transporters (GLUTs) are overexpressed in GC, promoting glycolysis and GC cell invasion while also inducing M2 macrophage polarization [257]. AhpC/TSA antioxidant enzyme domain‐containing 1 (AAED1) promotes glycolysis and proliferation of GC cells by regulating the ERK/AKT1/HIF‐1α pathway and increasing HIF‐1α expression [258].

#### 4.4.2 Lipid Metabolism

The FA receptor CD36 is induced in GC cells, promoting the uptake of palmitic acid (PA) and mediating GC cell metastasis through the AKT/GSK‐3β/β‐catenin signaling pathway [259]. Loss of fatty acid‐binding protein 5 (FABP5) expression inhibits GC cell proliferation and invasion and significantly reduces cell viability [260]. Acetyl‐CoA carboxylase (ACC) activity is enhanced in GC, facilitating immune evasion by GC cells [261]. Diacylglycerol acyltransferase 1 (DGAT1) is highly expressed in GC cells and is associated with reduced overall survival (OS) in patients with poorly differentiated GC [262]. Expression of diacylglycerol acyltransferase 2 (DGAT2) is increased in metastatic GC, promoting lipid metabolism and peritoneal metastasis [263]. Loss of the adipose triglyceride lipase (*ATGL*) gene is associated with reduced survival in patients with GC [264]. 3‐Hydroxy‐3‐methylglutaryl coenzyme A reductase (HMGCR) is overexpressed in GC cells and promotes GC cell growth and metastasis through the Hedgehog/Gli1 signaling pathway [265]. Overexpression of sterol O‐acyltransferase 1 (SOAT1) promotes GC cell proliferation and invasion and also promotes lymphangiogenesis and lymph node (LN) metastasis by inducing VEGF‐C expression [266]. Natriuretic peptide receptor A (NPRA), overexpressed in GC cells, promotes GC cell metastasis and binds to PPARα to activate carnitine palmitoyltransferase 1B (CPT1B), thereby promoting FAO [267]. Stearoyl‐CoA desaturase (SCD) is significantly overexpressed during the IM and dysplasia stages of gastric carcinogenesis, promoting the occurrence and development of GC [268]. Fatty acid 2‐hydroxylase (FA2H) expression is decreased in GC, promoting GC cell proliferation, and drug resistance [269]. GC cells secrete large amounts of laminin subunit gamma 1 (LAMC1), which promotes preperitoneal niche formation and GC metastasis [270].

#### 4.4.3 Amino Acid Metabolism

S‐adenosylmethionine (SAM), produced during methionine metabolism, is required by tumor‐initiating cells for nucleic acid and histone methylation, thereby promoting the occurrence and development of GC [271]. SAM also promotes PD‐L1 expression and inhibits T‐cell activation, thereby facilitating immune evasion by GC cells [272].

Cysteine protease inhibitor 1 (CST1) is overexpressed in GC cells and interacts with GPX4 to enhance its stability. CST1 also mediates GPX4 ubiquitination through OTUB1, thereby inhibiting ferroptosis and promoting GC cell metastasis [273]. SLC7A11 promotes GPX4 expression and induces glutathione synthesis, thereby inhibiting ferroptosis and promoting peritoneal metastasis of GC cells [274]. Actin‐like protein 6A (ACTL6A), secreted by GC cells, induces glutathione synthesis, thereby inhibiting ferroptosis of GC cells [275].

Serine hydroxymethyltransferase 2 (SHMT2) is highly expressed in GC and mediates glycolysis, promoting GC cell proliferation, invasion, and metastasis through the HIF‐1α/VEGF/STAT3 signaling pathway [276]. During glutamine starvation, SEM (Stem‐like/EMT/Mesenchymal) type GC cells secrete large amounts of 3‐phosphoglycerate dehydrogenase (PHGDH), which mediates the mitochondrial folate cycle to remove ROS and maintain cell survival [277]. U2AF homology motif kinase 1 (UHMK1) is highly expressed in GC cells and promotes GC cell proliferation and invasion by regulating the de novo purine synthesis pathway [278].

## 5 Clinical Features of Gastric Cancer

The molecular and pathological alterations in the GC ultimately manifest as physical symptoms and signs.

### 5.1 Symptoms and Presentation

Early‐stage GC often presents with epigastric pain. As GC progresses, symptoms become more frequent and more severe. Common symptoms of advanced GC include epigastric pain, weight loss, indigestion, heartburn, and early satiety. Some patients also present with hematemesis, melena, and symptomatic anemia [279]. When the tumor obstructs the esophagus, dysphagia may occur. Jaundice is commonly seen in patients with biliary metastasis, liver metastasis, or peritoneal metastasis after recurrence following curative resection for advanced GC [280, 281]. If GC metastasizes to the ovaries (Krukenberg tumor), patients may develop abdominal bloating, ascites, and irregular vaginal bleeding [282].

### 5.2 Subtypes, Histology, and Molecular Classifications

The subtypes of GC vary according to the classification system used. The Laurén classification divides GC into intestinal, diffuse, and indeterminate subtypes. The intestinal subtype is characterized histologically by malignant epithelial cells with glandular differentiation infiltrating the tissue. The diffuse subtype is characterized by poorly differentiated cells and lack of cohesion [283]. According to the Goseki classification, Group I is characterized by well‐differentiated tubules and poor intracellular mucin, Group II by well‐differentiated tubules and abundant intracellular mucin, Group III by poorly differentiated tubules and poor intracellular mucin, and Group IV by poorly differentiated tubules and abundant intracellular mucin [284]. The World Health Organization classification divides GC into tubular, papillary, mucinous, poorly cohesive (including signet ring cell carcinoma), and mixed carcinomas based entirely on histomorphologic appearance [283].

Based on molecular classification, GC comprises several subtypes. The Cancer Genome Atlas (TCGA) Research Network classified GC into four subtypes: EBV‐positive tumors, characterized by CDKN2A (p16^INK4A^) promoter hypermethylation and PIK3CA, ARID1A, and BCOR mutations; microsatellite instability (MSI) tumors, characterized by MLH1 promoter hypermethylation; genomically stable (GS) tumors, characterized by RHOA mutations; and chromosomal instability (CIN) tumors, characterized by TP53 mutations [285] (Figure 5).

**FIGURE 5:**
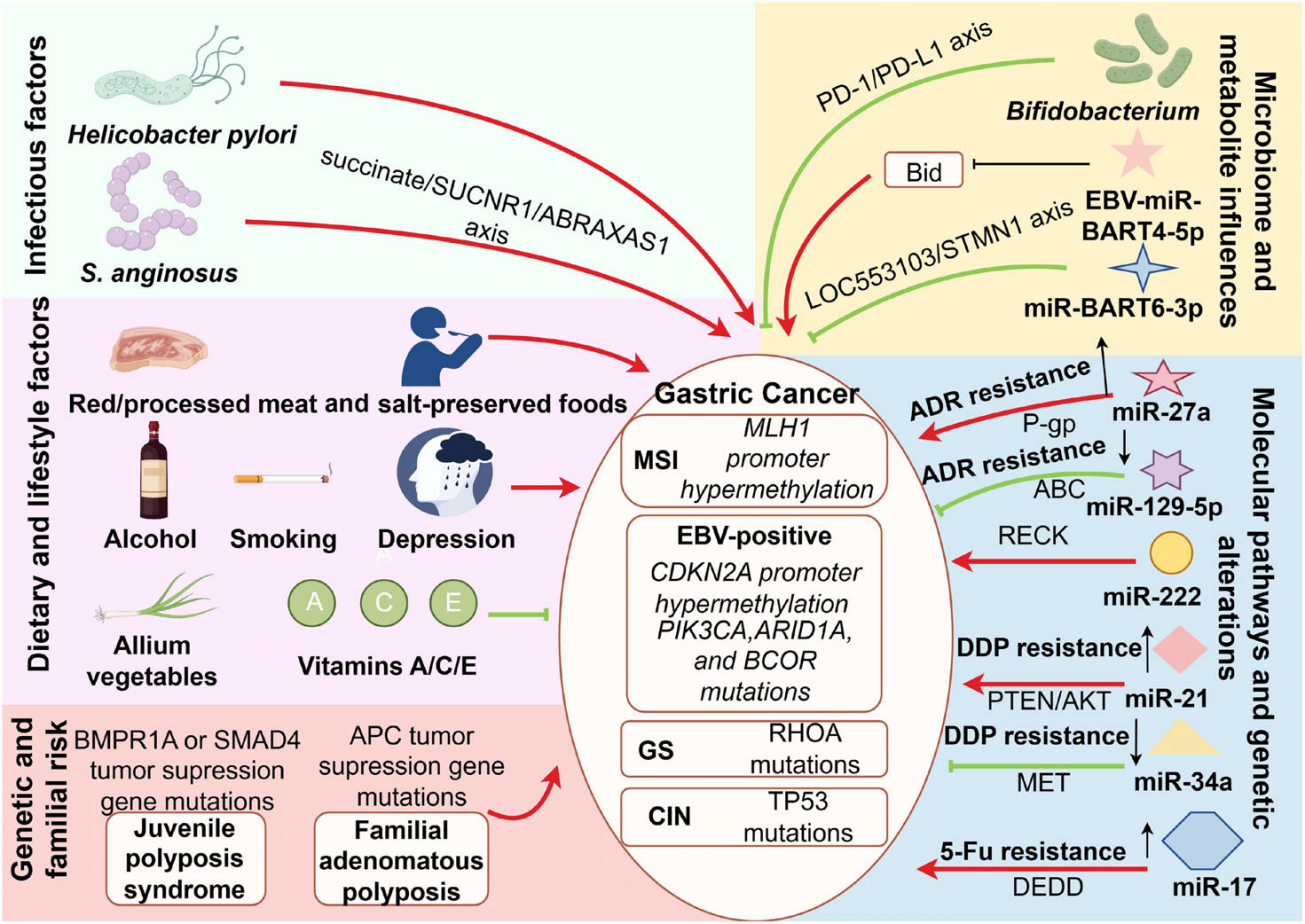
Risk factors and molecular pathology landscape of gastric cancer. GC comprises four TCGA molecular subtypes: EBV‐positive tumors, characterized by CDKN2A (p16^INK4A^) promoter hypermethylation and PIK3CA, ARID1A, and BCOR mutations; microsatellite instability (MSI) tumors, characterized by MLH1 promoter hypermethylation; genomically stable (GS) tumors, characterized by RHOA mutations; and chromosomal instability (CIN) tumors, characterized by TP53 mutations. The function of *HP is shown in* Figure 1. *S. anginosus* promotes GC progression through the succinate/SUCNR1/ABRAXAS1 axis. Dietary and lifestyle also have an influence on GC. High intake of red meat, processed meat, salt‐preserved foods and individuals with depression may increase the risk of GC. Alcohol consumption and smoking also increase the risk of GC. By contrast, high intake of allium vegetables and vitamins A, C, and E are associated with a lower risk of GC. Familial syndromes are closely associated with GC. Juvenile polyposis syndrome caused by mutations in the BMPR1A or SMAD4 tumor suppressor genes, and familial adenomatous polyposis characterized by mutations in the APC tumor suppressor gene increase the risk of gastric polyposis and GC. In the microbiome and metabolite, *Bifidobacterium* can target the PD‐1/PD‐L1 axis and promote GC development. EBV‐miR‐BART4‐5p inhibits the expression of Bid and suppresses apoptosis in GC cells. By contrast, miR‐BART6‐3p inhibits the proliferation of GC cells by regulating the LOC553103/STMN1 axis. Other miRNAs also have various functions in GC. miR‐27a may induce ADR resistance by regulating P‐gp in GC cells. miR‐129‐5p targets the ABC transporter family and inhibits ADR resistance in GC cells. miR‐222 promotes GC cell proliferation by targeting RECK. miR‐21 enhances DDP resistance by inhibiting the PTEN/AKT pathway. Overexpression of miR‐34a inhibits MET expression and improves the sensitivity of GC cells to DDP. miR‐17 inhibits the expression of DEDD protein and enhances 5‐FU resistance in GC cells. Due to space constraints, only representative examples are shown; the list is not exhaustive. For comprehensive details, please refer to Sections 3 and 4.2 of the main text.

The Asian Cancer Research Group classified GC into four subtypes: MSI tumors, characterized by loss of *MLH1*; microsatellite stable with EMT (MSS/EMT), characterized by loss of *CDH1*; microsatellite stable with TP53 activity (MSS/TP53+); and microsatellite stable without TP53 activity (MSS/TP53−) [286].

### 5.3 Prognostic Factors and Outcome Benchmarks

The prognostic factors and outcome benchmarks of GC are associated with genes, proteins, and GC subtypes. Single‐nucleotide polymorphisms are prognostic biomarkers. ERCC1 rs11615 polymorphisms are associated with outcomes in patients with GC receiving oxaliplatin‐based chemotherapy [287]. The lncRNA H19 rs2839698 polymorphism is associated with OS in patients with T3 gastric adenocarcinoma who underwent postoperative treatment [288]. Expression levels of miR‐635 and miR‐519d were higher in the survival group and effectively predicted prognosis [289]. A higher preoperative albumin‐to‐carcinoembryonic antigen ratio was associated with better OS and disease‐free survival (DFS) in patients with Stage III–IV GC [290]. Poor outcomes were associated with higher expression of transmembrane protein 119 in patients with GC [291]. Six prognostic genes (*ELANE*, *ASCL2*, *APOA1*, *GRP*, *CD36*, and *MUC15*) related to N7‐methylguanosine‐related genes (m7G‐RGs) were associated with prognosis. Lower expression of *ELANE*, *APOA1*, *GRP*, *CD36*, and *MUC15* was associated with a more immunosuppressive microenvironment and poorer prognosis, whereas *ASCL2* showed the opposite pattern [292].

According to the Laurén classification, patients classified as having the intestinal type had a higher 5‐year survival rate than those classified as having the diffuse type [293]. Patients with distal GC had a better 5‐year survival rate than those with proximal GC [294].

## 6 Diagnosis of GC

In clinical practice, the diagnosis of GC is typically established through screening strategies, risk stratification, endoscopy, biopsy, and the TNM staging framework.

### 6.1 Screening and Risk Stratification Approaches

There are two types of serum pepsinogens: pepsinogen I (PGI) and pepsinogen II (PGII), which reflect the function and morphology of the gastric mucosa. With the development of atrophic gastritis, the serum PGI level gradually decreases, whereas the PGII level remains relatively stable [295]. Some studies have shown that the PGI/PGII ratio is inversely associated with the risk of GC [296]. However, PG levels are influenced by multiple factors, including age, sex, and lifestyle [295].

A prediction risk score based on age, sex, body mass index, family history of GC in first‐degree relatives, and consumption of pickled foods showed satisfactory discriminatory performance. Among high‐risk participants, 85.2% of GC cases were detected by gastroscopy [297]. A prognostic risk score model including the *MPO*, *BST1*, *IL6*, *KCNJ15*, *SELP*, and *ELANE* genes showed that patients in the low‐risk gene (LNRG) group had longer survival than those in the high‐risk gene (HNRG) group. A study published in 2025 reported that methylated *RIMS1* levels could serve as a risk stratification marker [298]. These risk stratification approaches facilitate GC screening and prognostic prediction.

### 6.2 Endoscopy, Biopsy, and Histopathology

Endoscopy plays an important role in the diagnosis of GC. Gastric atrophy is characterized endoscopically by pale mucosa, increased visibility of blood vessels, loss of gastric folds, and an atrophic border [299]. IM is characterized by mottled patchy erythema (defined as multiple flat or slightly depressed erythematous lesions) on conventional endoscopy and by a light blue crest and a marginal turbid band on magnifying narrow‐band imaging [300, 301]. Gastric neoplasia is characterized by irregular glands and microvessels, accompanied by loss of the normal pit and mucosal patterns. Persistent or nonhealing gastric ulcers are also characteristic findings [302].

Biopsy combined with metabolomic analysis has shown promise in GC diagnosis. A study published in 2026 reported that upregulated metabolites were closely associated with amino acid metabolism, methylation, and redox regulation, whereas downregulated metabolites were involved in nucleotide metabolism and energy‐related pathways [303]. In the arginine biosynthesis pathway, metabolites such as arginine, ornithine, and L‐argininosuccinic acid, as well as enzymes including ornithine transcarbamylase (OTC), ornithine decarboxylase 1 (ODC1), and argininosuccinate synthase 1 (ASS1), were upregulated in GC. Receiver operating characteristic curve analysis identified ornithine and citrulline as strong discriminators [303]. In pyrimidine metabolism, uridine and uracil levels were increased, whereas cytidine monophosphate (CMP) and cytidine levels were decreased. Adenosine monophosphate (AMP), guanosine triphosphate (GTP), uridine monophosphate (UMP), and other metabolites may also discriminate GC from normal tissue [303].

### 6.3 Imaging for Staging

TNM staging includes primary tumor (T), regional LNs (N), and distant metastasis (M). The current TNM classification is based on the latest *AJCC Cancer Staging Manual* (eighth edition). For example, T1a describes tumor invades lamina propria or muscularis mucosae. N0 describes no regional LN metastasis. M0 describes no distant metastasis. IA (T1N0M0) describes the staging of GC [304].

### 6.4 Biomarkers and Liquid Biopsy

PD‐L1 is considered an important prognostic biomarker in GC. PD‐L1 expression is associated with GC progression, tumor invasiveness, and patient survival. PD‐L1 can bind to and activate epidermal growth factor receptor (EGFR), thereby inhibiting apoptosis of GC cells. HER2 binds to and activates its ligand, and HER2 overexpression promotes GC cell proliferation by regulating the expression of cell cycle‐related proteins. HER2 also regulates the Wnt/β‐catenin pathway to promote GC cell invasion and metastasis. Serum VEGF levels are associated with GC classification. VEGF secreted by GC cells promotes lymphangiogenesis and lymphatic metastasis. Studies have shown that patients with high tumor mutational burden have higher ORRs and better therapeutic responses. Albumin expression derived from tumor cells is increased in advanced T and N stages (T2–T4 and N1–N3), and albumin knockdown suppresses GC cell proliferation and migration [305].

Liquid biopsy analyzes circulating tumor cells (CTCs), circulating tumor DNA (ctDNA), and other components in body fluids for early diagnosis, treatment monitoring, and prognostic assessment. CTCs released from the primary tumor are cells that invade blood vessels through EMT and are strongly associated with GC prognosis [306]. A meta‐analysis showed that patients with CTC‐negative status had longer OS and progression‐free survival (PFS) than those with CTC‐positive status [307]. ctDNA consists of small DNA fragments released by tumor cells. ctDNA methylation plays an important role in detecting early‐stage GC in blood. One study reported that ctDNA methylation biomarkers achieved 100% specificity and 94% accuracy for predicting GC and distinguishing GC from colorectal cancer and hepatocellular carcinoma [9].

## 7 Management of GC

Accurate diagnosis and precise clinical staging constitute the cornerstone of rational therapeutic decision‐making. Building upon the diagnosis, this section comprehensively reviews current multimodal treatment strategies, encompassing surgery, chemoradiotherapy, targeted therapy, immunotherapy, and macrophage‐targeted therapeutic strategies (Figure 6).

**FIGURE 6:**
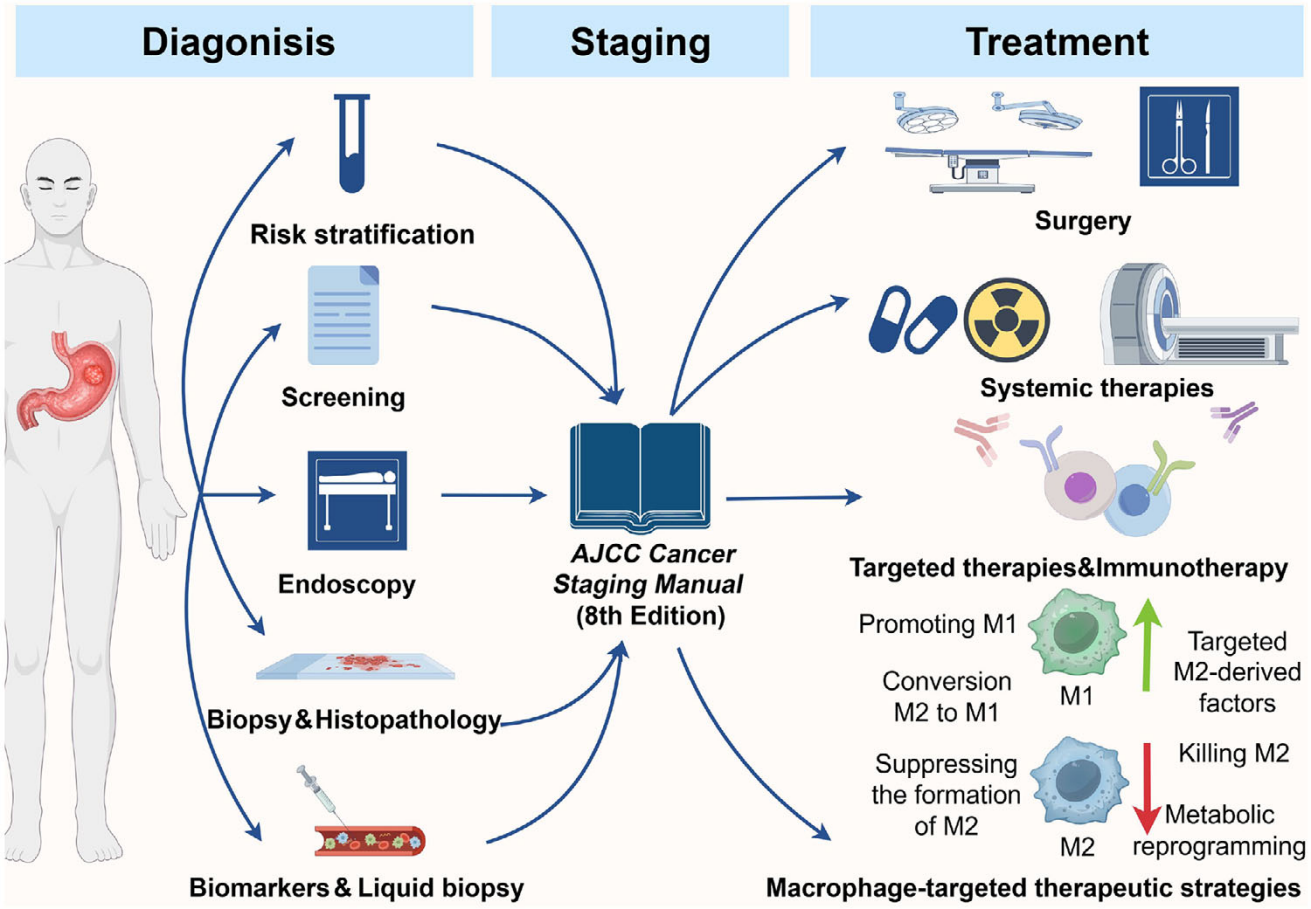
Diagnostic and treatment management algorithm for gastric cancer. The process of diagnosis and treatment begins with diagnostic evaluation, including screening, risk stratification, endoscopy, biopsy, histopathology, biomarkers and liquid biopsy, and the *AJCC Cancer Staging Manual* (eighth edition). Based on diagnosis and clinical staging, there are various treatment management options: surgery, systemic therapies, targeted therapies, immunotherapy, and macrophage‐targeted therapeutic strategies. Due to space constraints, only representative examples are shown; the list is not exhaustive. For comprehensive details, please refer to Sections 6 and 7 of the main text.

### 7.1 Surgical Approaches

Endoscopic resection includes endoscopic mucosal resection (EMR) and endoscopic submucosal dissection (ESD). According to the 2025 MAPS III guidelines, EMR is an alternative for Paris 0‐IIa lesions of ≤ 10 mm with a low likelihood of malignancy. ESD is recommended for differentiated gastric lesions clinically staged as low‐grade dysplasia, high‐grade dysplasia, or intramucosal carcinoma of ≤ 30 mm if ulcerated or regardless of size if nonulcerated. ESD is also recommended for malignant lesions that are clinically staged as intramucosal, undifferentiated, and ≤ 20 mm if nonulcerated or as having minimal submucosal invasion if differentiated and ≤ 30 mm [308]. Compared with ESD, EMR is less time‐consuming and has a lower risk of perforation, whereas ESD has a higher en bloc resection rate, a higher histologic curative resection rate, and a lower postoperative local recurrence rate [309].

Lymphadenectomy includes D1 and D2 lymphadenectomy. D1 lymphadenectomy involves the perigastric LN stations (N1), including the right cardial, left cardial, lesser curvature, greater curvature, suprapyloric, and infrapyloric nodes. D2 lymphadenectomy includes the N1 stations together with the LNs around the branches of the celiac axis (N2), including the left gastric artery, common hepatic artery, celiac trunk, splenic hilum, proximal splenic artery, and hepatoduodenal ligament LNs along the proper hepatic artery [310]. A trial conducted in the United Kingdom found that D2 lymphadenectomy was associated with higher postoperative morbidity and mortality [311]. A 15‐year follow‐up study in the Netherlands showed that D1 lymphadenectomy was associated with higher rates of local recurrence, regional recurrence, and GC‐related death [312].

A study published in 2026 showed that open total gastrectomy was associated with greater blood loss, whereas laparoscopic total gastrectomy required a longer median operative time in patients with advanced GC [313]. An analysis published in 2026 found no statistically significant differences between the two procedures in survival or the incidence of postoperative complications among patients with Stage 0‐I GC [314].

### 7.2 Systemic Therapies (Chemotherapy, RT)

Perioperative chemotherapy has been widely used in locally advanced GC. The ECF regimen, consisting of epirubicin, cisplatin, and 5‐FU, can reduce tumor stage and size and improve OS [315]. The FLOT regimen consists of oxaliplatin, 5‐FU, leucovorin, and docetaxel. Patients who received FLOT had a longer median OS than those who received a 5‐FU/platinum regimen [316]. The XELOX regimen, which includes capecitabine and oxaliplatin (CAPOX), achieved a better 3‐year DFS after D2 gastrectomy than D2 gastrectomy alone; however, it was associated with a higher incidence of Grade 3 or 4 adverse events and a lower 3‐year DFS than the DOX regimen in a Phase III trial [317, 318]. A retrospective cohort study showed that patients who received postoperative adjuvant chemotherapy had longer survival than those who underwent surgery alone [319]. In a study of Stage II–III GC, patients with Stage IIIC disease who received paclitaxel/S‐1/oxaliplatin (PSOX) had a higher DFS rate than those who received S‐1/oxaliplatin (SOX) [320]. The DOS regimen, consisting of docetaxel, oxaliplatin, and S‐1, significantly increased the R0 resection rate and demonstrated antitumor activity in a Phase II clinical trial [321].

Hemostatic RT is a well‐tolerated treatment for tumor‐related bleeding. One study showed that patients treated with 20 Gy in five fractions achieved an 80% response rate [322]. In a Phase II trial, treatment with 36 Gy delivered in 12 daily fractions effectively relieved pain and obstruction, and bleeding was controlled in 80% of patients [323]. A case report demonstrated disappearance of tumor vessels and tumor staining on angiography 2 h after hemostatic RT, with no active bleeding observed on upper gastrointestinal endoscopy performed 10 days later [324].

### 7.3 Targeted Therapies

Overexpression of HER2 promotes GC cell invasion, proliferation, and metastasis by activating the PI3K/AKT/mTOR pathway. HER2‐amplified tumor cells also exhibit enhanced immune evasion [325]. Trastuzumab targets extracellular domain IV of HER2 and inhibits downstream signaling. One study showed that OS was significantly prolonged in HER2‐positive patients treated with trastuzumab plus chemotherapy [326].

VEGF drives pathologic angiogenesis and lymphangiogenesis and promotes metastasis through the PI3K/AKT/mTOR, PLCγ/PKC/Raf/MEK/ERK, and FAK/Src/Rac pathways [327, 328]. Ramucirumab inhibits angiogenesis by targeting VEGFR2 [329]. In 2023, a Phase II/III trial showed that perioperative FLOT (5‐FU, leucovorin, oxaliplatin, and docetaxel) plus ramucirumab numerically improved median DFS in patients with resectable esophagogastric adenocarcinoma [330]. Apatinib also inhibits angiogenesis by targeting VEGFR2 [331]. A Phase II study published in 2024 showed that camrelizumab plus apatinib and chemotherapy achieved a confirmed ORR of 76.5% and a median PFS of 8.4 months in patients with advanced GC [332].

### 7.4 Immunotherapy

PD‐1 binding to PD‐L1 inhibits effector T‐cell function and promotes tumor progression and immune resistance [333]. Nivolumab restores the antitumor function of T cells by blocking PD‐1/PD‐L1 interaction. Study NCT04130763 found that nivolumab combined with fecal microbiota transplantation was well tolerated and achieved an ORR of 25% and a disease control rate of 50% in patients with GC [10]. Avelumab activates the antitumor immune response by blocking PD‐L1. A Phase II study found that avelumab combined with trastuzumab and mFOLFOX6 achieved a median PFS of 8.0 months and a median OS of 13.1 months in previously untreated patients with HER2‐amplified metastatic gastroesophageal adenocarcinoma [334].

Cytotoxic T‐lymphocyte‐associated antigen 4 (CTLA‐4) binds B7 molecules, thereby preventing their interaction with CD28 and inhibiting T‐cell activation [335]. Tremelimumab is an anti‐CTLA‐4 IgG2 monoclonal antibody. A Phase Ib/II study showed that the combination of durvalumab, tremelimumab, and paclitaxel was well tolerated and achieved an ORR of 52.1% in biomarker‐selected patients with metastatic GC.

Claudin 18.2 (CLDN18.2) is associated with GC cell invasion. CT041, a chimeric antigen receptor T‐cell (CAR‐T) therapy targeting CLDN18.2, demonstrated favorable ORR and DCR in patients with GC in a Phase I trial, and its efficacy did not appear to be affected by PD‐L1 expression or prior use of PD‐1/PD‐L1 inhibitors [336].

### 7.5 Macrophage‐Targeted Therapeutic Strategies

Given the key regulatory role of macrophages in GC progression, targeting macrophages has become an important therapeutic strategy. Studies have shown that promoting M1 polarization while inhibiting M2 polarization may contribute to the treatment of GC.

#### 7.5.1 Promoting M1 Polarization

GC cells exhibit decreased ADH1A expression. Therefore, restoring ADH1A expression in GC cells may enable ADH1A to modulate the cGMP‐PKG signaling pathway, thereby slowing GC progression and promoting IFN‐γ secretion, which polarizes macrophages toward the M1 phenotype [203]. This mechanism may have therapeutic potential in GC by promoting M1 polarization within the gastric TME.

miR‐125 is a proinflammatory miRNA that stimulates macrophages to secrete proinflammatory cytokines [337]. It has two subtypes: miR‐125a and miR‐125b [337]. LPS induces miR‐125a transcription in macrophages [338], whereas miR‐125b promotes M1 polarization by inhibiting IRF4 [339]. Both miR‐125a and miR‐125b potentiate NF‐κB signaling [340], thereby sustaining the M1 phenotype. Enhancing the expression of these miRNAs may be beneficial for GC treatment.

During liver fibrosis, follistatin‐like protein 1 (FSTL1) binds directly to PKM2, promoting PKM2 phosphorylation and nuclear translocation, reducing PKM2 ubiquitination, and inducing PKM2‐dependent glycolysis and M1 polarization [340]. This mechanism could be explored as a potential therapeutic strategy for GC because it may promote M1 polarization within the gastric TME and enhance antitumor immunity.

Flavokawain B inhibits GC cell proliferation and induces apoptosis through the TGF‐β/SMAD4 pathway while promoting M1 polarization and suppressing M2 polarization [341].

Astragalus polysaccharides activate Notch signaling and induce M1 polarization [342]. Their use may represent a strategy to promote M1 polarization in GC and thereby contribute to GC treatment.

IL‐24 is a downstream target of the IL‐6R/JAK/STAT pathway [343]. Cyclic GMP‐AMP synthase (cGAS)‐stimulator of interferon genes (STING) is a cytoplasmic DNA‐sensing pathway associated with the endoplasmic reticulum [344]. Expression of STING is significantly higher in cancer tissues than in normal tissues and is inversely correlated with patient survival. Both STING knockdown and STING activation reportedly shift TAMs toward the M1 phenotype, induce IL‐24 secretion through the IL‐6R/JAK/STAT pathway, and trigger GC cell apoptosis [345]. Given their roles in macrophage polarization and tumor suppression, IL‐24 and STING may represent potential therapeutic targets for GC.

#### 7.5.2 Killing M2 Macrophages

Trabectedin is an antitumor drug that induces depletion of M2 macrophages [346]. It promotes TNF‐related apoptosis by activating the TNF‐related apoptosis‐inducing ligand receptor (TRAILR), leading to caspase‐8–mediated macrophage apoptosis [347]. The use of trabectedin to reduce M2 macrophages in GC could be explored as a potential therapeutic strategy (Table 1).

**TABLE 1:** Drugs targeting M1/M2 macrophage polarization for GC treatment.

Drugs	Manufacturer	Study type	Targets	Mechanism of action	References
Trabectedin	PharmaMar	Preclinical	TRAIL‐Rs	Trabectedin activates caspase‐8 cleavage.	[346]
Bisphosphonates	−	Review	Fpp‐synthase	Bisphosphonates inhibits Fpp‐synthase.	[348]
M2pep	−	Preclinical	M2 TAMs	M2pep delivers proapoptotic peptides.	[349]
Melittin	−	Preclinical	M2 TAMs	Melittin reduces Mrc1/CD206 and VEGF levels.	[350]
PLX3397	Daiichi Sankyo	NCT01525602	CSF1R	PLX3397 Inhibits CSF1R.	[351]
Chlorogenic acid	Jiuzhang Biochemical Engineering Science and Technology Development Co., Ltd.	Preclinical	M1 and M2	Chlorogenic acid activates STAT1 and inhibits STAT6.	[352]
Ganodermanontriol	−	Preclinical	JAK1/STAT6 signaling pathway	Ganodermanontriol conducts micro‐PET imaging.	[353]
Synergy of CCL6, CpG, and anti‐IL‐10 receptor	MWG (Ebersberg, Germany)	Preclinical	Dendritic cells	The strategy triggers dendritic cells to secrete inflammatory cytokines.	[354]
poly(I:C)	GE Healthcare	Preclinical	TLR3	Poly(I:C) enhances TLR3‐involved IFN‐αβ signaling.	[355]
Bromonitrozine (RRx‐001)	EpicentRx, Inc	NCT02518958	NLRP3	Bromonitrozine (RRx‐001) suppresses CD47.	[356]
Pep‐20	MilliporeSigma	Preclinical	CD47	Pep‐20 blocks CD47–SIRPα interaction.	[357]
Corosolic acid	−	Preclinical	CD163	Corosolic acid suppresses STAT3.	[358]
*Dendrobium officinale* polysaccharide	Zhejiang Shouxiangu Pharmaceutical Co., Ltd. (Jinhua, China)	Preclinical	M2	*Dendrobium officinale* polysaccharide blocks STAT6/PPAR‐γ and JAGGED1/NOTCH1 pathways.	[359]
R848‐loaded β‐cyclodextrin nanoparticles (CDNP‐R848)	Selleckchem, MedchemExpress, InvivoGen, or LC Laboratories	Preclinical	M2	CDNP‐R848 activates TLR7/8.	[360]
TriCurin	Thermo Fisher Scientific	Preclinical	M2	TriCurin inhibits STAT3 and induces activation of STAT1 and NF‐κB.	[361]
Simvastatin	Melone Pharmaceutical Company	Preclinical	M2	Simvastatin downregulates LXR/ABCA1 expression.	[362]
IFN‐γ backpack	Thermo Fisher Scientific, Millipore Sigma	Preclinical	M2	IFN‐γ backpack releases IFN‐γ.	[363]
Chloroquine	Sigma‐Aldrich	Preclinical	M2	Chloroquine blocks lysosomal acidification.	[364]
IDR RP‐182	−	Preclinical	M2	IDR RP‐182 binds to CD206.	[365]
STING‐activating nanoparticles	−	Preclinical	M2	STING‐activating nanoparticles activates the STING pathway.	[366]
Self‐assembled M2pep‐Cs NPs/Plerixafor nanoparticles	MedChemExpress	Preclinical	CXCL12–CXCR4 signaling pathway	The strategy interferes with the homodimerization of HER‐2.	[367]
αVEGFR2‐MICA fusion antibodies	Eli Lilly and Company	Preclinical	AKT/ERK/P38 MAPK signaling pathway	The strategy inhibits FGFR phosphorylation.	[368]
Rapamycin and regorafenib	Dalian Meilun Biotechnology Co., Ltd. (Dalian, China)	Preclinical	M2 and PD‐L1/CD206‐overexpressing cancer cells	The strategy inhibits mTOR and angiogenesis.	[369]
Combination of pexidartinib and sirolimus	−	NCT02584647	TAMs	The strategy inhibits the CSF‐1/CSF‐1R pathway.	[370]

Bisphosphonates can be taken up by M2 TAMs, triggering macrophage death [348]. Liposomes and nanoparticles have been developed to encapsulate bisphosphonates, thereby reducing severe adverse effects and enhancing targeted uptake by M2 TAMs [371]. ClodroLip is an example of liposome‐encapsulated clodronate. Although bisphosphonates have demonstrated efficacy in the treatment of bone metastases [371], their potential application in GC treatment requires further investigation.

Targeted peptides can specifically bind M2 TAMs, leading to their depletion. Compared with antibodies, these peptides are less expensive and easier to synthesize, and they can also serve as therapeutic delivery vehicles [347]. M2pep selectively targets primary murine bone marrow‐derived M2 macrophages [349]. and serves as a delivery vehicle for KLA peptides, CSF1R inhibitors, and PLX3397 [351]. Notably, nanoparticles containing PLX3397 and M2pep have been shown to inhibit tumor growth in mice [351]. Therapeutic modifications of M2pep, including substitution of lysine at Position 9 or tryptophan at Position 10 with arginine or tyrosine, enhance its binding efficiency [372]. However, M2pep receptor expression varies among tumor types, with higher expression in 4T1 breast cancer cells than in liver cancer cells, limiting its broader applicability [373]. Moreover, its expression and therapeutic efficacy in GC remain uncertain and require further investigation.

The receptor for the linear UNO peptide is CD206. In GC, melanoma, and other tumors, the linear UNO peptide infiltrates the TME and selectively binds CD206‐positive M2 macrophages [374]. Melittin has a similar mechanism of action [374]. It has been shown to deplete M2 macrophages in mouse models of lung cancer and melanoma [350].

However, its specific effects on M2 macrophages in GC remain unknown and require further investigation.

It is worth noting that gastric adenosquamous carcinoma exhibits lower resistance to cisplatin, doxorubicin, and paclitaxel than patient‐derived organoids generated from the primary adenosquamous carcinoma tumor [375].

#### 7.5.3 Suppressing the Formation of M2 Macrophages

USP19 is overexpressed in GC cells. USP19 deficiency reduces chitin‐induced eosinophils, leading to decreased IL‐4 secretion, which inhibits M2 polarization [376]. IRF4 is essential for M2 macrophage transcription. USP19 stabilizes the NLRP3 inflammasome, preventing the interaction between IRF4 and P62/SQSTM1, thereby inhibiting IRF4 degradation and promoting M2 polarization [376]. By contrast, USP19 deletion prevents M2 polarization [376]. Thus, blocking USP19 in GC cells may promote IRF4 degradation and thereby inhibit M2 polarization in patients with GC. Additionally, adipocyte‐derived miR‐34a inhibits M2 polarization [377]. However, its role in M2 macrophages within the gastric TME requires further investigation.

In the TME, tumor cell‐secreted CSF‐1 binds to colony‐stimulating factor 1 receptor (CSF1R), recruiting macrophages to the tumor site and polarizing them toward the M2 phenotype [378]. PLX3397, a small‐molecule inhibitor, blocks CSF1R and thereby inhibits macrophage recruitment [379]. However, PLX3397 may cause anemia and hypertension and should be used with caution [380]. Potential therapeutic approaches include inhibiting CSF1R with PLX3397 or other small‐molecule inhibitors or suppressing CSF‐1 secretion to prevent M2 polarization of gastric macrophages.

miR‐378‐3p suppresses the AKT1 signaling pathway, thereby restricting IL‐4‐mediated M2 polarization [381]. The miR‐146 family includes miR‐146a and miR‐146b. miR‐146a functions as a tumor suppressor, and its deficiency leads to increased expression of proinflammatory cytokines, including TRAF6 and IL‐1 receptor‐associated kinase 1 (IRAK1) [382]. Both miR‐146a and miR‐146b inhibit NF‐κB activation, allowing macrophages to maintain the M2 phenotype [383]. Additionally, miR‐9, miR‐21, and miR‐147 also inhibit NF‐κB activation [337]. A potential therapeutic strategy may involve enhancing miR‐378‐3p expression to promote proinflammatory signaling while suppressing miR‐146, miR‐9, miR‐21, and miR‐147 expression to prevent polarization toward the M2 phenotype.

Pentraxin‐3 inhibits JNK1/2 phosphorylation in GC cells, thereby suppressing IL‐4 and IL‐10 expression and inhibiting M2 macrophage polarization [384]. Pentraxin‐3 may therefore have potential therapeutic applications in GC.

IL‐8 promotes GC cell proliferation and invasion. Under hypoxic conditions, IL‐8 activates the STAT1 pathway and enhances IL‐10 expression in GC cells, which in turn drives M2 macrophage polarization and further IL‐8 secretion [182]. This creates a positive feedback loop. Inhibiting IL‐8‐mediated activation of the STAT1 pathway may help disrupt this cascade, making IL‐8 a potential therapeutic target in GC. Additionally, lncRNA LOC339059 binds to c‐Myc in GC cells, blocking IL‐6 transcription and suppressing IL‐6/STAT3‐mediated M2 polarization [385].

CDK5RAP3 expressed by GC cells inhibits IL‐4 and IL‐10 expression by blocking NF‐κB phosphorylation in vivo, thereby suppressing M2 polarization. Additionally, CDK5RAP3 promotes M1 polarization both in vitro and in vivo. When CDK5RAP3 expression in GC cells is low, MMP2 released by M2 TAMs enhances GC cell invasion, metastasis, and EMT [386]. Increasing CDK5RAP3 expression in GC cells may represent a promising therapeutic strategy to counteract M2 polarization and inhibit GC progression.

Chlorogenic acid, also known as 5‐*O*‐caffeoylquinic acid, has been shown to treat glioblastoma by promoting M1 polarization and inhibiting M2 polarization through STAT1 activation and STAT6 suppression [352]. Its potential role in GC treatment remains to be verified and requires further experimental validation.

In cholesterol metabolism, cholesterol depletion leads to lipid raft dysfunction. In mice with metastatic ovarian cancer, tumor cell‐secreted hyaluronic acid induces membrane cholesterol efflux from TAMs, depleting lipid rafts and ultimately polarizing TAMs toward the M2 phenotype. Inhibiting hyaluronic acid secretion by GC cells may help prevent M2 polarization and thereby enhance GC treatment [387].

TRIM32 overexpressed in GC cells induces macrophage recruitment and M2 polarization through the TRIM32/PDE9A pathway [388]. Inhibiting TRIM32 expression in GC cells may suppress this process and help prevent tumor immune evasion.

GPX4 expressed in GC cells influences GC progression and metastasis. Downregulation of GPX4 in GC cells inhibits M2 polarization by reducing kynurenine levels [389].

Ganodermanontriol targeting the JAK/STAT6 axis inhibited M2 polarization in mice [353].

#### 7.5.4 Targeting M2 Macrophage‐Derived Factors in GC Progression

CTCs are associated with GC growth and metastasis. In colorectal cancer, M2 TAM‐secreted IL‐6 activates the JAK2/STAT3 pathway, suppressing miR‐506‐3p transcription and inducing ectopic expression of FOXQ1. This process enhances M2 TAM‐induced CCL2 secretion by colorectal cancer cells, promoting macrophage recruitment and mesenchymal CTC‐mediated colorectal cancer metastasis [187]. Therefore, blocking IL‐6 production by gastric M2 TAMs may represent a novel therapeutic strategy for GC.

M2 macrophages secrete IL‐4, which plays a crucial role in GC progression. High IL‐4 levels contribute to anti‐PD‐1 resistance in GC. Additionally, IL‐4 enhances M2 glycolysis and lactate production, leading to increased FcγRIIB expression and suppression of T‐cell immunity [390]. Furthermore, IL‐4‐induced transactivation of EGFR in heparin‐binding EGF‐like growth factor‐dependent macrophages promotes M2 polarization through STAT6 activation [391]. Thus, inhibiting IL‐4 expression may be a promising strategy for GC treatment.

Recent studies indicate that lactate produced during aerobic glycolysis promotes secretion of platelet factor 4 (PF4, also known as CXCL4) by M2 macrophages. PF4 binds to the IFN‐γ‐induced CXCR3 receptor on the surface of regulatory T cells, increasing the number of immunosuppressive Th1‐like regulatory T cells and facilitating tumor immune evasion. Knockout of PF4 in M2 macrophages or treatment with PF4‐targeted antibodies may inhibit tumor growth and exert antitumor effects [392], offering new opportunities for drug development and GC treatment.

#### 7.5.5 Conversion of M2 Into M1 Subtype

The activation state of tumor‐infiltrating macrophages can be modulated within the TME, allowing them to be redirected from a tumor‐promoting to an antitumor role. By contrast, tumor‐infiltrating dendritic cells exhibit defective proinflammatory cytokine production and fail to effectively induce innate IFN‐γ production or adaptive Th1 responses. CCL6 is an inflammatory chemokine, whereas IL‐10, an immunosuppressive cytokine, inhibits dendritic cell maturation and migration, thereby suppressing antitumor immune responses. Delivery of the CCL6 chemokine to tumor nodules using adenoviral vectors induces specific systemic antitumor responses and promotes substantial accumulation of dendritic cells at tumor sites. Additionally, the combination of CpG and anti‐IL‐10R reverses the functional paralysis of dendritic cells, promotes IL‐12 production, and induces conversion of M2 macrophages into the M1 phenotype [354]. Thus, adenoviral codelivery of CCL6, CpG, and anti‐IL‐10R may be a promising strategy for inducing M2‐to‐M1 conversion in GC.

Toll‐like receptors (TLRs) are innate immune receptors that bind specific ligands and activate M1 macrophage polarization [393]. Among them, TLR3 plays a crucial role in the conversion of M2 macrophages to the M1 phenotype [394]. Polyinosinic‐polycytidylic acid [poly(I:C)], a TLR3 ligand, stimulates TLR3 signaling and promotes M2‐to‐M1 conversion [355]. However, its role in GC therapy remains to be validated.

CD47 is a transmembrane protein that is overexpressed in most tumor cells. Signal regulatory protein alpha (SIRPα), the receptor for CD47, is expressed on macrophages, and the interaction between CD47 and SIRPα protects tumor cells from phagocytosis. Inhibiting CD47 signaling reprograms M2 macrophages into M1 macrophages, as observed with bromonitrozine (RRx‐001) [356]. Anti‐CD47 and anti‐SIRPα antibodies block the CD47/SIRPα axis, triggering M2‐to‐M1 repolarization and inducing tumor cell apoptosis [357, 395, 396]. However, anti‐CD47 antibodies may cause adverse effects because CD47 is widely expressed in normal tissues [357, 396]. By contrast, Pep‐20 offers a potentially safer alternative because it is a short peptide that blocks the CD47/SIRPα interaction without reducing the red blood cell count [357]. Thus, both anti‐CD47 antibodies and Pep‐20 could be explored as approaches for M2‐to‐M1 TAM repolarization, although the potential toxicities of anti‐CD47 antibodies require careful consideration.

CD40 is expressed on macrophages, whereas CD40L is expressed on T cells and B cells. Their interaction synergistically induces M2‐to‐M1 repolarization and stimulates TNF‐α secretion [397], thereby inhibiting the growth of GC cells.

The STAT6 and STAT3 pathways drive M2 macrophage polarization. PD‐1 deficiency blocks STAT6 signaling while enhancing STAT1 signaling, thereby suppressing M2 polarization and promoting M1 polarization [398]. Additionally, corosolic acid inhibits IL‐10 expression, thereby blocking STAT3 signaling and inducing M2‐to‐M1 repolarization [358]. Thus, inhibiting PD‐1 signaling and using corosolic acid to block IL‐10 may disrupt the STAT6/STAT3 pathways, facilitating repolarization of gastric M2 macrophages toward the M1 phenotype.

From the perspective of traditional Chinese medicine, Jianpi Yangzheng Xiaozheng Decoction promotes M2‐to‐M1 conversion [399]. It inhibits GC progression by suppressing exosomal PD‐L1 and reprogramming lipid metabolism through the SCD1/Wnt/β‐catenin pathway [400, 401]. Additionally, *Dendrobium officinale* polysaccharide enhances the mRNA expression of M1 markers such as IL‐6, CXCL9, and TNF‐α in M2 macrophages, thereby facilitating their conversion to the M1 phenotype, suggesting potential therapeutic value [359].

In Ehrlich ascites tumor‐bearing mice, combined treatment with 2‐deoxy‐D‐glucose (2‐DG) and RT successfully reprogrammed splenic macrophages from the M2 to the M1 phenotype [402]. However, the effect of 2‐DG on oxidative phosphorylation (OXPHOS) is dose‐dependent, promoting OXPHOS at low doses (< 1.25 mM) but inhibiting it at higher doses [403]. The potential application of this approach in GC treatment requires further evaluation.

Mannose‐conjugated lipid‐coated calcium phosphate nanoparticles can specifically deliver miR‐155 to M2 macrophages, inducing their conversion to the M1 phenotype [404]. However, the efficacy of these nanoparticles in promoting M2 repolarization within the gastric TME and their therapeutic potential in GC remain unclear and require further investigation.

Chimeric antigen receptor macrophages (CAR‐Ms), which are CAR‐expressing macrophages derived from induced pluripotent stem cells, can repolarize M2 macrophages into the M1 phenotype in an antigen‐dependent manner [405]. The use of the chimeric adenoviral vector Ad5.F35 addresses limitations associated with CAR‐M bioengineering. A clinical trial using Ad5.F35 targeting HER2 in solid tumors successfully induced M2‐to‐M1 repolarization [406]. CAR‐Ms retain the intrinsic properties of macrophages, including phenotypic plasticity and enhanced migratory capacity, and are regarded as an innovative immunotherapeutic approach [407, 408, 409, 410, 411].

In another study, IL‐4 polarized mouse bone marrow‐derived macrophages (BMDMs) to the M2 phenotype. These cells were subsequently treated with various immunostimulatory agents, and IL‐12 expression was measured to determine whether M2 macrophages had been repolarized to the M1 phenotype. R848 (resiquimod), a TLR7/8 agonist, significantly increased IL‐12 expression and effectively repolarized M2 macrophages to the M1 phenotype [412]. Unlike most immunostimulants, which require IFN‐γ to influence M1/M2 gene expression, R848 directly enhances IL‐12 expression [413]. R848 and other TLR agonists can be delivered to M2 macrophages in vivo using M2‐targeting peptides, enabling their repolarization to the M1 phenotype [414]. Combining R848 with nanoparticles has shown substantial effects on M2 repolarization. Gelatin nanoemulsions loaded with R848 (resiquimod‐GNE) produced sustained M2‐to‐M1 repolarization and significantly enhanced expression of proinflammatory markers (e.g., CD80) and antitumor activity [415]. Additionally, polyglucose nanoparticles (macrins), which exhibit high macrophage affinity, enhanced the effect of R848 on primary mouse macrophages by threefold in vitro [413, 416, 417, 418]. R848‐loaded β‐cyclodextrin nanoparticles (CDNP‐R848) induced high IL‐12 production in macrophages and successfully repolarized M2 macrophages to the M1 phenotype across various mouse tumor models, effectively slowing tumor growth [360, 419]. Furthermore, R848 and its derivatives, including R848‐loaded nanoparticles, were reported to be noncytotoxic to both macrophages and tumor cells [419, 420, 421]. Thus, R848 may represent a promising therapeutic strategy for GC.

TriCurin, a synergistic formulation of curcumin, resveratrol, and epicatechin gallate, inhibits STAT3 signaling in M2 macrophages within TC‐1 tumors while activating STAT1 and NF‐κB, thereby repolarizing M2 macrophages to the M1 phenotype [361]. Such synergistic formulations may represent a potential therapeutic strategy for GC.

Simvastatin reduces the expression of CD206 and TGF‐β, a protumor cytokine, by downregulating liver X receptor (LXR)/ATP‐binding cassette transporter A1 (ABCA1) signaling in the hepatic TME, thereby promoting M2‐to‐M1 repolarization [362]. Its potential role in GC treatment warrants further investigation.

Floppy disk‐like granular “backpacks” have been shown to modulate macrophage phenotypes in vivo. IFN‐γ backpacks bind tightly to macrophages, providing sustained IFN‐γ stimulation. An animal study demonstrated successful repolarization of M2 macrophages into the M1 phenotype in mice treated with IFN‐γ backpacks [363]. However, their effectiveness in GC remains uncertain and requires further investigation.

Chloroquine, an autophagy inhibitor, has been shown to influence M2 macrophage polarization. In Hep‐2 laryngeal tumor cells, chloroquine promotes LC3‐II accumulation by inhibiting lysosomal acidification and autophagosomal degradation in M2 macrophages [364], thereby suppressing M2 macrophage autophagy. Additionally, chloroquine repolarizes M2 macrophages to the M1 phenotype by upregulating major histocompatibility complex Class II expression and downregulating CD206 expression [422]. Given these effects, chloroquine may have therapeutic potential in GC.

Innate defense regulator (IDR) peptides are synthetic mimics of natural antimicrobial/host defense peptides that function as immunomodulators [423]. IDR RP‐182 activates CD206 on M2 macrophages, triggering endocytosis, phagosome–lysosome formation, M2 autophagy, and M2‐to‐M1 reprogramming [365]. The therapeutic potential of IDR RP‐182 in GC remains to be investigated.

The macrophage receptor with collagenous structure (MARCO) is a scavenger receptor that is overexpressed on the surface of M2 macrophages [424]. Anti‐MARCO antibodies block inhibitory Fc receptors on M2 macrophages, reprogramming them into the M1 phenotype [425].

STING is a cytoplasmic DNA sensor that is essential for the transcription of host defense‐associated genes. Activation of STING by agonists triggers signaling pathways that repolarize M2 macrophages into the M1 phenotype, thereby contributing to antitumor immunity [426]. Additionally, STING‐activating nanoparticles enhance the immunostimulatory activity of cyclic GMP‐AMP (cGAMP), thereby augmenting STING signaling [366].

Given its role in macrophage reprogramming, STING may represent a promising therapeutic target for GC.

miR‐221‐3p suppresses the JAK3/STAT3 signaling pathway, leading to M2‐to‐M1 repolarization [427]. Further studies are needed to determine whether this miRNA has therapeutic potential in GC.

Methionine enkephalin is an endogenous opioid peptide. It repolarizes M2 macrophages into the M1 phenotype and induces GC cell apoptosis through the opioid growth factor receptor (OGFr)/PI3K/AKT/mTOR signaling pathway [428].

Self‐assembled M2pep‐Cs NPs/Plerixafor nanoparticles repolarized M2 macrophages into M1 macrophages and inhibited tumor progression by targeting the CXCL12‐CXCR4 signaling pathway [367].

αVEGFR2‐MICA fusion antibodies repolarized M2 macrophages into M1 macrophages in mice by targeting the extracellular domain of VEGFR2 on macrophages [368].

#### 7.5.6 Metabolic Reprogramming of M2 Macrophages

Metabolic reprogramming plays a critical role in macrophage repolarization. In CT26 colon cancer cells and colorectal tumor models, a dual‐targeted liposomal delivery system containing the mTOR inhibitor rapamycin and the antiangiogenic agent regorafenib successfully reprogrammed M2 macrophages into the M1 phenotype by remodeling glycolytic metabolism and reducing lactate production through the mTOR pathway [369]. This liposomal system may have therapeutic potential for GC.

Glutamine synthetase (GS) promotes M2 macrophage polarization. By contrast, L‐methionine sulfoximine inhibits GS, leading to succinate accumulation and increased glucose utilization. This process suppresses IL‐10‐mediated CD163 expression in M2 macrophages, thereby facilitating M2‐to‐M1 repolarization. Additionally, *GS* gene deletion in M2 macrophages has been shown to repolarize M2 macrophages into the M1 phenotype [429]. The use of L‐methionine sulfoximine to inhibit GS and metabolically reprogram M2 macrophages may represent a potential therapeutic strategy for GC.

CSF1R inhibitors (e.g., pexidartinib combined with sirolimus) inhibit tumor growth by inducing lipid metabolic reprogramming of M2 macrophages [370]. Reprogramming lipid metabolism by targeting cholesterol transfer with CSF1R inhibitors or by inhibiting M2 macrophages may represent an effective therapeutic strategy for GC [370, 430].

### 7.6 Preclinical and Clinical Progress of GC

In recent years, several therapeutic strategies have gradually progressed from in vitro proof‐of‐concept studies to validation in animal models, and some have advanced to clinical trials.

Trastuzumab significantly inhibited tumor growth and was associated with HER2 copy number in patient‐derived gastric adenocarcinoma xenograft mouse models [431]. Futibatinib, a fibroblast growth factor receptor (FGFR) 1–4 inhibitor, achieved an ORR of 17.9% in a Phase II study of patients with GC or gastroesophageal junction cancer [432]. Zolbetuximab combined with CAPOX, targeting CLDN18.2, improved OS in patients with GC or gastroesophageal junction adenocarcinoma in the Phase III GLOW trial [433]. Infigratinib showed an ORR of 23.8% in a Phase II trial for patients with FGFR2‐amplified GC [434]. K13 synergized with docetaxel overcame docetaxel resistance in preclinical models and achieved partial response (PR) in a Phase Ib/IIa trial [435]. Ramucirumab, an anti‐VEGFR‐2 antibody, is the global standard second‐line treatment for GC based on REGARD and RAINBOW [436]. In the phase‐in‐human Phase I trial, telisotuzumab adizutecan (ABBV‐400/Temab‐A), targeting c‐MET, achieved an ORR of 29%, median PFS of 4 months and median OS of 5.82 months in heavily pretreated advanced gastric/gastroesophageal junction adenocarcinoma, with manageable safety in patients [437]. In the Phase II trial, maintenance rucaparib after first‐line platinum‐based chemotherapy in HER2‐negative advanced esophagogastric adenocarcinoma achieved a median PFS of 4.2 months, which did not have a significant difference [438] (Table 2).

**TABLE 2:** Preclinical studies and clinical trials of therapeutic strategies for GC.

Strategy	Research phase	Pathway	Mechanism	References
Trastuzumab	Preclinical	HER‐2	Trastuzumab interferes with the homodimerization of HER‐2 and activates antibody‐dependent cytotoxicity (ADCC)	[431]
Futibatinib	Phase II	FGFR	Futibatinib inhibits FGFR phosphorylation and blocks RAS/MEK/ERK and PI3K/AKT axis	[432]
Zolbetuximab combined with capecitabine and oxaliplatin (CAPOX)	Phase III	CLDN18.2	The strategy activates ADCC and complement‐dependent cytotoxicity (CDCC)	[433]
Infigratinib	Phase II	FGFR2 tyrosine kinase	Infigratinib inhibits FGFR2 kinase activity and blocks RAS/MEK/ERK and PI3K/AKT axes	[434]
K13 synergized with docetaxel	Phase Ib/II	Ribonucleotide Reductase M2 (RRM2)	The strategy inhibits RRM2 to block the AKT/mTOR axis to induce ferroptosis	[435]
Ramucirumab	Approved	VEGFR2	Ramucirumab binds to VEGFR2, preventing VEGF‐A/C/D from binding to VEGFR2	[436]
Telisotuzumab Adizutecan (ABBV‐400/Temab‐A)	Phase I	Mesenchymal–epithelial transition factor (c‐MET)	The strategy binds with c‐MET and releases a topoisomerase 1 inhibitor payload	[437]
Rucaparib	Phase II	PARP	Rucaparib blocks PARP‐mediated repair of DNA and increases PARP‐DNA complexes	[438]

Abbreviations: ADCC, antibody‐dependent cytotoxicity; CAPOX, capecitabine and oxaliplatin; CDCC, complement‐dependent cytotoxicity; GC, gastric cancer.

## 8 Conclusions and Future Perspectives

### 8.1 Summary of Key Insights

This review systematically covers the entire spectrum of GC, from epidemiology to clinical management, including epidemiology, risk factors, pathogenesis, clinical features, diagnosis, and management. Regarding pathogenesis, we elaborate on the classical Correa cascade and focus on the role of miRNAs in regulating invasion, proliferation, apoptosis, metastasis, and drug resistance. We also highlight the bidirectional interactions between GC cells and macrophages that perpetuate tumor progression within the TME.

We systematically reviewed current early screening methods and risk‐stratification tools, classification systems, endoscopic findings, and biomarkers. In terms of treatment, in addition to surgery, chemotherapy, and RT, we summarize targeted therapies, including anti‐HER2 therapy (trastuzumab), antiangiogenic therapy (ramucirumab), immune checkpoint inhibitors (PD‐1/PD‐L1 inhibitors), and therapeutic strategies based on macrophage phenotypic repolarization and metabolic reprogramming.

Therapeutic strategies targeting macrophage phenotypic repolarization and metabolic reprogramming are emerging as a frontier in GC research. Macrophage repolarization essentially involves intervening in the complex signaling networks within the TME. The goal of macrophage repolarization therapy is to reprogram protumor M2 macrophages into antitumor M1 macrophages. The basis of this strategy lies in reversing the processes by which GC cells induce M2 polarization. GC cells promote M2 polarization through multiple mechanisms, including exosome release, chemokine secretion, and metabolic regulation. Together, these mechanisms establish a signaling network that maintains macrophages in a protumor state. Regulating these signaling molecules, targeting key signaling nodes, or delivering specific therapeutic agents can inhibit these pathways or activate alternative pathways, thereby inducing macrophage repolarization from the M2 to the M1 phenotype. Metabolic reprogramming is not merely a consequence of macrophage polarization but also a determinant of macrophage functional state. In recent years, research has progressed from describing metabolic phenotypes to exploring their underlying mechanisms and therapeutic applications. The therapeutic potential of metabolic reprogramming lies in altering macrophage fate by targeting key metabolic pathways. Modulating macrophage metabolism can differentially affect metabolite production, thereby influencing macrophage phenotype and GC progression.

### 8.2 Current Limitations and Unmet Needs

GC exhibits substantial heterogeneity in anatomical location, histological type, molecular subtype, and regional distribution. These differences contribute to distinct pathogeneses, clinical behaviors, and treatment responses. Although this review discusses several GC subtypes, the broad scope of the review inevitably limits detailed discussion of subtype‐specific characteristics that may influence clinical diagnosis and treatment.

This review presents a large body of evidence from both basic research (e.g., studies of miRNAs and metabolism) and clinical observations. However, the translational gap between these findings remains substantial. Most studies investigating molecular mechanisms are still limited to cellular or preclinical models, and early‐phase clinical trials remain scarce.

Metabolic pathways are highly interconnected. Inhibiting a single pathway (e.g., glycolysis) may result in compensatory activation of alternative pathways, thereby reducing therapeutic efficacy. Furthermore, many metabolic pathways are essential for the normal function and proliferation of healthy cells. Consequently, systemic metabolic inhibition may cause significant toxicity.

Macrophages do not function in isolation. Their activities are closely coordinated with those of other cells within the TME, including CAFs and T cells. Reprogramming macrophages without considering these interactions may disrupt beneficial cellular crosstalk or inadvertently enhance other immunosuppressive mechanisms, ultimately contributing to therapeutic resistance.

The treatment landscape for GC is evolving rapidly, with new clinical trial results emerging continuously, particularly in the field of immune checkpoint inhibitors. Although this review aims to incorporate the latest advances, the inherent delay associated with academic publication means that some recently published, potentially important clinical trial data may not have been included.

### 8.3 Future Directions and Translational Outlook

In the future, molecular classification may be more deeply integrated with screening strategies for high‐risk populations to facilitate earlier detection of GC and more effective monitoring of recurrence, while supporting the development of more refined guidelines for endoscopic surveillance and preventive interventions.

Deep learning models and artificial intelligence may integrate multimodal data, including genomic, proteomic, metabolomic, pathological imaging, and clinical information, to construct more accurate models for predicting treatment response and prognosis, thereby assisting clinical decision‐making.

Large‐scale clinical studies investigating macrophage repolarization and metabolic reprogramming in patients with GC are needed to provide high‐quality clinical evidence and determine whether these strategies can translate into meaningful survival benefits.

## Author Contributions

**Yixuan Xin, Xingya Wang, and Yangtao Wu**: writing – original draft. **Qian Liu, Qian Ren, and Hui Wang**: writing – review and editing. **Jingbo Zhai**: conceptualization, project administration, writing – review and editing. All the authors have read and approved the final manuscript.

## Funding

This work was supported by the grant from the National Natural Science Foundation of China (No. 82160312), and 2024 Local Universities' Scientific Research Platform Project Supported by Central Government—Key Laboratory Project for Higher Universities of Inner Mongolia Autonomous Region, and Basic Scientific Research Project for Universities of Inner Mongolia Autonomous Region—Capacity Building Project of Scientific Research and Innovation Platform of Brucellosis Prevention and Treatment Engineering Technology Research Center (No. NMDZK24002), and 2025 Finance Project of Inner Mongolia Autonomous Region—Special Fund of Science and Engineering, and Major Science and Technology projects of Inner Mongolia of China (2019ZD006), and Postgraduate Education and Teaching Reform Project of Inner Mongolia Autonomous Region (JG2024045C).

## Ethics Statement

The authors have nothing to report.

## Conflicts of Interest

The authors declare no conflicts of interest.

## Data Availability

The authors have nothing to report.

## References

[1] H. Sung , J. Ferlay , R. L. Siegel , et al., “Global Cancer Statistics 2020: GLOBOCAN Estimates of Incidence and Mortality Worldwide for 36 Cancers in 185 Countries,” CA: A Cancer Journal for Clinicians 71, no. 3 (2021): 209–249. 10.3322/caac.21660 33538338

[2] U. B. Ahmedov , “Epidemiology of Gastric Cancer: Global Burden, Risk Factors, and Trends,” Modern Science and Research 5, no. 6 (2026): 563–572.

[3] T. Zhang , Y. Zhang , and X. Leng , “Global, Regional, and National Trends in Gastric Cancer Burden: 1990‐2021 and Projections to 2040,” Frontiers in Oncology 14 (2024): 1468488. 10.3389/fonc.2024.1468488 PMC11669584 39726708

[4] J. Y. Park , D. Georges , C. J. Alberts , F. Bray , G. Clifford , and I. Baussano , “Global Lifetime Estimates of Expected and Preventable Gastric Cancers Across 185 Countries,” Nature Medicine 31, no. 9 (2025): 3020–3027. 10.1038/s41591-025-03793-6 PMC12443596 40624406

[5] J. L. Liang , H. M. Yuan , C. Quan , and J. Q. Chen , “Risk Factors for Gastric Cancer: An Umbrella Review of Systematic Reviews and Meta‐Analyses,” Frontiers in Oncology 15 (2025): 1564575. 10.3389/fonc.2025.1564575 PMC12241803 40641920

[6] Y. He , Q. Hong , S. Chen , J. Zhou , and S. Qiu , “Reprogramming Tumor‐Associated Macrophages in Gastric Cancer: A Pathway to Enhanced Immunotherapy,” Frontiers in Immunology 16 (2025): 1558091. 10.3389/fimmu.2025.1558091 PMC11911521 40098971

[7] X. Xiang , F. Wang , Y. Shen , and S. Dai , “CLEVER‐1 Blockade Reprograms TAMs to Overcome Anti‐PD‐1 Resistance in Gastric Cancer,” Journal for ImmunoTherapy of Cancer 13, no. 12 (2025): e013145. 10.1136/jitc-2025-013145 PMC12682175 41339106

[8] M. Yuan , X. Zheng , and S. Zheng , “Exosomal PKM2: A Noninvasive Diagnostic Marker Linking Macrophage Metabolic Reprogramming to Gastric Cancer Pathogenesis,” Cancer Science 116, no. 6 (2025): 1537–1549. 10.1111/cas.70056 PMC12127107 40126044

[9] J. Ren , P. Lu , and X. Zhou , “Genome‐Scale Methylation Analysis of Circulating Cell‐Free DNA in Gastric Cancer Patients,” Clinical Chemistry 68, no. 2 (2022): 354–364. 10.1093/clinchem/hvab204 34791072

[10] Y. Zhang , X. Xu , and S. Wang , “Fecal Microbiota Transplantation Combined With Anti‐PD‐1 Therapy in Refractory Microsatellite‐Stable Gastric Cancer: A Phase I Feasibility and Safety Study,” Journal for ImmunoTherapy of Cancer 14, no. 3 (2026): e013823. 10.1136/jitc-2025-013823 PMC13034380 41871875

[11] A. K. Patel , N. S. Sethi , and H. Park , “Gastric Cancer,” JAMA 335, no. 5 (2026): 439. 10.1001/jama.2025.20034 41499132

[12] A. P. Thrift , T. N. Wenker , and H. B. El‐Serag , “Global Burden of Gastric Cancer: Epidemiological Trends, Risk Factors, Screening and Prevention,” Nature Reviews Clinical Oncology 20, no. 5 (2023): 338–349. 10.1038/s41571-023-00747-0 36959359

[13] W. Liu , Z. Z. Peng , T. Zhang , K. Liao , and J. Li , “Burden, Trends, Driving Factors, and Predictions of Gastric Cancer: A Cross‐National Comparative Analysis of China, Japan, and South Korea,” BMC Cancer 25, no. 1 (2025): 1669. 10.1186/s12885-025-15009-8 PMC12574057 41163138

[14] N. Qin , Y. Fan , T. Yang , Z. Yang , and D. Fan , “The Burden of Gastric Cancer and Possible Risk Factors From 1990 to 2021, and Projections Until 2035: Findings From the Global Burden of Disease Study 2021,” Biomarker Research 13, no. 1 (2025): 5. 10.1186/s40364-024-00720-8 PMC11708091 39773334

[15] M. Inoue , “Epidemiology of Gastric Cancer—Changing Trends and Global Disparities,” Cancers 16, no. 17 (2024): 2948. 10.3390/cancers16172948 PMC11394435 39272806

[16] M. Capasso , T. Pessarelli , G. Manfredi , and A. Amato , “Early Detection of Gastric Cancer: Linking Epidemiology, Pathophysiology, and Innovations in Digestive Endoscopy,” Diseases 14, no. 4 (2026): 148. 10.3390/diseases14040148 PMC13115024 42041640

[17] Y. Li , M. Tang , and S. Li , “Global, Regional, and National Burden of Gastrointestinal Cancers in Early Onset and Young Women, 1990–2021: Analysis of Data From the Global Burden of Disease Study 2021,” Journal of Gastrointestinal Oncology 16, no. 5 (2025): 1924–1943. 10.21037/jgo-2025-229 PMC12598334 41220740

[18] N. Jiang , “Global Burden and Future Trends of Gastric Cancer in Women of Reproductive Age: Estimates From the GBD 2021 Study, 1990–2050,” Frontiers in Oncology 15 (2025): 1616936. 10.3389/fonc.2025.1616936 PMC12661426 41323395

[19] S. F. Alavi Rostami , M. Khaledi , and F. Dalilian , “Vacuolating Cytotoxin A (VacA) and Extracellular Vesicles in *Helicobacter pylori*: Two Key Arms in Disease Development,” Iranian Journal of Pathology 20, no. 1 (2025): 1–17. 10.30699/ijp.2024.2031417.3312 PMC11887638 40060221

[20] O. Handa , Y. Naito , and T. Yoshikawa , “CagA Protein of *Helicobacter pylori*: A Hijacker of Gastric Epithelial Cell Signaling,” Biochemical Pharmacology 73, no. 11 (2007): 1697–1702. 10.1016/j.bcp.2006.10.022 17134680

[21] S. Ansari and N. Ahmed , “Pathogenicity of *Helicobacter pylori*‐Associated Gastric Cancer,” World Journal of Clinical Oncology 16, no. 12 (2025): 110909. 10.5306/wjco.v16.i12.110909 PMC12754107 41480161

[22] A. Lamb and L. F. Chen , “The Many Roads Traveled by *Helicobacter pylori* to NF‐κB Activation,” Gut Microbes 1, no. 2 (2010): 109–113. 10.4161/gmic.1.2.11587 PMC3023589 21326919

[23] S. Backert and M. Naumann , “What a Disorder: Proinflammatory Signaling Pathways Induced by *Helicobacter pylori* ,” Trends in Microbiology 18, no. 11 (2010): 479–486. 10.1016/j.tim.2010.08.003 20863705

[24] K. Matsushima , H. Isomoto , and N. Inoue , “MicroRNA Signatures in *Helicobacter pylori*‐Infected Gastric Mucosa,” International Journal of Cancer 128, no. 2 (2011): 361–370. 10.1002/ijc.25348 20333682

[25] X. Chen , B. Zhou , and S. Wang , “Intestinal Metaplasia Key Molecules and UPP1 Activation via *Helicobacter pylori*/NF‐kB: Drivers of Malignant Progression in Gastric Cancer,” Cancer Cell International 24, no. 1 (2024): 399. 10.1186/s12935-024-03598-6 PMC11657005 39695769

[26] Y. Y. Wu , C. W. Lin , and K. S. Cheng , “Increased Programmed Death‐Ligand‐1 Expression in Human Gastric Epithelial Cells in *Helicobacter pylori* Infection,” Clinical and Experimental Immunology 161, no. 3 (2010): 551–559. 10.1111/j.1365-2249.2010.04217.x PMC2962974 20646001

[27] X. Wang , Y. Weng , Y. Yao , S. Shao , L. Mao , and J. Wang , “ *Helicobacter pylori* Modulates Macrophage Polarization via the Glucocorticoid Receptor to Promote Gastric Cancer Cell Proliferation,” Tissue and Cell 96 (2025): 103001. 10.1016/j.tice.2025.103001 40472550

[28] Y.‐S. Teng , Y.‐L. Zhao , and M.‐S. Li , “Upexpression of BHLHE40 in Gastric Epithelial Cells Increases CXCL12 Production Through Interaction With p‐STAT3 in *Helicobacter pylori*‐Associated Gastritis,” FASEB Journal 34, no. 1 (2020): 1169–1181. 10.1096/fj.201900464RR 31914631

[29] C. Mi , Y. Zhao , L. Ren , and D. Zhang , “Inhibition of MDFI Attenuates Proliferation and Glycolysis of *Helicobacter pylori*‐Infected Gastric Cancer Cells by Inhibiting Wnt/β‐Catenin Pathway,” Cell Biology International 46, no. 12 (2022): 2198–2206. 10.1002/cbin.11907 36116024

[30] R. Cao , F. Zhou , C. Zhu , and H. Xu , “Metabolic Reprogramming as a Key Regulator in *Helicobacter pylori*‐Infected Gastric Cancer,” Gastric Cancer 29, no. 1 (2026): 1–15. 10.1007/s10120-025-01675-x PMC12830420 41171531

[31] X. Chen , R. Lu , and S. Wang , “ *Helicobacter pylori* Promotes Gastric Cancer Progression via UPP1‐Mediated Uridine Bypass of Glycolysis,” Cancer Cell International 26, no. 1 (2026): 91. 10.1186/s12935-026-04195-5 PMC12910853 41572295

[32] N. Kim , W.‐Y. Park , and J. M. Kim , “Gene Expression of AGS Cells Stimulated With Released Proteins by *Helicobacter pylori* ,” Journal of Gastroenterology and Hepatology 23, no. 4 (2008): 643–651. 10.1111/j.1440-1746.2007.05241.x 18070016

[33] C. Shen , H. Liu , and Y. Chen , “ *Helicobacter pylori* Induces GBA1 Demethylation to Inhibit Ferroptosis in Gastric Cancer,” Molecular and Cellular Biochemistry 480, no. 3 (2025): 1845–1863. 10.1007/s11010-024-05105-x 39283563

[34] K. Fu , A. H. K. Cheung , and C. C. Wong , “ *Streptococcus anginosus* Promotes Gastric Inflammation, Atrophy, and Tumorigenesis in Mice,” Cell 187, no. 4 (2024): 882–896.e17, e17. 10.1016/j.cell.2024.01.004 38295787

[35] F. Liao , J. Rong , and C. He , “ *Streptococcus anginosus*‐Generated Succinate Promotes the Progression of Gastric Cancer via the Succinate/SUCNR1/ABRAXAS1 Axis,” Cell Reports 45, no. 3 (2026): 117047. 10.1016/j.celrep.2026.117047 41746808

[36] R. Yang , Y. Yang , W. Lin , K. He , D. Bi , and L. Teng , “ *Lactobacillus paracasei* ZJUZ2‐3 Inhibits Gastrointestinal Tumors via the IAA‐Induced AHR/MTDH/NF‐κB Axis,” International Journal of Biological Sciences 21, no. 14 (2025): 6522–6541. 10.7150/ijbs.114602 PMC12594602 41208900

[37] P. Sonveaux , T. Copetti , and C. J. De Saedeleer , “Targeting the Lactate Transporter MCT1 in Endothelial Cells Inhibits Lactate‐Induced HIF‐1 Activation and Tumor Angiogenesis,” PLoS ONE 7, no. 3 (2012): e33418. 10.1371/journal.pone.0033418 PMC3302812 22428047

[38] K. Vinasco , H. M. Mitchell , N. O. Kaakoush , and N. Castaño‐Rodríguez , “Microbial Carcinogenesis: Lactic Acid Bacteria in Gastric Cancer,” Biochimica et Biophysica Acta (BBA) ‐ Reviews on Cancer 1872, no. 2 (2019): 188309. 10.1016/j.bbcan.2019.07.004 31394110

[39] R. Peng , S. Liu , and W. You , “Gastric Microbiome Alterations Are Associated With Decreased CD8+ Tissue‐Resident Memory T Cells in the Tumor Microenvironment of Gastric Cancer,” Cancer Immunology Research 10, no. 10 (2022): 1224–1240. 10.1158/2326-6066.CIR-22-0107 35881964

[40] M. Sun , W. Wu , and L. Chen , “Microbiota‐Derived Short‐Chain Fatty Acids Promote Th1 Cell IL‐10 Production to Maintain Intestinal Homeostasis,” Nature Communications 9, no. 1 (2018): 3555. 10.1038/s41467-018-05901-2 PMC6120873 30177845

[41] S. R. Kim , K. Kim , and S. A. Lee , “Effect of Red, Processed, and White Meat Consumption on the Risk of Gastric Cancer: An Overall and Dose–Response Meta‐Analysis,” Nutrients 11, no. 4 (2019): 826. 10.3390/nu11040826 PMC6520977 30979076

[42] V. Balendra , C. Amoroso , and B. Galassi , “High‐Salt Diet Exacerbates *H. pylori* Infection and Increases Gastric Cancer Risks,” Journal of Personalized Medicine 13, no. 9 (2023): 1325. 10.3390/jpm13091325 PMC10533117 37763093

[43] W. Hatta , T. Koike , and N. Asano , “The Impact of Tobacco Smoking and Alcohol Consumption on the Development of Gastric Cancers,” International Journal of Molecular Sciences 25, no. 14 (2024): 7854. 10.3390/ijms25147854 PMC11276971 39063094

[44] M. Dalmartello , F. Turati , and Z. F. Zhang , “Allium Vegetables Intake and the Risk of Gastric Cancer in the Stomach Cancer Pooling (StoP) Project,” British Journal of Cancer 126, no. 12 (2022): 1755–1764. 10.1038/s41416-022-01750-5 PMC9174191 35210588

[45] H. Akhondi , E. A. Kalteh , and M. H. Lotfi , “Common Risk Factors in Gastrointestinal Cancers: A Narrative Review,” Medical Principles and Practice 34, no. 6 (2025): 509–526. 10.1159/000547241 PMC12503528 40628241

[46] Y. Chen , R. Fu , M. Xu , Y. Huang , G. Sun , and L. Xu , “N‐Methyl‐N‐Nitro‐N‐Nitrosoguanidine‐Mediated ING4 Downregulation Contributed to the Angiogenesis of Transformed Human Gastric Epithelial Cells,” Life Sciences 199 (2018): 179–187. 10.1016/j.lfs.2018.02.034 29496496

[47] A. Vences‐Mejía , H. Caballero‐Ortega , and V. Dorado‐González , “Cytochrome P450 Expression in Rat Gastric Epithelium With Intestinal Metaplasia Induced by High Dietary NaCl Levels,” Environmental Toxicology and Pharmacology 20, no. 1 (2005): 57–64. 10.1016/j.etap.2004.10.010 21783568

[48] K. Ozeki , K. Hada , and Y. Wakiya , “Factors Influencing the Degree of Gastric Atrophy in *Helicobacter pylori* Eradication Patients With Drinking Habits,” Microorganisms 12, no. 7 (2024): 1398. 10.3390/microorganisms12071398 PMC11278706 39065166

[49] S. Syngal , R. E. Brand , J. M. Church , F. M. Giardiello , H. L. Hampel , and R. W. Burt , “ACG Clinical Guideline: Genetic Testing and Management of Hereditary Gastrointestinal Cancer Syndromes,” American Journal of Gastroenterology 110, no. 2 (2015): 223–262, quiz 263. 10.1038/ajg.2014.435 PMC4695986 25645574

[50] W. Yang , T. Yu , and X. Huang , “Intestinal Microbiota‐Derived Short‐Chain Fatty Acids Regulation of Immune Cell IL‐22 Production and Gut Immunity,” Nature Communications 11, no. 1 (2020): 4457. 10.1038/s41467-020-18262-6 PMC7478978 32901017

[51] M. Luu , Z. Riester , and A. Baldrich , “Microbial Short‐Chain Fatty Acids Modulate CD8+ T Cell Responses and Improve Adoptive Immunotherapy for Cancer,” Nature Communications 12, no. 1 (2021): 4077. 10.1038/s41467-021-24331-1 PMC8249424 34210970

[52] A. Sivan , L. Corrales , and N. Hubert , “Commensal *Bifidobacterium* Promotes Antitumor Immunity and Facilitates Anti–PD‐L1 Efficacy,” Science 350, no. 6264 (2015): 1084–1089. 10.1126/science.aac4255 PMC4873287 26541606

[53] Z. Wang , X. Zhang , and L. Zhu , “Inulin Alleviates Inflammation of Alcoholic Liver Disease via SCFAs‐Inducing Suppression of M1 and Facilitation of M2 Macrophages in Mice,” International Immunopharmacology 78 (2020): 106062. 10.1016/j.intimp.2019.106062 31830621

[54] Y. Pi , Y. Wu , and X. Zhang , “Gut Microbiota‐Derived Ursodeoxycholic Acid Alleviates Low Birth Weight‐Induced Colonic Inflammation by Enhancing M2 Macrophage Polarization,” Microbiome 11, no. 1 (2023): 19. 10.1186/s40168-022-01458-x PMC9887892 36721210

[55] Z. Zhao , L. Zhong , and P. Li , “Cholesterol Impairs Hepatocyte Lysosomal Function Causing M1 Polarization of Macrophages via Exosomal miR‐122‐5p,” Experimental Cell Research 387, no. 1 (2020): 111738. 10.1016/j.yexcr.2019.111738 31759057

[56] Y. Li , J. Yan , M. Wang , J. Lv , F. Yan , and J. Chen , “Uremic Toxin Indoxyl Sulfate Promotes Proinflammatory Macrophage Activation by Regulation of β‐Catenin and YAP Pathways,” Journal of Molecular Histology 52, no. 2 (2021): 197–205. 10.1007/s10735-020-09936-y PMC8012310 33387144

[57] H. Song , Y. Zhang , J. Liu , W. Liu , and B. Luo , “Activation of DNA Methyltransferase 3A by Epstein‐Barr Nuclear Antigen 1 in Gastric Carcinoma,” Digestive and Liver Disease 54, no. 7 (2022): 973–983. 10.1016/j.dld.2021.06.004 34215536

[58] D. Nkosi , L. Sun , L. C. Duke , and D. G. Meckes Jr , “Epstein‐Barr Virus LMP1 Manipulates the Content and Functions of Extracellular Vesicles to Enhance Metastatic Potential of Recipient Cells,” PLoS Pathogens 16, no. 12 (2020): e1009023. 10.1371/journal.ppat.1009023 PMC7774862 33382850

[59] W. Li , A. Okabe , and G. Usui , “Activation of EHF via STAT3 Phosphorylation by LMP2A in Epstein‐Barr Virus–Positive Gastric Cancer,” Cancer Science 112, no. 8 (2021): 3349–3362. 10.1111/cas.14978 PMC8353921 34014591

[60] M. Fukuda and R. Longnecker , “Latent Membrane Protein 2A Inhibits Transforming Growth Factor‐β1‐Induced Apoptosis Through the Phosphatidylinositol 3‐Kinase/Akt Pathway,” Journal of Virology 78, no. 4 (2004): 1697–1705. 10.1128/JVI.78.4.1697-1705.2004 PMC369507 14747535

[61] K. Min , J. Y. Kim , and S. K. Lee , “Epstein‐Barr Virus miR‐BART1‐3p Suppresses Apoptosis and Promotes Migration of Gastric Carcinoma Cells by Targeting DAB2,” International Journal of Biological Sciences 16, no. 4 (2020): 694–707. 10.7150/ijbs.36595 PMC6990914 32025216

[62] A. Shinozaki‐Ushiku , A. Kunita , and M. Isogai , “Profiling of Virus‐Encoded MicroRNAs in Epstein‐Barr Virus‐Associated Gastric Carcinoma and Their Roles in Gastric Carcinogenesis,” Journal of Virology 89, no. 10 (2015): 5581–5591. 10.1128/JVI.03639-14 PMC4442544 25740983

[63] C. J. Yoon , M. S. Chang , and D. H. Kim , “Epstein–Barr Virus‐Encoded miR‐BART5‐5p Upregulates PD‐L1 Through PIAS3/pSTAT3 Modulation, Worsening Clinical Outcomes of PD‐L1‐Positive Gastric Carcinomas,” Gastric Cancer 23, no. 5 (2020): 780–795. 10.1007/s10120-020-01059-3 32206940

[64] H. Kim , H. Choi , and S. K. Lee , “Epstein–Barr Virus miR‐BART20‐5p Regulates Cell Proliferation and Apoptosis by Targeting BAD,” Cancer Letters 356, no. 2 pt B (2015): 733–742. 10.1016/j.canlet.2014.10.023 25449437

[65] J. Wang , J. Ge , and Y. Wang , “EBV miRNAs BART11 and BART17‐3p Promote Immune Escape through the Enhancer‐Mediated Transcription of PD‐L1,” Nature Communications 13, no. 1 (2022): 866. 10.1038/s41467-022-28479-2 PMC8844414 35165282

[66] Y. Du , J. Y. Zhang , and L.‐P. Gong , “Hypoxia‐Induced Ebv‐circLMP2A Promotes Angiogenesis in EBV‐Associated Gastric Carcinoma Through the KHSRP/VHL/HIF1α/VEGFA Pathway,” Cancer Letters 526 (2022): 259–272. 10.1016/j.canlet.2021.11.031 34863886

[67] D. Wang , Z. Zeng , and S. Zhang , “RETRACTED: Epstein‐Barr Virus‐Encoded miR‐BART6‐3p Inhibits Cancer Cell Proliferation Through the LOC553103‐STMN1 Axis,” FASEB Journal 34, no. 6 (2020): 8012–8027. 10.1096/fj.202000039RR 32306460

[68] M. Bauer , Z. Nascakova , and A. I. Mihai , “The ALPK1/TIFA/NF‐κB Axis Links a Bacterial Carcinogen to R‐Loop‐Induced Replication Stress,” Nature Communications 11, no. 1 (2020): 5117. 10.1038/s41467-020-18857-z PMC7547021 33037203

[69] S. C. Tran , K. N. Bryant , and T. L. Cover , “The *Helicobacter pylori* Cag Pathogenicity Island as a Determinant of Gastric Cancer Risk,” Gut Microbes 16, no. 1 (2024): 2314201. 10.1080/19490976.2024.2314201 PMC10896142 38391242

[70] L. Z. Yuan , X. Shi , D. Tang , S. P. Zheng , Z. M. Xiao , and F. Wang , “Construction and Preservation of a Stable and Highly Expressed Recombinant *Helicobacter pylori* Vacuolating Cytotoxin A With Apoptotic Activity,” BMC Microbiology 21, no. 1 (2021): 229. 10.1186/s12866-021-02262-7 PMC8371779 34407768

[71] X. Fei , S. Chen , and L. Li , “ *Helicobacter pylori* Infection Promotes M1 Macrophage Polarization and Gastric Inflammation by Activation of NLRP3 Inflammasome via TNF/TNFR1 Axis,” Cell Communication and Signaling 23, no. 1 (2025): 6. 10.1186/s12964-024-02017-7 PMC11705855 39762835

[72] Y. F. Wei , X. Li , and M. R. Zhao , “ *Helicobacter pylori* Disrupts Gastric Mucosal Homeostasis by Stimulating Macrophages to Secrete CCL3,” Cell Communication and Signaling 22, no. 1 (2024): 263. 10.1186/s12964-024-01627-5 PMC11084090 38730482

[73] W. Chang , J. Bai , and S. Tian , “Autophagy Protects Gastric Mucosal Epithelial Cells From Ethanol‐Induced Oxidative Damage via mTOR Signaling Pathway,” Experimental Biology and Medicine 242, no. 10 (2017): 1025–1033. 10.1177/1535370216686221 PMC5444638 28056554

[74] C. Hernández , M. D. Barrachina , and J. Vallecillo‐Hernández , “Aspirin‐Induced Gastrointestinal Damage Is Associated With an Inhibition of Epithelial Cell Autophagy,” Journal of Gastroenterology 51, no. 7 (2016): 691–701. 10.1007/s00535-015-1137-1 26525539

[75] S.‐Y. Lu , S. Guo , and S. B. Chai , “Autophagy in Gastric Mucosa: The Dual Role and Potential Therapeutic Target,” BioMed Research International 2021 (2021): 2648065. 10.1155/2021/2648065 PMC8214476 34195260

[76] N. Yamaguchi , T. Sakaguchi , and M. Taira , “Autophagy‐Related Gene ATG7 Polymorphism Could Potentially Serve as a Biomarker of the Progression of Atrophic Gastritis,” Journal of Clinical Medicine 13, no. 2 (2024): 629. 10.3390/jcm13020629 PMC10817077 38276136

[77] Z. Zhou , C. Hu , B. Cui , et al., “Ginsenoside Rg1 Suppresses Pyroptosis via the NF‐κB/NLRP3/GSDMD Pathway to Alleviate Chronic Atrophic Gastritis In Vitro and In Vivo,” Journal of Agricultural and Food Chemistry 72, (2024): 13668–13683. 10.1021/acs.jafc.4c01271 38855973

[78] J. Sun , S. Wang , and Z. Zhao , “Oxymatrine Attenuates Ulcerative Colitis Through Inhibiting Pyroptosis Mediated by the NLRP3 Inflammasome,” Molecules 29, no. 12 (2024): 2897. 10.3390/molecules29122897 PMC11206389 38930963

[79] D. Liu , J. Peng , J. Xie , and Y. Xie , “Comprehensive Analysis of the Function of *Helicobacter*‐Associated Ferroptosis Gene YWHAE in Gastric Cancer Through Multi‐Omics Integration, Molecular Docking, and Machine Learning,” Apoptosis 29, no. 3–4 (2024): 439–456. 10.1007/s10495-023-01916-3 38001345

[80] J. C. Mills and J. R. Goldenring , “Metaplasia in the Stomach Arises From Gastric Chief Cells,” Cellular and Molecular Gastroenterology and Hepatology 4, no. 1 (2017): 85–88. 10.1016/j.jcmgh.2017.03.006 PMC5440355 28560292

[81] R. N. Jain , C. S. Brunkan , C. S. Chew , and L. C. Samuelson , “Gene Expression Profiling of Gastrin Target Genes in Parietal Cells,” Physiological Genomics 24, no. 2 (2006): 124–132. 10.1152/physiolgenomics.00133.2005 16278279

[82] Y. Murayama , J. I. Miyagawa , and S. Higashiyama , “Localization of Heparin‐Binding Epidermal Growth Factor‐Like Growth Factor in Human Gastric Mucosa,” Gastroenterology 109, no. 4 (1995): 1051–1059. 10.1016/0016-5085(95)90562-6 7557069

[83] T. Matsuhisa , T. Arakawa , and T. Watanabe , “Relation Between Bile Acid Reflux Into the Stomach and the Risk of Atrophic Gastritis and Intestinal Metaplasia: A Multicenter Study of 2283 Cases,” Digestive Endoscopy 25, no. 5 (2013): 519–525. 10.1111/den.12030 23363381

[84] M. Tatsugami , M. Ito , and S. Tanaka , “Bile Acid Promotes Intestinal Metaplasia and Gastric Carcinogenesis,” Cancer Epidemiology, Biomarkers & Prevention 21, no. 11 (2012): 2101–2107. 10.1158/1055-9965.EPI-12-0730 23010643

[85] H. Mutoh , H. Hayakawa , H. Sakamoto , M. Sashikawa , and K. Sugano , “Transgenic Cdx2 Induces Endogenous Cdx1 in Intestinal Metaplasia of Cdx2‐Transgenic Mouse Stomach,” FEBS Journal 276, no. 20 (2009): 5821–5831. 10.1111/j.1742-4658.2009.07263.x 19725873

[86] M. di Pietro , P. Lao‐Sirieix , S. Boyle , et al., “Evidence for a Functional Role of Epigenetically Regulated Midcluster HOXB Genes in the Development of Barrett Esophagus,” Proceedings of the National Academy of Sciences of the United States of America 109, no. 23 (2012): 9077–9082. 10.1073/pnas.1116933109 PMC3384195 22603795

[87] L. Raghoebir , E. R. Bakker , and J. C. Mills , “SOX2 Redirects the Developmental Fate of the Intestinal Epithelium Toward a Premature Gastric Phenotype,” Journal of Molecular Cell Biology 4, no. 6 (2012): 377–385. 10.1093/jmcb/mjs030 PMC3523556 22679103

[88] J. Bornschein , K. Tóth , and M. Selgrad , “Dysregulation of CDX1, CDX2 and SOX2 in Patients With Gastric Cancer Also Affects the Non‐Malignant Mucosa,” Journal of Clinical Pathology 66, no. 9 (2013): 819–822. 10.1136/jclinpath-2013-201448 23613102

[89] T. Yuan , Z. Ni , and C. Han , “SOX2 Interferes With the Function of CDX2 in Bile Acid‐Induced Gastric Intestinal Metaplasia,” Cancer Cell International 19 (2019): 24. 10.1186/s12935-019-0739-8 PMC6357452 30733645

[90] J. N. Freund , “Extending the Functions of the Homeotic Transcription Factor Cdx2 in the Digestive System through Nontranscriptional Activities,” World Journal of Gastroenterology 21, no. 5 (2015): 1436. 10.3748/wjg.v21.i5.1436 PMC4316086 25663763

[91] Z. Ni , Y. Min , and C. Han , “TGR5‐HNF4α Axis Contributes to Bile Acid‐Induced Gastric Intestinal Metaplasia Markers Expression,” Cell Death Discovery 6 (2020): 56. 10.1038/s41420-020-0290-3 PMC7338499 32655894

[92] N. Wang , M. Chen , and Z. Ni , “HDAC6/HNF4α Loop Mediated by miR‐1 Promotes Bile Acids‐Induced Gastric Intestinal Metaplasia,” Gastric Cancer 24, no. 1 (2021): 103–116. 10.1007/s10120-020-01108-x PMC7790803 32705446

[93] W. Jia , G. Xie , and W. Jia , “Bile Acid–Microbiota Crosstalk in Gastrointestinal Inflammation and Carcinogenesis,” Nature Reviews Gastroenterology & Hepatology 15, no. 2 (2018): 111–128. 10.1038/nrgastro.2017.119 PMC5899973 29018272

[94] H. J. Ku , “Bile Acid Increases Expression of the Histamine‐Producing Enzyme, Histidine Decarboxylase, in Gastric Cells,” World Journal of Gastroenterology 20, no. 1 (2014): 175. 10.3748/wjg.v20.i1.175 PMC3886006 24415870

[95] Y. Xu , T. Watanabe , and T. Tanigawa , “Bile Acids Induce cdx2 Expression Through the Farnesoid X Receptor in Gastric Epithelial Cells,” Journal of Clinical Biochemistry and Nutrition 46, no. 1 (2010): 81–86. 10.3164/jcbn.09-71 PMC2803137 20104269

[96] S. Li , X. Chen , L. Zhou , and B. M. Wang , “Farnesoid X Receptor Signal Is Involved in Deoxycholic Acid‐Induced Intestinal Metaplasia of Normal Human Gastric Epithelial Cells,” Oncology Reports 34, no. 5 (2015): 2674–2682. 10.3892/or.2015.4207 26324224

[97] L. Wang , Z. Gong , and X. Zhang , “Gut Microbial Bile Acid Metabolite Skews Macrophage Polarization and Contributes to High‐Fat Diet‐Induced Colonic Inflammation,” Gut Microbes 12, no. 1 (2020): 1819155. 10.1080/19490976.2020.1819155 PMC7553752 33006494

[98] C. P. Petersen , V. G. Weis , K. T. Nam , J. F. Sousa , B. Fingleton , and J. R. Goldenring , “Macrophages Promote Progression of Spasmolytic Polypeptide‐Expressing Metaplasia After Acute Loss of Parietal Cells,” Gastroenterology 146, no. 7 (2014): 1727–1738.e8. 10.1053/j.gastro.2014.02.007 PMC4035414 24534633

[99] C. P. Petersen , A. R. Meyer , and C. De Salvo , “A Signalling Cascade of IL‐33 to IL‐13 Regulates Metaplasia in the Mouse Stomach,” Gut 67, no. 5 (2018): 805–817. 10.1136/gutjnl-2016-312779 PMC5681443 28196875

[100] X. Xu , J. Cheng , and S. Luo , “Deoxycholic Acid‐Stimulated Macrophage‐Derived Exosomes Promote Spasmolytic Polypeptide‐Expressing Metaplasia in the Stomach,” Biochemical and Biophysical Research Communications 524, no. 3 (2020): 649–655. 10.1016/j.bbrc.2020.01.159 32033746

[101] J. Ma , T. Zhan , X. Zhang , W. Gao , S. Zhao , and H. Li , “Roles of Interleukins in Spasmolytic Polypeptide‑Expressing Metaplasia (Review),” International Journal of Oncology 68, no. 2 (2026): [pii]17. 10.3892/ijo.2025.5830 PMC12716909 41347815

[102] L. Zhang , X. Wang , and P. Chen , “MiR‐204 Down Regulates SIRT1 and Reverts SIRT1‐Induced Epithelial‐Mesenchymal Transition, Anoikis Resistance and Invasion in Gastric Cancer Cells,” BMC Cancer 13 (2013): 290. 10.1186/1471-2407-13-290 PMC3710153 23768087

[103] B. Xiao , J. Guo , and Y. Miao , “Detection of miR‐106a in Gastric Carcinoma and Its Clinical Significance,” Clinica Chimica Acta 400, no. 1–2 (2009): 97–102. 10.1016/j.cca.2008.10.021 18996365

[104] F. Petrocca , A. Vecchione , and C. M. Croce , “Emerging Role of miR‐106b‐25/miR‐17‐92 Clusters in the Control of Transforming Growth Factor β Signaling,” Cancer Research 68, no. 20 (2008): 8191–8194. 10.1158/0008-5472.CAN-08-1768 18922889

[105] T. Li , Y. Y. Lu , and X. D. Zhao , “MicroRNA‐296‐5p Increases Proliferation in Gastric Cancer through Repression of Caudal‐Related Homeobox 1,” Oncogene 33, no. 6 (2014): 783–793. 10.1038/onc.2012.637 23353818

[106] Z. Zhang , Z. Li , and C. Gao , “miR‐21 Plays a Pivotal Role in Gastric Cancer Pathogenesis and Progression,” Laboratory Investigation 88, no. 12 (2008): 1358–1366. 10.1038/labinvest.2008.94 18794849

[107] D. Liapopoulos , P. Sarantis , and T. Biniari , “MicroRNA Landscape in Hepatocellular Carcinoma: Metabolic Re‐Wiring, Predictive and Diagnostic Biomarkers, and Emerging Therapeutic Targets,” Biomedicines 13, no. 9 (2025): 2243. 10.3390/biomedicines13092243 PMC12467513 41007803

[108] N. Li , B. Tang , and E.‐D. Zhu , “Increased miR‐222 in *H. pylori*‐Associated Gastric Cancer Correlated With Tumor Progression by Promoting Cancer Cell Proliferation and Targeting RECK,” FEBS Letters 586, no. 6 (2012): 722–728. 10.1016/j.febslet.2012.01.025 22321642

[109] K. W. Lai , K. X. Koh , and M. Loh , “MicroRNA‐130b Regulates the Tumour Suppressor RUNX3 in Gastric Cancer,” European Journal of Cancer 46, no. 8 (2010): 1456–1463. 10.1016/j.ejca.2010.01.036 20176475

[110] M. Wang , C. Li , and B. Yu , “Overexpressed miR‐301a Promotes Cell Proliferation and Invasion by Targeting RUNX3 in Gastric Cancer,” Journal of Gastroenterology 48, no. 9 (2013): 1023–1033. 10.1007/s00535-012-0733-6 23338485

[111] P. Gu , X. Xing , and M. Tänzer , “Frequent Loss of TIMP‐3 Expression in Progression of Esophageal and Gastric Adenocarcinomas,” Neoplasia 10, no. 6 (2008): 563–572. 10.1593/neo.08208 PMC2386541 18516293

[112] L. Ma , Y. Chen , B. Zhang , and G. Liu , “Increased microRNA‐223 in *Helicobacter pylori*‐Associated Gastric Cancer Contributed to Cancer Cell Proliferation and Migration,” Bioscience, Biotechnology, and Biochemistry 78, no. 4 (2014): 602–608. 10.1080/09168451.2014.895661 25036956

[113] C. Gao , Z. Zhang , W. Liu , S. Xiao , W. Gu , and H. Lu , “Reduced microRNA‐218 Expression Is Associated With High Nuclear Factor Kappa B Activation in Gastric Cancer,” Cancer 116, no. 1 (2010): 41–49. 10.1002/cncr.24743 19890957

[114] Y. Du , Y. Xu , and L. Ding , “Down‐Regulation of miR‐141 in Gastric Cancer and Its Involvement in Cell Growth,” Journal of Gastroenterology 44, no. 6 (2009): 556–561. 10.1007/s00535-009-0037-7 19363643

[115] F. Ye , C. Tang , and W. Shi , “A MDM 2‐Dependent Positive‐Feedback Loop Is Involved in Inhibition of miR‐375 and miR‐106b Induced by *Helicobacter pylori* Lipopolysaccharide,” International Journal of Cancer 136, no. 9 (2015): 2120–2131. 10.1002/ijc.29268 25307786

[116] T. Kan , F. Sato , and T. Ito , “The miR‐106b‐25 Polycistron, Activated by Genomic Amplification, Functions as an Oncogene by Suppressing p21 and Bim,” Gastroenterology 136, no. 5 (2009): 1689–1700. 10.1053/j.gastro.2009.02.002 PMC2887605 19422085

[117] D. Kong , Y. S. Piao , and S. Yamashita , “Inflammation‐Induced Repression of Tumor Suppressor miR‐7 in Gastric Tumor Cells,” Oncogene 31, no. 35 (2012): 3949–3960. 10.1038/onc.2011.558 22139078

[118] T. Liu , H. Tang , Y. Lang , M. Liu , and X. Li , “MicroRNA‐27a Functions as an Oncogene in Gastric Adenocarcinoma by Targeting Prohibitin,” Cancer Letters 273, no. 2 (2009): 233–242. 10.1016/j.canlet.2008.08.003 18789835

[119] B. Xiao , E. D. Zhu , N. Li , et al., “Increased miR‐146a in Gastric Cancer Directly Targets SMAD4 and Is Involved in Modulating Cell Proliferation and Apoptosis,” Oncology Reports 27, no. 2 (2012): 559–566. 10.3892/or.2011.1514 22020746

[120] Z. Hou , L. Xie , L. Yu , X. Qian , and B. Liu , “MicroRNA‐146a Is Down‐Regulated in Gastric Cancer and Regulates Cell Proliferation and Apoptosis,” Medical Oncology 29, no. 2 (2012): 886–892. 10.1007/s12032-011-9862-7 21347720

[121] S. G. Crone , A. Jacobsen , and B. Federspiel , “microRNA‐146a Inhibits G Protein‐Coupled Receptor‐Mediated Activation of NF‐κB by Targeting CARD10 and COPS8 in Gastric Cancer,” Molecular Cancer 11 (2012): 71. 10.1186/1476-4598-11-71 PMC3515505 22992343

[122] Z. Hou , H. Yin , and C. Chen , “microRNA‐146a Targets the L1 Cell Adhesion Molecule and Suppresses the Metastatic Potential of Gastric Cancer,” Molecular Medicine Reports 6, no. 3 (2012): 501–506. 10.3892/mmr.2012.946 22711166

[123] T. Bou Kheir , E. Futoma‐Kazmierczak , and A. Jacobsen , “miR‐449 Inhibits Cell Proliferation and Is Down‐Regulated in Gastric Cancer,” Molecular Cancer 10 (2011): 29. 10.1186/1476-4598-10-29 PMC3070685 21418558

[124] J. Kurashige , H. Kamohara , and M. Watanabe , “MicroRNA‐200b Regulates Cell Proliferation, Invasion, and Migration by Directly Targeting ZEB2 in Gastric Carcinoma,” Annals of Surgical Oncology 19, no. suppl 3 (2012): 656–664. 10.1245/s10434-012-2217-6 22311119

[125] X. Zhao , L. Yang , and J. Hu , “Down‐Regulation of miR‐27a Might Inhibit Proliferation and Drug Resistance of Gastric Cancer Cells,” Journal of Experimental & Clinical Cancer Research 30, no. 1 (2011): 55. 10.1186/1756-9966-30-55 PMC3120716 21569481

[126] Y. Pan , F. Ren , and W. Zhang , “Regulation of BGC‐823 Cell Sensitivity to Adriamycin via miRNA‐135a‐5p,” Oncology Reports 32, no. 6 (2014): 2549–2556. 10.3892/or.2014.3546 25322930

[127] F. Wang , T. Li , and B. Zhang , “MicroRNA‐19a/b Regulates Multidrug Resistance in human Gastric Cancer Cells by Targeting PTEN,” Biochemical and Biophysical Research Communications 434, no. 3 (2013): 688–694. 10.1016/j.bbrc.2013.04.010 23603256

[128] J. Chen , C. Zhou , J. Li , et al., “miR‑21‑5p Confers Doxorubicin Resistance in Gastric Cancer Cells by Targeting PTEN and TIMP3,” International Journal of Molecular Medicine 41, no. 4 (2018): 1855–1866. 10.3892/ijmm.2018.3405 PMC5810196 29393355

[129] X. Pang , Z. Zhou , and Z. Yu , “Foxo3a‐Dependent miR‐633 Regulates Chemotherapeutic Sensitivity in Gastric Cancer by Targeting Fas‐Associated Death Domain,” RNA Biology 16, no. 2 (2019): 233–248. 10.1080/15476286.2019.1565665 PMC6380283 30628514

[130] Y. Shang , Z. Zhang , and Z. Liu , “miR‐508‐5p Regulates Multidrug Resistance of Gastric Cancer by Targeting ABCB1 and ZNRD1,” Oncogene 33, no. 25 (2014): 3267–3276. 10.1038/onc.2013.297 23893241

[131] R. Teng , Y. Hu , and J. Zhou , “Overexpression of Lin28 Decreases the Chemosensitivity of Gastric Cancer Cells to Oxaliplatin, Paclitaxel, Doxorubicin, and Fluorouracil in Part via microRNA‐107,” PLoS ONE 10, no. 12 (2015): e0143716. 10.1371/journal.pone.0143716 PMC4670127 26636340

[132] W. Zhu , H. Xu , and D. Zhu , “miR‐200bc/429 Cluster Modulates Multidrug Resistance of Human Cancer Cell Lines by Targeting BCL2 and XIAP,” Cancer Chemotherapy and Pharmacology 69, no. 3 (2012): 723–731. 10.1007/s00280-011-1752-3 21993663

[133] L. Xia , D. Zhang , and R. Du , “miR‐15b and miR‐16 Modulate Multidrug Resistance by Targeting BCL2 in Human Gastric Cancer Cells,” International Journal of Cancer 123, no. 2 (2008): 372–379. 10.1002/ijc.23501 18449891

[134] Y. Zhang , X. Qu , and C. Li , “miR‐103/107 Modulates Multidrug Resistance in Human Gastric Carcinoma by Downregulating Cav‐1,” Tumor Biology 36, no. 4 (2015): 2277–2285. 10.1007/s13277-014-2835-7 25407491

[135] Y. Fang , H. Shen , and H. Li , “miR‐106a Confers Cisplatin Resistance by Regulating PTEN/Akt Pathway in Gastric Cancer Cells,” Acta Biochimica et Biophysica Sinica 45, no. 11 (2013): 963–972. 10.1093/abbs/gmt106 24108762

[136] Y. Zhang , Q. Lu , and X. Cai , “MicroRNA‐106a Induces Multidrug Resistance in Gastric Cancer by Targeting RUNX3,” FEBS Letters 587, no. 18 (2013): 3069–3075. 10.1016/j.febslet.2013.06.058 23932924

[137] S. M. Yang , C. Huang , X. F. Li , M. Z. Yu , Y. He , and J. Li , “miR‐21 Confers Cisplatin Resistance in Gastric Cancer Cells by Regulating PTEN,” Toxicology 306 (2013): 162–168. 10.1016/j.tox.2013.02.014 23466500

[138] M. Zhu , X. Zhou , and Y. Du , “miR‐20a Induces Cisplatin Resistance of a Human Gastric Cancer Cell Line via Targeting CYLD,” Molecular Medicine Reports 14, no. 2 (2016): 1742–1750. 10.3892/mmr.2016.5413 27357419

[139] X. Zhou , W. Jin , H. Jia , J. Yan , and G. Zhang , “MiR‐223 Promotes the Cisplatin Resistance of Human Gastric Cancer Cells via Regulating Cell Cycle by Targeting FBXW7,” Journal of Experimental & Clinical Cancer Research 34, no. 1 (2015): 28. 10.1186/s13046-015-0145-6 PMC4387683 25888377

[140] Y. Zhang , W. Xu , P. Ni , A. Li , J. Zhou , and S. Xu , “MiR‐99a and MiR‐491 Regulate Cisplatin Resistance in Human Gastric Cancer Cells by Targeting CAPNS1,” International Journal of Biological Sciences 12, no. 12 (2016): 1437–1447. 10.7150/ijbs.16529 PMC5166486 27994509

[141] J. Zhou and Q. Chen , “Poor Expression of microRNA‐135b Results in the Inhibition of Cisplatin Resistance and Proliferation and Induces the Apoptosis of Gastric Cancer Cells Through MST1‐Mediated MAPK Signaling Pathway,” FASEB Journal 33, no. 3 (2019): 3420–3436. 10.1096/fj.201800618RRR PMC6629133 30576232

[142] X. Ge , X. Liu , and F. Lin , “MicroRNA‐421 Regulated by HIF‐1α Promotes Metastasis, Inhibits Apoptosis, and Induces Cisplatin Resistance by Targeting E‐Cadherin and Caspase‐3 in Gastric Cancer,” Oncotarget 7, no. 17 (2016): 24466–24482. 10.18632/oncotarget.8228 PMC5029715 27016414

[143] C. Lu , Z. Shan , C. Li , and L. Yang , “MiR‐129 Regulates Cisplatin‐Resistance in Human Gastric Cancer Cells by Targeting P‐gp,” Biomedicine & Pharmacotherapy 86 (2017): 450–456. 10.1016/j.biopha.2016.11.139 28012924

[144] Q. Wu , Z. Yang , and L. Xia , “Methylation of miR‐129‐5p CpG Island Modulates Multi‐Drug Resistance in Gastric Cancer by Targeting ABC Transporters,” Oncotarget 5, no. 22 (2014): 11552–11563. 10.18632/oncotarget.2594 PMC4294356 25344911

[145] Y. Shang , B. Feng , and L. Zhou , “The miR27b‐CCNG1‐P53‐miR‐508‐5p Axis Regulates Multidrug Resistance of Gastric Cancer,” Oncotarget 7, no. 1 (2016): 538–549. 10.18632/oncotarget.6374 PMC4808016 26623719

[146] W. Zhu , X. Shan , T. Wang , Y. Shu , and P. Liu , “miR‐181b Modulates Multidrug Resistance by Targeting BCL2 in Human Cancer Cell Lines,” International Journal of Cancer 127, no. 11 (2010): 2520–2529. 10.1002/ijc.25260 20162574

[147] W. Zhu , D. Zhu , and S. Lu , “miR‐497 Modulates Multidrug Resistance of Human Cancer Cell Lines by Targeting BCL2,” Medical Oncology 29, no. 1 (2012): 384–391. 10.1007/s12032-010-9797-4 21258880

[148] J. Hu , Y. Fang , Y. Cao , R. Qin , and Q. Chen , “miR‐449a Regulates Proliferation and Chemosensitivity to Cisplatin by Targeting Cyclin D1 and BCL2 in SGC7901 Cells,” Digestive Diseases and Sciences 59, no. 2 (2014): 336–345. 10.1007/s10620-013-2923-3 24248414

[149] T. Wang , G. Ge , and Y. Ding , “MiR‐503 Regulates Cisplatin Resistance of Human Gastric Cancer Cell Lines by Targeting IGF1R and BCL2,” Chinese Medical Journal 127, no. 12 (2014): 2357–2362. 24931256

[150] M. Yang , X. Shan , and X. Zhou , “miR‐1271 Regulates Cisplatin Resistance of Human Gastric Cancer Cell Lines by Targeting IGF1R, IRS1, mTOR, and BCL2,” Anti‐Cancer Agents in Medicinal Chemistry 14, no. 6 (2014): 884–891. 10.2174/1871520614666140528161318 24875127

[151] M. Zhuang , Q. Shi , and X. Zhang , “Involvement of miR‐143 in Cisplatin Resistance of Gastric Cancer Cells via Targeting IGF1R and BCL2,” Tumor Biology 36, no. 4 (2015): 2737–2745. 10.1007/s13277-014-2898-5 25492481

[152] X. Du , B. Liu , X. Luan , Q. Cui , and L. Li , “miR‐30 Decreases Multidrug Resistance in Human Gastric Cancer Cells by Modulating Cell Autophagy,” Experimental and Therapeutic Medicine 15, no. 1 (2018): 599–605. 10.3892/etm.2017.5354 PMC5763658 29375703

[153] B. Li , W. Wang , and Z. Li , “MicroRNA‐148a‐3p Enhances Cisplatin Cytotoxicity in Gastric Cancer Through Mitochondrial Fission Induction and Cyto‐Protective Autophagy Suppression,” Cancer Letters 410 (2017): 212–227. 10.1016/j.canlet.2017.09.035 PMC5675767 28965855

[154] J. Zhao , Y. Nie , H. Wang , and Y. Lin , “MiR‐181a Suppresses Autophagy and Sensitizes Gastric Cancer Cells to Cisplatin,” Gene 576, no. 2 pt 2 (2016): 828–833. 10.1016/j.gene.2015.11.013 26589846

[155] X. Xie , N. Huang , and Y. Zhang , “MiR‐192‐5p Reverses Cisplatin Resistance by Targeting ERCC3 and ERCC4 in SGC7901/DDP Cells,” Journal of Cancer 10, no. 4 (2019): 1039–1051. 10.7150/jca.25814 PMC6400793 30854110

[156] J. Ning , Y. Jiao , X. Xie , et al., “miR‑138‑5p Modulates the Expression of Excision Repair Cross‑Complementing Proteins ERCC1 and ERCC4, and Regulates the Sensitivity of Gastric Cancer Cells to Cisplatin,” Oncology Reports 41, no. 2 (2019): 1131–1139. 10.3892/or.2018.6907 30535472

[157] X. Li , J. Liang , and Y. X. Liu , “miR‐149 Reverses Cisplatin Resistance of Gastric Cancer SGC7901/DDP Cells by Targeting FoxM1,” Die Pharmazie 71, no. 11 (2016): 640–643. 10.1691/ph.2016.6696 29441968

[158] Z. Zhang , Y. Kong , and W. Yang , “Upregulation of microRNA‐34a Enhances the DDP Sensitivity of Gastric Cancer Cells by Modulating Proliferation and Apoptosis via Targeting MET,” Oncology Reports 36, no. 4 (2016): 2391–2397. 10.3892/or.2016.5016 27513895

[159] J. Wang , X. Xue , and H. Hong , “Upregulation of microRNA‐524‐5p Enhances the Cisplatin Sensitivity of Gastric Cancer Cells by Modulating Proliferation and Metastasis via Targeting SOX9,” Oncotarget 8, no. 1 (2017): 574–582. 10.18632/oncotarget.13479 PMC5352179 27880941

[160] L. H. Yan , Z. N. Chen , and J. Chen , “miR‐135a Promotes Gastric Cancer Progression and Resistance to Oxaliplatin,” Oncotarget 7, no. 43 (2016): 70699–70714. 10.18632/oncotarget.12208 PMC5342584 27683111

[161] D. M. Wu , X.‐W. Hong , and L. L. Wang , “MicroRNA‐17 Inhibition Overcomes Chemoresistance and Suppresses Epithelial‐Mesenchymal Transition Through a DEDD‐Dependent Mechanism in Gastric Cancer,” International Journal of Biochemistry & Cell Biology 102 (2018): 59–70. 10.1016/j.biocel.2018.06.007 29953965

[162] B. Jian , Z. Li , D. Xiao , G. He , L. Bai , and Q. Yang , “Downregulation of microRNA‐193‐3p Inhibits Tumor Proliferation Migration and Chemoresistance in Human Gastric Cancer by Regulating PTEN Gene,” Tumor Biology 37, no. 7 (2016): 8941–8949. 10.1007/s13277-015-4727-x 26753960

[163] P. Zhu , J. Zhang , J. Zhu , J. Shi , Q. Zhu , and Y. Gao , “MiR‐429 Induces Gastric Carcinoma Cell Apoptosis Through Bcl‐2,” Cellular Physiology and Biochemistry 37, no. 4 (2015): 1572–1580. 10.1159/000438524 26513239

[164] A. Korourian , R. Roudi , A. Shariftabrizi , and Z. Madjd , “MicroRNA‐31 Inhibits RhoA‐Mediated Tumor Invasion and Chemotherapy Resistance in MKN‐45 Gastric Adenocarcinoma Cells,” Experimental Biology and Medicine 242, no. 18 (2017): 1842–1847. 10.1177/1535370217728460 PMC5714140 28836853

[165] A. Korourian , Z. Madjd , R. Roudi , A. Shariftabrizi , and M. Soleimani , “Induction of miR‐31 Causes Increased Sensitivity to 5‐FU and Decreased Migration and Cell Invasion in Gastric Adenocarcinoma,” Bratislavské lekárske listy 120, no. 1 (2019): 35–39. 10.4149/BLL_2019_005 30685990

[166] K.‐K. Sun , X. J. Shen , and D. Yang , “MicroRNA‐31 Triggers G2/M Cell Cycle Arrest, Enhances the Chemosensitivity and Inhibits Migration and Invasion of Human Gastric Cancer Cells by Downregulating the Expression of Zeste Homolog 2 (ZH2),” Archives of Biochemistry and Biophysics 663 (2019): 269–275. 10.1016/j.abb.2019.01.023 30677405

[167] J. M. Noto , M. B. Piazuelo , and R. Chaturvedi , “Strain‐Specific Suppression of microRNA‐320 by Carcinogenic *Helicobacter pylori* Promotes Expression of the Antiapoptotic Protein Mcl‐1,” American Journal of Physiology‐Gastrointestinal and Liver Physiology 305, no. 11 (2013): G786–G796. 10.1152/ajpgi.00279.2013 PMC3882435 24136787

[168] X. Zhou , Y. Xia , L. Li , and G. Zhang , “MiR‐101 Inhibits Cell Growth and Tumorigenesis of *Helicobacter pylori* Related Gastric Cancer by Repression of SOCS2,” Cancer Biology & Therapy 16, no. 1 (2015): 160–169. 10.4161/15384047.2014.987523 PMC4622743 25561270

[169] Y. Feng , L. Wang , and J. Zeng , “FoxM1 Is Overexpressed in *Helicobacter pylori*‐Induced Gastric Carcinogenesis and Is Negatively Regulated by miR‐370,” Molecular Cancer Research 11, no. 8 (2013): 834–844. 10.1158/1541-7786.MCR-13-0007 23576572

[170] J. Shen , Z. Xiao , and W. K. K. Wu , “Epigenetic Silencing of miR‐490‐3p Reactivates the Chromatin Remodeler SMARCD1 to Promote *Helicobacter pylori*‐Induced Gastric Carcinogenesis,” Cancer Research 75, no. 4 (2015): 754–765. 10.1158/0008-5472.CAN-14-1301 25503559

[171] Y. Zhu , Q. Jiang , and X. Lou , “MicroRNAs Up‐Regulated by CagA of *Helicobacter pylori* Induce Intestinal Metaplasia of Gastric Epithelial Cells,” PLoS ONE 7, no. 4 (2012): e35147. 10.1371/journal.pone.0035147 PMC3335061 22536353

[172] K. Binnemars‐Postma , G. Storm , and J. Prakash , “Nanomedicine Strategies to Target Tumor‐Associated Macrophages,” International Journal of Molecular Sciences 18, no. 5 (2017): 979. 10.3390/ijms18050979 PMC5454892 28471401

[173] A. Shapouri‐Moghaddam , S. Mohammadian , and H. Vazini , “Macrophage Plasticity, Polarization, and Function in Health and Disease,” Journal of Cellular Physiology 233, no. 9 (2018): 6425–6440. 10.1002/jcp.26429 PMC13482560 29319160

[174] S. Aziz , A. Ahmed , P. Singh , and S. Mukhopadhyay , “The Arginase‐Polyamine Signaling Axis in Immune Cells: Implications for Immune Modulation and Host‐Pathogen Interactions,” iScience 29, no. 1 (2026): 114363. 10.1016/j.isci.2025.114363 PMC12794441 41531738

[175] O. Mazuryk , I. Gurgul , and M. Oszajca , “Nitric Oxide Signaling and Sensing in Age‐Related Diseases,” Antioxidants 13, no. 10 (2024): 1213. 10.3390/antiox13101213 PMC11504650 39456466

[176] Y.‐N. Sheng , Y. H. Luo , and S. B. Liu , “Zeaxanthin Induces Apoptosis via ROS‐Regulated MAPK and AKT Signaling Pathway in Human Gastric Cancer Cells,” OncoTargets and Therapy 13 (2020): 10995–11006. 10.2147/OTT.S272514 PMC7605660 33149614

[177] J. Li , J. Sun , and Z. Zeng , “Tumour‐Associated Macrophages in Gastric Cancer: From Function and Mechanism to Application,” Clinical and Translational Medicine 13, no. 8 (2023): e1386. 10.1002/ctm2.1386 PMC10444973 37608500

[178] Y.‐L. Yang , F. Yang , and Z. Q. Huang , “T Cells, NK Cells, and Tumor‐Associated Macrophages in Cancer Immunotherapy and the Current State of the Art of Drug Delivery Systems,” Frontiers in Immunology 14 (2023): 1199173. 10.3389/fimmu.2023.1199173 PMC10348220 37457707

[179] A. Mantovani , S. Sozzani , M. Locati , P. Allavena , and A. Sica , “Macrophage Polarization: Tumor‐Associated Macrophages as a Paradigm for Polarized M2 Mononuclear Phagocytes,” Trends in Immunology 23, no. 11 (2002): 549–555. 10.1016/s1471-4906(02)02302-5 12401408

[180] P. J. Murray and T. A. Wynn , “Protective and Pathogenic Functions of Macrophage Subsets,” Nature Reviews Immunology 11, no. 11 (2011): 723–737. 10.1038/nri3073 PMC3422549 21997792

[181] L. Cassetta and J. W. Pollard , “Targeting Macrophages: Therapeutic Approaches in Cancer,” Nature Reviews Drug Discovery 17, no. 12 (2018): 887–904. 10.1038/nrd.2018.169 30361552

[182] H. Piao , L. Fu , and Y. Wang , “A Positive Feedback Loop Between Gastric Cancer Cells and Tumor‐Associated Macrophage Induces Malignancy Progression,” Journal of Experimental & Clinical Cancer Research 41, no. 1 (2022): 174. 10.1186/s13046-022-02366-6 PMC9107227 35562774

[183] D. Barkan , J. E. Green , and A. F. Chambers , “Extracellular Matrix: A Gatekeeper in the Transition From Dormancy to Metastatic Growth,” European Journal of Cancer 46, no. 7 (2010): 1181–1188. 10.1016/j.ejca.2010.02.027 PMC2856784 20304630

[184] Y. Chen , S. Zhang , Q. Wang , and X. Zhang , “Tumor‐Recruited M2 Macrophages Promote Gastric and Breast Cancer Metastasis via M2 Macrophage‐Secreted CHI3L1 Protein,” Journal of Hematology & Oncology 10, no. 1 (2017): 36. 10.1186/s13045-017-0408-0 PMC5286803 28143526

[185] K. Kessenbrock , V. Plaks , and Z. Werb , “Matrix Metalloproteinases: Regulators of the Tumor Microenvironment,” Cell 141, no. 1 (2010): 52–67. 10.1016/j.cell.2010.03.015 PMC2862057 20371345

[186] S. U. Kang , S. Y. Cho , and H. Jeong , “Matrix Metalloproteinase 11 (MMP11) in Macrophages Promotes the Migration of HER2‐Positive Breast Cancer Cells and Monocyte Recruitment Through CCL2–CCR2 Signaling,” Laboratory Investigation 102, no. 4 (2022): 376–390. 10.1038/s41374-021-00699-y 34775491

[187] C. Wei , C. Yang , and S. Wang , “Crosstalk between Cancer Cells and Tumor Associated Macrophages Is Required for Mesenchymal Circulating Tumor Cell‐Mediated Colorectal Cancer Metastasis,” Molecular Cancer 18, no. 1 (2019): 64. 10.1186/s12943-019-0976-4 PMC6441214 30927925

[188] V. Riabov , A. Gudima , N. Wang , A. Mickley , A. Orekhov , and J. Kzhyshkowska , “Role of Tumor Associated Macrophages in Tumor Angiogenesis and Lymphangiogenesis,” Frontiers in Physiology 5 (2014): 75. 10.3389/fphys.2014.00075 PMC3942647 24634660

[189] G. Bergers , R. Brekken , and G. McMahon , “Matrix Metalloproteinase‐9 Triggers the Angiogenic Switch During Carcinogenesis,” Nature Cell Biology 2, no. 10 (2000): 737–744. 10.1038/35036374 PMC2852586 11025665

[190] L. Q. Fu , W. L. Du , M. H. Cai , J. Y. Yao , Y. Y. Zhao , and X. Z. Mou , “The Roles of Tumor‐Associated Macrophages in Tumor Angiogenesis and Metastasis,” Cellular Immunology 353 (2020): 104119. 10.1016/j.cellimm.2020.104119 32446032

[191] E. Y. Lin and J. W. Pollard , “Tumor‐Associated Macrophages Press the Angiogenic Switch in Breast Cancer,” Cancer Research 67, no. 11 (2007): 5064–5066. 10.1158/0008-5472.CAN-07-0912 17545580

[192] M. A. Venneri , M. D. Palma , and M. Ponzoni , “Identification of Proangiogenic TIE2‐Expressing Monocytes (TEMs) in Human Peripheral Blood and Cancer,” Blood 109, no. 12 (2007): 5276–5285. 10.1182/blood-2006-10-053504 17327411

[193] S. Goswami , E. Sahai , and J. B. Wyckoff , “Macrophages Promote the Invasion of Breast Carcinoma Cells via a Colony‐Stimulating Factor‐1/Epidermal Growth Factor Paracrine Loop,” Cancer Research 65, no. 12 (2005): 5278–5283. 10.1158/0008-5472.CAN-04-1853 15958574

[194] A. M. Gil‐Bernabé , Š. Ferjančič , and M. Tlalka , “Recruitment of Monocytes/Macrophages by Tissue Factor‐Mediated Coagulation Is Essential for Metastatic Cell Survival and Premetastatic Niche Establishment in Mice,” Blood 119, no. 13 (2012): 3164–3175. 10.1182/blood-2011-08-376426 22327225

[195] Q. Chen , X. H. Zhang , and J. Massagué , “Macrophage Binding to Receptor VCAM‐1 Transmits Survival Signals in Breast Cancer Cells That Invade the Lungs,” Cancer Cell 20, no. 4 (2011): 538–549. 10.1016/j.ccr.2011.08.025 PMC3293160 22014578

[196] W. L. Neumann , E. Coss , M. Rugge , and R. M. Genta , “Autoimmune Atrophic Gastritis—Pathogenesis, Pathology and Management,” Nature Reviews Gastroenterology & Hepatology 10, no. 9 (2013): 529–541. 10.1038/nrgastro.2013.101 23774773

[197] L. Faass , S. C. Stein , M. Hauke , M. Gapp , M. Albanese , and C. Josenhans , “Contribution of Heptose Metabolites and the Cag Pathogenicity Island to the Activation of Monocytes/Macrophages by *Helicobacter pylori* ,” Frontiers in Immunology 12 (2021): 632154. 10.3389/fimmu.2021.632154 PMC8174060 34093525

[198] B. Z. Qian and J. W. Pollard , “Macrophage Diversity Enhances Tumor Progression and Metastasis,” Cell 141, no. 1 (2010): 39–51. 10.1016/j.cell.2010.03.014 PMC4994190 20371344

[199] M. Wu , X. Xu , and C. Yang , “Regulator of G Protein Signaling 1 Is a Potential Target in Gastric Cancer and Impacts Tumor‐Associated Macrophages,” Cancer Science 115, no. 4 (2024): 1085–1101. 10.1111/cas.16083 PMC11006993 38287908

[200] H. Wang , H. Nie , and X. Zhao , “Tumor‐Derived PRMT1 Suppresses Macrophage Antitumor Activity by Inhibiting cGAS/STING Signaling in Gastric Cancer Cells,” Cell Death & Disease 16, no. 1 (2025): 649. 10.1038/s41419-025-07960-y PMC12381180 40858547

[201] M. A. Hwang , M. Won , J. Y. Im , M. J. Kang , D. H. Kweon , and B. K. Kim , “TNF‐α Secreted From Macrophages Increases the Expression of Prometastatic Integrin αV in Gastric Cancer,” International Journal of Molecular Sciences 24, no. 1 (2022): 376. 10.3390/ijms24010376 PMC9820470 36613819

[202] Z. Li , B. Suo , and G. Long , “Exosomal miRNA‐16‐5p Derived From M1 Macrophages Enhances T Cell‐Dependent Immune Response by Regulating PD‐L1 in Gastric Cancer,” Frontiers in Cell and Developmental Biology 8 (2020): 572689. 10.3389/fcell.2020.572689 PMC7734296 33330451

[203] J. Ma , Y. Shi , Q. Lu , and D. Huang , “Inflammation‐Related Gene ADH1A Regulates the Polarization of Macrophage M1 and Influences the Malignant Progression of Gastric Cancer,” Journal of Inflammation Research 17 (2024): 4647–4665. 10.2147/JIR.S452670 PMC11264289 39045532

[204] M. Yu , J. Peng , Y. Lu , S. Li , and K. Ding , “Silencing Immune‐Infiltrating Biomarker CCDC80 Inhibits Malignant Characterization and Tumor Formation in Gastric Cancer,” BMC Cancer 24, no. 1 (2024): 724. 10.1186/s12885-024-12451-y PMC11170897 38872096

[205] Y. Zhihua , T. Yulin , and W. Yibo , “Hypoxia Decreases Macrophage Glycolysis and M1 Percentage by Targeting MicroRNA‐30c and mTOR in Human Gastric Cancer,” Cancer Science 110, no. 8 (2019): 2368–2377. 10.1111/cas.14110 PMC6676118 31222863

[206] D. M. Hardbower , M. Asim , and T. Murray‐Stewart , “Arginase 2 Deletion Leads to Enhanced M1 Macrophage Activation and Upregulated Polyamine Metabolism in Response to *Helicobacter pylori* Infection,” Amino Acids 48, no. 10 (2016): 2375–2388. 10.1007/s00726-016-2231-2 PMC5042810 27074721

[207] V. Gambardella , J. Castillo , and N. Tarazona , “The Role of Tumor‐Associated Macrophages in Gastric Cancer Development and Their Potential as a Therapeutic Target,” Cancer Treatment Reviews 86 (2020): 102015. 10.1016/j.ctrv.2020.102015 32248000

[208] I. Montalbán del Barrio , C. Penski , and L. Schlahsa , “Adenosine‐Generating Ovarian Cancer Cells Attract Myeloid Cells Which Differentiate Into Adenosine‐Generating Tumor Associated Macrophages – A Self‐Amplifying, CD39‐ and CD73‐Dependent Mechanism for Tumor Immune Escape,” Journal for ImmunoTherapy of Cancer 4 (2016): 49. 10.1186/s40425-016-0154-9 PMC4986205 27532024

[209] O. R. Colegio , N. Q. Chu , and A. L. Szabo , “Functional Polarization of Tumour‐Associated Macrophages by Tumour‐Derived Lactic Acid,” Nature 513, no. 7519 (2014): 559–563. 10.1038/nature13490 PMC4301845 25043024

[210] P. Chen , H. Zuo , and H. Xiong , “Gpr132 Sensing of Lactate Mediates Tumor–Macrophage Interplay to Promote Breast Cancer Metastasis,” Proceedings of the National Academy of Sciences 114, no. 3 (2017): 580–585. 10.1073/pnas.1614035114 PMC5255630 28049847

[211] G. Zhang , Z. Gao , and X. Guo , “CAP2 Promotes Gastric Cancer Metastasis by Mediating the Interaction Between Tumor Cells and Tumor‐Associated Macrophages,” Journal of Clinical Investigation 133, no. 21 (2023): e166224. 10.1172/JCI166224 PMC10617780 37707957

[212] H. Jeong , B. Lee , and K. H. Kim , “WFDC2 Promotes Spasmolytic Polypeptide‐Expressing Metaplasia Through the Up‐Regulation of IL33 in Response to Injury,” Gastroenterology 161, no. 3 (2021): 953–967.e15, e15. 10.1053/j.gastro.2021.05.058 PMC8380710 34116028

[213] W. Li , S. L. Zhao , P. Zheng , et al., “Gastric Cancer‐Derived Mesenchymal Stem Cells Regulate the M2 Polarization of Macrophages Within Gastric Cancer Microenvironment via JAK2/STAT3 Signaling Pathway,” Zhonghua Zhong Liu Za Zhi 44, no. 7 (2022): 728–736. 10.3760/cma.j.cn112152-20200106-00008 35880339

[214] L. Zhang and S. Li , “Lactic Acid Promotes Macrophage Polarization Through MCT‐HIF1α Signaling in Gastric Cancer,” Experimental Cell Research 388, no. 2 (2020): 111846. 10.1016/j.yexcr.2020.111846 31945319

[215] L. Zheng , H. Xu , and Y. Di , “ELK4 Promotes the Development of Gastric Cancer by Inducing M2 Polarization of Macrophages Through Regulation of the KDM5A‐PJA2‐KSR1 Axis,” Journal of Translational Medicine 19, no. 1 (2021): 342. 10.1186/s12967-021-02915-1 PMC8353876 34372882

[216] P. Su , T. Yu , and Y. Zhang , “Upregulation of MELK Promotes Chemoresistance and Induces Macrophage M2 Polarization via CSF‐1/JAK2/STAT3 Pathway in Gastric Cancer,” Cancer Cell International 24, no. 1 (2024): 287. 10.1186/s12935-024-03453-8 PMC11320770 39135038

[217] Y. Wu , Y. Hao , Q. Zhuang , X. Ma , and C. Shi , “AKR1B10 Regulates M2 Macrophage Polarization to Promote the Malignant Phenotype of Gastric Cancer,” Bioscience Reports 43, no. 10 (2023): BSR20222007. 10.1042/BSR20222007 PMC10545534 37039038

[218] F. He , Y. Chen , D. He , and S. He , “USP14‐Mediated Deubiquitination of SIRT1 in Macrophage Promotes Fatty Acid Oxidation Amplification and M2 Phenotype Polarization,” Biochemical and Biophysical Research Communications 646 (2023): 19–29. 10.1016/j.bbrc.2022.12.076 36701891

[219] W. Li , X. Zhang , and F. Wu , “Gastric Cancer‐Derived Mesenchymal Stromal Cells Trigger M2 Macrophage Polarization That Promotes Metastasis and EMT in Gastric Cancer,” Cell Death & Disease 10, no. 12 (2019): 918. 10.1038/s41419-019-2131-y PMC6892854 31801938

[220] H. Luo , Y. Hao , B. Tang , D. Zeng , Y. Shi , and P. Yu , “Mouse Forestomach Carcinoma Cells Immunosuppress Macrophages Through TGF‐β1,” Turkish Journal of Gastroenterology 23, no. 6 (2012): 658–665. 10.4318/tjg.2012.0563 23794301

[221] P. Su , L. Jiang , and Y. Zhang , “Crosstalk Between Tumor‐Associated Macrophages and Tumor Cells Promotes Chemoresistance via CXCL5/PI3K/AKT/mTOR Pathway in Gastric Cancer,” Cancer Cell International 22, no. 1 (2022): 290. 10.1186/s12935-022-02717-5 PMC9508748 36151545

[222] C. Tang , X. Lei , L. Xiong , Z. Hu , and B. Tang , “HMGA1B/2 Transcriptionally Activated‐POU1F1 Facilitates Gastric Carcinoma Metastasis via CXCL12/CXCR4 Axis‐Mediated Macrophage Polarization,” Cell Death & Disease 12, no. 5 (2021): 422. 10.1038/s41419-021-03703-x PMC8084942 33927188

[223] Z. Wu , B. Qu , and M. Yuan , “CRIP1 Reshapes the Gastric Cancer Microenvironment to Facilitate Development of Lymphatic Metastasis,” Advanced Science 10, no. 26 (2023): e2303246. 10.1002/advs.202303246 PMC10502640 37409440

[224] B. Ma , J. Wang , and P. Yusufu , “Tumor‐Derived Exosome ElNF1‐AS1 Affects the Progression of Gastric Cancer by Promoting M2 Polarization of Macrophages,” Environmental Toxicology 38, no. 9 (2023): 2228–2239. 10.1002/tox.23862 37310109

[225] L. Xin , Y. Wu , and C. Liu , “Exosome‐Mediated Transfer of lncRNA HCG18 Promotes M2 Macrophage Polarization in Gastric Cancer,” Molecular Immunology 140 (2021): 196–205. 10.1016/j.molimm.2021.10.011 34735868

[226] C. Li , Z. Chen , and J. Gao , “MIR4435‐2HG in Exosomes Promotes Gastric Carcinogenesis by Inducing M2 Polarization in Macrophages,” Frontiers in Oncology 12 (2022): 1017745. 10.3389/fonc.2022.1017745 PMC9723220 36483041

[227] H. Xiao , J. Fu , R. Liu , L. Yan , Z. Zhou , and J. Yuan , “Gastric Cancer Cell‐Derived Exosomal miR‐541‐5p Induces M2 Macrophage Polarization Through DUSP3/JAK2/STAT3 Pathway,” BMC Cancer 24, no. 1 (2024): 957. 10.1186/s12885-024-12672-1 PMC11302208 39103776

[228] C. Deng , M. Huo , and H. Chu , “Exosome circATP8A1 Induces Macrophage M2 Polarization by Regulating the miR‐1‐3p/STAT6 Axis to Promote Gastric Cancer Progression,” Molecular Cancer 23, no. 1 (2024): 49. 10.1186/s12943-024-01966-4 PMC10921793 38459596

[229] S. Qiu , L. Xie , and C. Lu , “Gastric Cancer‐Derived Exosomal miR‐519a‐3p Promotes Liver Metastasis by Inducing Intrahepatic M2‐Like Macrophage‐Mediated Angiogenesis,” Journal of Experimental & Clinical Cancer Research 41, no. 1 (2022): 296. 10.1186/s13046-022-02499-8 PMC9549645 36217165

[230] Z. Ye , J. Yi , and X. Jiang , “Gastric Cancer‐Derived Exosomal let‐7g‐5p Mediated by SERPINE1 Promotes Macrophage M2 Polarization and Gastric Cancer Progression,” Journal of Experimental & Clinical Cancer Research 44, no. 1 (2025): 2. 10.1186/s13046-024-03269-4 PMC11694445 39748408

[231] C. Shu , C. Wang , and S. Chen , “ERR‐Activated GPR35 Promotes Immune Infiltration Level of Macrophages in Gastric Cancer Tissues,” Cell Death Discovery 8, no. 1 (2022): 444. 10.1038/s41420-022-01238-4 PMC9636254 36333291

[232] D. Li , L. Xia , and P. Huang , “Cancer‐Associated Fibroblast‐Secreted IGFBP7 Promotes Gastric Cancer by Enhancing Tumor Associated Macrophage Infiltration via FGF2/FGFR1/PI3K/AKT Axis,” Cell Death Discovery 9, no. 1 (2023): 17. 10.1038/s41420-023-01336-x PMC9867714 36681667

[233] T. Shi , Y. Zhang , and Y. Wang , “DKK1 Promotes Tumor Immune Evasion and Impedes Anti–PD‐1 Treatment by Inducing Immunosuppressive Macrophages in Gastric Cancer,” Cancer Immunology Research 10, no. 12 (2022): 1506–1524. 10.1158/2326-6066.CIR-22-0218 36206576

[234] T. K. Mak , K. Li , and Z. Zhao , “m6A Demethylation of NNMT in CAFs Promotes Gastric Cancer Progression by Enhancing Macrophage M2 Polarization,” Cancer Letters 611 (2024): 217422. 10.1016/j.canlet.2024.217422 39725153

[235] X. Yang , S. Cai , and Y. Shu , “Exosomal miR‐487a Derived From m2 Macrophage Promotes the Progression of Gastric Cancer,” Cell Cycle 20, no. 4 (2021): 434–444. 10.1080/15384101.2021.1878326 PMC7894454 33522393

[236] P. Zheng , Q. Luo , and W. Wang , “Tumor‐Associated Macrophages‐Derived Exosomes Promote the Migration of Gastric Cancer Cells by Transfer of Functional Apolipoprotein E,” Cell Death & Disease 9, no. 4 (2018): 434. 10.1038/s41419-018-0465-5 PMC5864742 29567987

[237] P. Zheng , L. Chen , and X. Yuan , “Exosomal Transfer of Tumor‐Associated Macrophage‐Derived miR‐21 Confers Cisplatin Resistance in Gastric Cancer Cells,” Journal of Experimental & Clinical Cancer Research 36, no. 1 (2017): 53. 10.1186/s13046-017-0528-y PMC5390430 28407783

[238] H. Y. Cui , J. S. Rong , and J. Chen , “Exosomal microRNA‐588 From M2 Polarized Macrophages Contributes to Cisplatin Resistance of Gastric Cancer Cells,” World Journal of Gastroenterology 27, no. 36 (2021): 6079–6092. 10.3748/wjg.v27.i36.6079 PMC8476330 34629821

[239] B. Qu , J. Liu , and Z. Peng , “Macrophages Enhance Cisplatin Resistance in Gastric Cancer Through the Transfer of circTEX2,” Journal of Cellular and Molecular Medicine 28, no. 5 (2024): e18070. 10.1111/jcmm.18070 PMC10902310 38102848

[240] H. Gao , J. Ma , Y. Cheng , and P. Zheng , “Exosomal Transfer of Macrophage‐Derived miR‐223 Confers Doxorubicin Resistance in Gastric Cancer,” OncoTargets and Therapy 13 (2020): 12169–12179. 10.2147/OTT.S283542 PMC7701146 33268995

[241] Z. Shen , Y. Yan , and C. Ye , “The Effect of Vasohibin‐1 Expression and Tumor‐Associated Macrophages on the Angiogenesis In Vitro and In Vivo,” Tumor Biology 37, no. 6 (2016): 7267–7276. 10.1007/s13277-015-4595-4 26666821

[242] H. Wang , B. Chen , Y. Lin , Y. Zhou , and X. Li , “Legumain Promotes Gastric Cancer Progression Through Tumor‐Associated Macrophages In Vitro and In Vivo,” International Journal of Biological Sciences 16, no. 1 (2020): 172–180. 10.7150/ijbs.36467 PMC6930372 31892854

[243] J. Guo , Y. Yan , and Y. Yan , “Tumor‐Associated Macrophages Induce the Expression of FOXQ1 to Promote Epithelial‐Mesenchymal Transition and Metastasis in Gastric Cancer Cells,” Oncology Reports 38, no. 4 (2017): 2003–2010. 10.3892/or.2017.5877 PMC5652949 28791370

[244] Z. He , D. Chen , and J. Wu , “Yes Associated Protein 1 Promotes Resistance to 5‐Fluorouracil in Gastric Cancer by Regulating GLUT3‐Dependent Glycometabolism Reprogramming of Tumor‐Associated Macrophages,” Archives of Biochemistry and Biophysics 702 (2021): 108838. 10.1016/j.abb.2021.108838 33727040

[245] J. Yin , Y. Lu , and Y. Liu , “SIGLEC11 Promotes M2 Macrophage Polarization Through AKT–mTOR Signaling and Facilitates the Progression of Gastric Cancer,” Journal for ImmunoTherapy of Cancer 13, no. 1 (2025): e010162. 10.1136/jitc-2024-010162 PMC11748936 39755581

[246] K. Meng , J. Song , and F. Qi , “The Mutualistic Relationship Between M2c Macrophages of TGFβ1 Induction and Gastric Cancer Cells: The Correlation Between Protective Mechanisms in the Tumor Microenvironment and Polarization of Subtypes of Cells,” Journal of Cancer 16, no. 5 (2025): 1598–1617. 10.7150/jca.97784 PMC11843238 39991579

[247] J. Zhang , L. Fu , and N. Yasuda‐Yoshihara , “IL‐1β Derived From Mixed‐Polarized Macrophages Activates Fibroblasts and Synergistically Forms a Cancer‐Promoting Microenvironment,” Gastric Cancer 26, no. 2 (2023): 187–202. 10.1007/s10120-022-01352-3 36513910

[248] Q. Luo , N. Zheng , and L. Jiang , “Lipid Accumulation in Macrophages Confers Protumorigenic Polarization and Immunity in Gastric Cancer,” Cancer Science 111, no. 11 (2020): 4000–4011. 10.1111/cas.14616 PMC7648032 32798273

[249] Y. Qiu , G. Lu , N. Li , Y. Hu , H. Tan , and C. Jiang , “Exosome‐Mediated Communication Between Gastric Cancer Cells and Macrophages: Implications for Tumor Microenvironment,” Frontiers in Immunology 15 (2024): 1327281. 10.3389/fimmu.2024.1327281 PMC10917936 38455041

[250] B. Ni , X. He , and Y. Zhang , “Tumor‐Associated Macrophage‐Derived GDNF Promotes Gastric Cancer Liver Metastasis via a GFRA1‐Modulated Autophagy Flux,” Cellular Oncology 46, no. 2 (2023): 315–330. 10.1007/s13402-022-00751-z PMC10060314 36808605

[251] M. Shao , J. Zhang , and J. Zhang , “SALL4 Promotes Gastric Cancer Progression via Hexokinase II Mediated Glycolysis,” Cancer Cell International 20 (2020): 188. 10.1186/s12935-020-01275-y PMC7247129 32489324

[252] J. Wang , Q. Huang , and X. Hu , “Disrupting Circadian Rhythm via the PER1–HK2 Axis Reverses Trastuzumab Resistance in Gastric Cancer,” Cancer Research 82, no. 8 (2022): 1503–1517. 10.1158/0008-5472.CAN-21-1820 PMC9662874 35255118

[253] X. H. Wang , Z. H. Jiang , H. M. Yang , Y. Zhang , and L. H. Xu , “Hypoxia‐Induced FOXO4/LDHA Axis Modulates Gastric Cancer Cell Glycolysis and Progression,” Clinical and Translational Medicine 11, no. 1 (2021): e279. 10.1002/ctm2.279 PMC7809603 33463054

[254] C. K. Huang , Y. Sun , L. Lv , and Y. Ping , “ENO1 and Cancer,” Molecular Therapy ‐ Oncolytics 24 (2022): 288–298. 10.1016/j.omto.2021.12.026 PMC8987341 35434271

[255] K. Zhang , L. Sun , and Y. Kang , “Regulation of Phosphoglycerate Kinase 1 and Its Critical Role in Cancer,” Cell Communication and Signaling 21, no. 1 (2023): 240. 10.1186/s12964-023-01256-4 PMC10506215 37723547

[256] K. Zahra , T. Dey , S. P. Mishra , and U. Pandey , “Pyruvate Kinase M2 and Cancer: The Role of PKM2 in Promoting Tumorigenesis,” Frontiers in Oncology 10 (2020): 159. 10.3389/fonc.2020.00159 PMC7061896 32195169

[257] X. Yao , Z. He , and C. Qin , “SLC2A3 Promotes Macrophage Infiltration by Glycolysis Reprogramming in Gastric Cancer,” Cancer Cell International 20 (2020): 503. 10.1186/s12935-020-01599-9 PMC7552479 33061855

[258] Y. Zhang , Z. Wu , L. Li , and M. Xie , “miR‐30a Inhibits Glioma Progression and Stem Cell‐Like Properties by Repression of Wnt5a,” Oncology Reports 40, no. 2 (2018): 1156–1162. 10.3892/or.2017.5728 28627699

[259] M. Jiang , N. Wu , and B. Xu , “Fatty Acid‐Induced CD36 Expression via O‐GlcNAcylation Drives Gastric Cancer Metastasis,” Theranostics 9, no. 18 (2019): 5359–5373. 10.7150/thno.34024 PMC6691574 31410220

[260] G. Zhao , M. Wu , X. Wang , Z. Du , and G. Zhang , “Effect of FABP5 Gene Silencing on the Proliferation, Apoptosis and Invasion of Human Gastric SGC‐7901 Cancer Cells,” Oncology Letters 14, no. 4 (2017): 4772–4778. 10.3892/ol.2017.6748 PMC5649645 29085478

[261] Y. He and X. Wang , “Identification of Molecular Features Correlating With Tumor Immunity in Gastric Cancer by Multi‐Omics Data Analysis,” Annals of Translational Medicine 8, no. 17 (2020): 1050. 10.21037/atm-20-922 PMC7575957 33145269

[262] P. He , S. Cheng , F. Hu , Z. Ma , and Y. Xia , “Up‐Regulation of DGAT1 in Cancer Tissues and Tumor‐Infiltrating Macrophages Influenced Survival of Patients With Gastric Cancer,” BMC Cancer 21, no. 1 (2021): 252. 10.1186/s12885-021-07976-5 PMC7941926 33750350

[263] S. Li , T. Wu , and Y. X. Lu , “Obesity Promotes Gastric Cancer Metastasis via Diacylglycerol Acyltransferase 2‐Dependent Lipid Droplets Accumulation and Redox Homeostasis,” Redox Biology 36 (2020): 101596. 10.1016/j.redox.2020.101596 PMC7276427 32506038

[264] W. Al‐Zoughbi , M. Pichler , and G. Gorkiewicz , “Loss of Adipose Triglyceride Lipase Is Associated With Human Cancer and Induces Mouse Pulmonary Neoplasia,” Oncotarget 7, no. 23 (2016): 33832–33840. 10.18632/oncotarget.9418 PMC5085122 27213586

[265] L. Chushi , W. Wei , X. Kangkang , F. Yongzeng , X. Ning , and C. Xiaolei , “HMGCR Is Up‐Regulated in Gastric Cancer and Promotes the Growth and Migration of the Cancer Cells,” Gene 587, no. 1 (2016): 42–47. 10.1016/j.gene.2016.04.029 27085483

[266] T. Zhu , Z. Wang , and T. Zou , “SOAT1 Promotes Gastric Cancer Lymph Node Metastasis Through Lipid Synthesis,” Frontiers in Pharmacology 12 (2021): 769647. 10.3389/fphar.2021.769647 PMC8591064 34790132

[267] T. Cao , S. Wang , and L. Qian , “NPRA Promotes Fatty Acid Metabolism and Proliferation of Gastric Cancer Cells by Binding to PPARα,” Translational Oncology 35 (2023): 101734. 10.1016/j.tranon.2023.101734 PMC10345484 37418841

[268] Y. Won , B. Jang , and S.‐H. Lee , “Oncogenic Fatty Acid Metabolism Rewires Energy Supply Chain in Gastric Carcinogenesis,” Gastroenterology 166, no. 5 (2024): 772–786.e14. 10.1053/j.gastro.2024.01.027 PMC11040571 38272100

[269] Y. Yao , X. Yang , and L. Sun , “Fatty Acid 2‐Hydroxylation Inhibits Tumor Growth and Increases Sensitivity to Cisplatin in Gastric Cancer,” EBioMedicine 41 (2019): 256–267. 10.1016/j.ebiom.2019.01.066 PMC6441949 30738828

[270] Y. Fang , R. Dou , and S. Huang , “LAMC1‐Mediated Preadipocytes Differentiation Promoted Peritoneum Pre‐Metastatic Niche Formation and Gastric Cancer Metastasis,” International Journal of Biological Sciences 18, no. 7 (2022): 3082–3101. 10.7150/ijbs.70524 PMC9066104 35541892

[271] Z. Wang , L. Y. Yip , and J. H. J. Lee , “Methionine Is a Metabolic Dependency of Tumor‐Initiating Cells,” Nature Medicine 25, no. 5 (2019): 825–837. 10.1038/s41591-019-0423-5 31061538

[272] T. Li , Y. T. Tan , and Y. X. Chen , “Methionine Deficiency Facilitates Antitumour Immunity by Altering m^6^ A Methylation of Immune Checkpoint Transcripts,” Gut 72, no. 3 (2023): 501–511. 10.1136/gutjnl-2022-326928 PMC9933173 35803704

[273] D. Li , Y. Wang , C. Dong , et al., “CST1 Inhibits Ferroptosis and Promotes Gastric Cancer Metastasis by Regulating GPX4 Protein Stability via OTUB1,” Oncogene 42, no. 2 (2023): 83–98. 10.1038/s41388-022-02537-x PMC9816059 36369321

[274] Z. Lin , J. Song , and Y. Gao , “Hypoxia‐Induced HIF‐1α/lncRNA‐PMAN Inhibits Ferroptosis by Promoting the Cytoplasmic Translocation of ELAVL1 in Peritoneal Dissemination From Gastric Cancer,” Redox Biology 52 (2022): 102312. 10.1016/j.redox.2022.102312 PMC9043498 35447413

[275] Z. Yang , S. Zou , and Y. Zhang , “ACTL6A Protects Gastric Cancer Cells Against Ferroptosis Through Induction of Glutathione Synthesis,” Nature Communications 14, no. 1 (2023): 4193. 10.1038/s41467-023-39901-8 PMC10345109 37443154

[276] W. Wang , M. Wang , and T. Du , “SHMT2 Promotes Gastric Cancer Development Through Regulation of HIF1α/VEGF/STAT3 Signaling,” International Journal of Molecular Sciences 24, no. 8 (2023): 7150. 10.3390/ijms24087150 PMC10138966 37108312

[277] B. K. Yoon , H. Kim , and T. G. Oh , “PHGDH Preserves One‐Carbon Cycle to Confer Metabolic Plasticity in Chemoresistant Gastric Cancer During Nutrient Stress,” Proceedings of the National Academy of Sciences 120, no. 21 (2023): e2217826120. 10.1073/pnas.2217826120 PMC10214193 37192160

[278] X. Feng , D. Ma , and J. Zhao , “UHMK1 Promotes Gastric Cancer Progression Through Reprogramming Nucleotide Metabolism,” EMBO Journal 39, no. 5 (2020): e102541. 10.15252/embj.2019102541 PMC7049804 31975428

[279] R. H. Mithany , M. H. Shahid , M. Manasseh , et al., “Gastric Cancer: A Comprehensive Literature Review,” Cureus 16, no. 3 (2024): e55902. 10.7759/cureus.55902 PMC11003650 38595903

[280] S. Saito , H. Yamaguchi , A. Saito , et al., “Advances in Intraperitoneal Chemotherapy for Gastric Cancer Patients With Peritoneal Metastases: Current Status of Treatment and Institutional Insights,” Journal of Clinical Medicine 14, no. 10 (2025): 3521. 10.3390/jcm14103521 PMC12112064 40429516

[281] Y. Koya , S. Hanada , and T. Murakami , “Lymphangiosis Carcinomatosa of the Liver and Extrahepatic Bile Duct due to Gastric Cancer: A Case Report and Literature Review,” Internal Medicine 64, no. 9 (2025): 1321–1326. 10.2169/internalmedicine.4357-24 PMC12120211 39293974

[282] M. Aziz , R. B. Killeen , K. Carlson , and A. Kasi , Krukenberg Tumor (StatPearls, 2026). 29489206

[283] L. Seeneevassen , E. Bessède , F. Mégraud , P. Lehours , P. Dubus , and C. Varon , “Gastric Cancer: Advances in Carcinogenesis Research and New Therapeutic Strategies,” International Journal of Molecular Sciences 22, no. 7 (2021): 3418. 10.3390/ijms22073418 PMC8037554 33810350

[284] M. Cisło , A. A. Filip , G. J. Arnold Offerhaus , et al., “Distinct Molecular Subtypes of Gastric Cancer: From Laurén to Molecular Pathology,” Oncotarget 9, no. 27 (2018): 19427–19442. 10.18632/oncotarget.24827 PMC5922408 29721214

[285] Cancer Genome Atlas Research Network , “Comprehensive Molecular Characterization of Gastric Adenocarcinoma,” Nature 513, no. 7517 (2014): 202–209. 10.1038/nature13480 PMC4170219 25079317

[286] R. Cristescu , J. Lee , and M. Nebozhyn , “Molecular Analysis of Gastric Cancer Identifies Subtypes Associated With Distinct Clinical Outcomes,” Nature Medicine 21, no. 5 (2015): 449–456. 10.1038/nm.3850 25894828

[287] S. C. Ma , Y. Zhao , T. Zhang , X. L. Ling , and D. Zhao , “Association Between the ERCC1 rs11615 Polymorphism and Clinical Outcomes of Oxaliplatin‐Based Chemotherapies in Gastrointestinal Cancer: A Meta‐Analysis,” OncoTargets and Therapy 8 (2015): 641–648. 10.2147/OTT.S80913 PMC4365759 25834456

[288] W. Wang , Q. Yang , Q. Huang , et al., “The rs2839698 Single Nucleotide Polymorphism of lncRNA H19 Is Associated With Post‐Operative Prognosis in T3 Gastric Adenocarcinoma,” Clinical Laboratory 64, no. 1 (2018): 105–112. 10.7754/Clin.Lab.2017.170706 29479897

[289] J. Liu , Q. Gu , C. Chen , and X. Liu , “The Expression and Clinical Significance of miR‐635 and miR‐519d in Gastric Cancer Patients,” Frontiers in Genetics 17 (2026): 1782820. 10.3389/fgene.2026.1782820 PMC13258530 42294502

[290] C. Yuan and W. Wang , “The Preoperative Albumin‐to‐Carcinoembryonic Antigen Ratio (ACR) Predicts Prognosis and Facilitates Risk Stratification in Gastric Cancer: A Retrospective Cohort Study,” Frontiers in Nutrition 13 (2026): 1789564. 10.3389/fnut.2026.1789564 PMC13105995 42039888

[291] Q. Liu , Y. Wei , and C. Shen , “High Expression of TMEM119 Predicts a Poor Prognosis in Gastric Cancer,” Translational Cancer Research 15, no. 3 (2026): 142. 10.21037/tcr-2025-aw-2166 PMC13066966 41969501

[292] N. Li , J. Yin , and Y. Zhao , “Exploration of m7G‐Related Immune Genes and Construction of a Prognostic Risk Model in Gastric Cancer,” Scientific Reports 16, no. 1 (2026): 17453. 10.1038/s41598-026-47456-z PMC13237151 41981105

[293] Y. C. Chen , W. L. Fang , and R. F. Wang , “Clinicopathological Variation of Lauren Classification in Gastric Cancer,” Pathology & Oncology Research 22, no. 1 (2016): 197–202. 10.1007/s12253-015-9996-6 26502923

[294] X. Yu , F. Hu , C. Li , Q. Yao , H. Zhang , and Y. Xue , “Clinicopathologic Characteristics and Prognosis of Proximal and Distal Gastric Cancer,” OncoTargets and Therapy 11 (2018): 1037–1044. 10.2147/OTT.S157378 PMC5833755 29520154

[295] S. H. Lim , Y. Choi , and N. Kim , “The Role of Serum Pepsinogen Tests for Detecting Gastric Cancer Between Sexes and Among Age Groups,” Gut and Liver 20, no. 2 (2026): 176–198. 10.5009/gnl250494 PMC12989666 41834169

[296] P. Karimi , F. Islami , S. Anandasabapathy , N. D. Freedman , and F. Kamangar , “Gastric Cancer: Descriptive Epidemiology, Risk Factors, Screening, and Prevention,” Cancer Epidemiology, Biomarkers & Prevention 23, no. 5 (2014): 700–713. 10.1158/1055-9965.EPI-13-1057 PMC4019373 24618998

[297] S. Yi , Q. Cai , and W. Dong , “Development and Validation of a Simple Prediction Risk Score for Predicting Risk of Gastric Cancer in Outpatient Department,” BMC Cancer 25, no. 1 (2025): 1898. 10.1186/s12885-025-15294-3 PMC12751690 41275148

[298] H. Yamada , S. Abe , and H. Charvat , “Precision Risk Stratification of Primary Gastric Cancer After Eradication of *H. pylori* by a DNA Methylation Marker: A Multicentre Prospective Study,” Gut 74, no. 9 (2025): 1410–1418. 10.1136/gutjnl-2025-335039 PMC12418579 40240063

[299] M. Banks and D. Graham , “Endoscopic Diagnosis of Chronic Atrophic Gastritis and Early Gastric Cancer: From Basics to Advanced Imaging,” Cancers 18, no. 11 (2026): 1846. 10.3390/cancers18111846 PMC13256978 42279428

[300] N. Nagata , T. Shimbo , J. Akiyama , et al., “Predictability of Gastric Intestinal Metaplasia by Mottled Patchy Erythema Seen on Endoscopy,” Gastroenterology Research 4, no. 5 (2011): 203–209. 10.4021/gr357w PMC5139844 27957016

[301] D. Kohoutova , M. Banks , and J. Bures , “Advances in the Aetiology & Endoscopic Detection and Management of Early Gastric Cancer,” Cancers (Basel) 13, no. 24 (2021): 6242. 10.3390/cancers13246242 PMC8699285 34944861

[302] W. Waddingham , D. G. Graham , and M. R. Banks , “Latest Advances in Endoscopic Detection of Oesophageal and Gastric Neoplasia,” Diagnostics 14, no. 3 (2024): 301. 10.3390/diagnostics14030301 PMC10855581 38337817

[303] A. Rangarajan , T. Natarajan , S. Lakshminarayanan , S. Sundersingh , J. Subramani , and G. Gopisetty , “Targeted Metabolomic Profiling of Gastric Cancer Biopsies,” Indian Journal of Medical Research 163, no. 6 (2026): 710–719. 10.25259/IJMR_3386_2025 PMC13289657 42295716

[304] M. B. Amin , F. L. Greene , and S. B. Edge , AJCC Cancer Staging Manual, 8th ed. (Springer, 2017).

[305] H. J. An , M. H. Kim , and D. H. Song , “High Albumin Expression Promotes Tumor Progression in Gastric Adenocarcinoma,” In Vivo 40, no. 3 (2026): 1485–1496. 10.21873/invivo.14299 PMC13133841 42049435

[306] E. Papaioannou , D. Ioannou , and T. Papamitsou , “Liquid Biopsies for the Diagnosis of Early‐Stage Gastric Cancer: A 5‐Year Systematic Review,” Journal of Liquid Biopsy 12 (2026): 100465. 10.1016/j.jlb.2026.100465 PMC13092028 42011166

[307] Z. Li , M. Song , S. Han , C. Jin , and J. Yang , “The Prognostic Role of Circulating Tumor Cells in Gastric Cancer: A Meta‐Analysis,” Frontiers in Oncology 12 (2022): 963091. 10.3389/fonc.2022.963091 PMC9610107 36313657

[308] M. Dinis‐Ribeiro , D. Libânio , H. Uchima , et al., “Management of Epithelial Precancerous Conditions and Early Neoplasia of the Stomach (MAPS III): European Society of Gastrointestinal Endoscopy (ESGE), European Helicobacter and Microbiota Study Group (EHMSG) and European Society of Pathology (ESP) Guideline Update 2025,” Endoscopy 57, no. 5 (2025): 504–554. 10.1055/a-2529-5025 40112834

[309] A. Facciorusso , “Endoscopic Submucosal Dissection Vs Endoscopic Mucosal Resection for Early Gastric Cancer: A Meta‐Analysis,” World Journal of Gastrointestinal Endoscopy 6, no. 11 (2014): 555. 10.4253/wjge.v6.i11.555 PMC4231495 25400870

[310] M. Monrabal Lezama , N. S. Murdoch Duncan , S. Bertona , and F. Schlottmann , “Current Standards of Lymphadenectomy in Gastric Cancer,” Updates in Surgery 75, no. 7 (2023): 1751–1758. 10.1007/s13304-023-01576-7 37358724

[311] A. Cuschieri , V. Joypaul , and P. Fayers , “Postoperative Morbidity and Mortality After D1 and D2 Resections for Gastric Cancer: Preliminary Results of the MRC Randomised Controlled Surgical Trial,” Lancet 347, no. 9007 (1996): 995–999. 10.1016/s0140-6736(96)90144-0 8606613

[312] I. Songun , H. Putter , E. M. Kranenbarg , M. Sasako , and C. J. van de Velde , “Surgical Treatment of Gastric Cancer: 15‐Year Follow‐Up Results of the Randomised Nationwide Dutch D1D2 Trial,” Lancet Oncology 11, no. 5 (2010): 439–449. 10.1016/S1470-2045(10)70070-X 20409751

[313] Y. Kawaguchi , K. Shoda , and S. Maruyama , “Short‐ and Long‐Term Outcomes After Laparoscopic Versus Open Total Gastrectomy for Advanced Gastric Cancer,” Cancer Diagnosis & Prognosis 6, no. 1 (2026): 92–105. 10.21873/cdp.10510 PMC12784174 41523584

[314] F. Zhou , Z. Ye , and C. Zheng , “Short‐ and Long‐Term Outcomes After Laparoscopic Versus Open Total Gastrectomy for Stage 0–I Gastric Cancer: A Multicenter, Retrospective Analysis,” Chinese Clinical Oncology 15, no. 2 (2026): 29. 10.21037/cco-2025-aw-152 42145013

[315] D. Cunningham , W. H. Allum , and S. P. Stenning , “Perioperative Chemotherapy Versus Surgery Alone for Resectable Gastroesophageal Cancer,” New England Journal of Medicine 355, no. 1 (2006): 11–20. 10.1056/NEJMoa055531 16822992

[316] C. Möhring , A. Mańczak , and A. Timotheou , “Perioperative Therapy With FLOT4 Significantly Increases Survival in Patients With Gastroesophageal and Gastric Cancer in a Large Real‐World Cohort,” International Journal of Cancer 153, no. 3 (2023): 609–622. 10.1002/ijc.34511 36919950

[317] Y. J. Bang , Y. W. Kim , and H. K. Yang , “Adjuvant Capecitabine and Oxaliplatin for Gastric Cancer after D2 Gastrectomy (CLASSIC): A Phase 3 Open‐Label, Randomised Controlled Trial,” Lancet 379, no. 9813 (2012): 315–321. 10.1016/S0140-6736(11)61873-4 22226517

[318] Y. Tian , P. Yang , and H. Guo , “Neoadjuvant Docetaxel, Oxaliplatin Plus Capecitabine Versus Oxaliplatin Plus Capecitabine for Patients With Locally Advanced Gastric Adenocarcinoma: Long‐Term Results of a Phase III Randomized Controlled Trial,” International Journal of Surgery 109, no. 12 (2023): 4000–4008. 10.1097/JS9.0000000000000692 PMC10720837 37678277

[319] P. Song , W. Tian , J. Zhao , Y. Liu , C. Wang , and L. Cao , “Impact of Adjuvant Chemotherapy on Survival of the Patients with pT1N + M0 Gastric Cancer: A Retrospective Cohort Study,” Journal of Investigative Surgery 39, no. 1 (2026): 2594559. 10.1080/08941939.2025.2594559 41399955

[320] F.‐Y. Wang , X. M. Huang , and Y.‐Q. Cao , “Comparison of PSOX (Paclitaxel, Oxaliplatin, S‐1) and SOX (Oxaliplatin, S‐1) as Postoperative Adjuvant Chemotherapy for Stage II‐III Gastric Cancer,” World Journal of Surgical Oncology 23, no. 1 (2025): 75. 10.1186/s12957-025-03723-3 PMC11887138 40055779

[321] Z. Jiang , Y. Xie , and W. Zhang , “Perioperative Chemotherapy With Docetaxel Plus Oxaliplatin and S‐1 (DOS) Versus Oxaliplatin Plus S‐1 (SOX) for the Treatment of Locally Advanced Gastric or Gastro‐Esophageal Junction Adenocarcinoma (MATCH): An Open‐Label, Randomized, Phase 2 Clinical Trial,” Gastric Cancer 27, no. 3 (2024): 571–579. 10.1007/s10120-024-01471-z PMC11016518 38457083

[322] O. Tanaka , A. Sugiyama , T. Omatsu , M. Tawada , C. Makita , and M. Matsuo , “Hemostatic Radiotherapy for Inoperable Gastric Cancer: A Pilot Study,” British Journal of Radiology 93, no. 1111 (2020): 20190958. 10.1259/bjr.20190958 PMC7336061 32356453

[323] J. Tey , H. Zheng , and Y. Y. Soon , “Palliative Radiotherapy in Symptomatic Locally Advanced Gastric Cancer: A Phase II Trial,” Cancer Medicine 8, no. 4 (2019): 1447–1458. 10.1002/cam4.2021 PMC6488108 30790469

[324] Y. Wada , M. Konno , and T. Tozawa , “Early Angiographic Changes After Hemostatic Radiotherapy for Gastric Cancer Bleeding, Mentioning the Mechanism and Potential Immediate Effects of the Treatment,” Gastric Cancer 28, no. 4 (2025): 712–717. 10.1007/s10120-025-01616-8 PMC12174217 40314880

[325] Y. Wang , J. Li , and G. Liu , “Advancements in Targeted Therapy for Gastric Cancer,” Therapeutic Advances in Gastroenterology 19 (2026): 17562848261439234. 10.1177/17562848261439234 PMC13087351 42004816

[326] Y. J. Bang , E. Van Cutsem , and A. Feyereislova , “Trastuzumab in Combination With Chemotherapy Versus Chemotherapy Alone for Treatment of HER2‐Positive Advanced Gastric or Gastro‐Oesophageal Junction Cancer (ToGA): A Phase 3, Open‐Label, Randomised Controlled Trial,” Lancet 376, no. 9742 (2010): 687–697. 10.1016/S0140-6736(10)61121-X 20728210

[327] S. Koch and L. Claesson‐Welsh , “Signal Transduction by Vascular Endothelial Growth Factor Receptors,” Cold Spring Harbor Perspectives in Medicine 2, no. 7 (2012): a006502. 10.1101/cshperspect.a006502 PMC3385940 22762016

[328] D. J. Webb , K. Donais , and L. A. Whitmore , “FAK–Src Signalling Through Paxillin, ERK and MLCK Regulates Adhesion Disassembly,” Nature Cell Biology 6, no. 2 (2004): 154–161. 10.1038/ncb1094 14743221

[329] I. Nakayama and D. Takahari , “The Role of Angiogenesis Targeted Therapies in Metastatic Advanced Gastric Cancer: A Narrative Review,” Journal of Clinical Medicine 12, no. 9 (2023): 3226. 10.3390/jcm12093226 PMC10178968 37176668

[330] T. O. Goetze , R.‐D. Hofheinz , and T. Gaiser , “Perioperative FLOT Plus Ramucirumab for Resectable Esophagogastric Adenocarcinoma: A Randomized Phase II/III Trial of the German AIO and Italian GOIM,” International Journal of Cancer 153, no. 1 (2023): 153–163. 10.1002/ijc.34495 36883420

[331] N. Ferrara , H. P. Gerber , and J. LeCouter , “The Biology of VEGF and Its Receptors,” Nature Medicine 9, no. 6 (2003): 669–676. 10.1038/nm0603-669 12778165

[332] X. Chen , H. Xu , and X. Chen , “First‐Line Camrelizumab (a PD‐1 Inhibitor) Plus Apatinib (an VEGFR‐2 Inhibitor) and Chemotherapy for Advanced Gastric Cancer (SPACE): A Phase 1 Study,” Signal Transduction and Targeted Therapy 9, no. 1 (2024): 73. 10.1038/s41392-024-01773-9 PMC10963362 38528050

[333] H. O. Alsaab , S. Sau , and R. Alzhrani , “PD‐1 and PD‐L1 Checkpoint Signaling Inhibition for Cancer Immunotherapy: Mechanism, Combinations, and Clinical Outcome,” Frontiers in Pharmacology 8 (2017): 561. 10.3389/fphar.2017.00561 PMC5572324 28878676

[334] M. S. Lee , J. Chao , and M. F. Mulcahy , “Phase II Study of Avelumab and Trastuzumab With FOLFOX Chemotherapy in Previously Untreated HER2‐Amplified Metastatic Gastroesophageal Adenocarcinoma,” Oncologist 30, no. 7 (2025): oyaf195. 10.1093/oncolo/oyaf195 PMC12269372 40571483

[335] A. Hosseini , T. Gharibi , F. Marofi , Z. Babaloo , and B. Baradaran , “CTLA‐4: From Mechanism to Autoimmune Therapy,” International Immunopharmacology 80 (2020): 106221. 10.1016/j.intimp.2020.106221 32007707

[336] C. Qi , J. Gong , and J. Li , “Claudin18.2‐Specific CAR T Cells in Gastrointestinal Cancers: Phase 1 Trial Interim Results,” Nature Medicine 28, no. 6 (2022): 1189–1198. 10.1038/s41591-022-01800-8 PMC9205778 35534566

[337] M. L. Squadrito , M. Etzrodt , M. De Palma , and M. J. Pittet , “MicroRNA‐Mediated Control of Macrophages and Its Implications for Cancer,” Trends in Immunology 34, no. 7 (2013): 350–359. 10.1016/j.it.2013.02.003 PMC3700601 23498847

[338] J. W. Graff , A. M. Dickson , G. Clay , A. P. McCaffrey , and M. E. Wilson , “Identifying Functional microRNAs in Macrophages With Polarized Phenotypes,” Journal of Biological Chemistry 287, no. 26 (2012): 21816–21825. 10.1074/jbc.M111.327031 PMC3381144 22549785

[339] A. A. Chaudhuri , A. Y.‐L. So , and N. Sinha , “MicroRNA‐125b Potentiates Macrophage Activation,” Journal of Immunology 187, no. 10 (2011): 5062–5068. 10.4049/jimmunol.1102001 PMC3208133 22003200

[340] S. W. Kim , K. Ramasamy , H. Bouamar , A. P. Lin , D. Jiang , and R. C. Aguiar , “MicroRNAs miR‐125a and miR‐125b Constitutively Activate the NF‐κB Pathway by Targeting the Tumor Necrosis Factor Alpha‐Induced Protein 3 (TNFAIP3, A20 ),” Proceedings of the National Academy of Sciences 109, no. 20 (2012): 7865–7870. 10.1073/pnas.1200081109 PMC3356650 22550173

[341] Y. Zhu , W. Fan , Y. Wang , H. Ding , S. Yang , and F. He , “Flavokawain B Weakens Gastric Cancer Progression via the TGF‐β1/SMAD4 Pathway and Attenuates M2 Macrophage Polarization,” Journal of Immunology Research 2022 (2022): 1–22. 10.1155/2022/4903333 PMC9308533 35879950

[342] W. Wei , Z. P. Li , Z. X. Bian , and Q. B. Han , “Astragalus Polysaccharide RAP Induces Macrophage Phenotype Polarization to M1 via the Notch Signaling Pathway,” Molecules 24, no. 10 (2019): 2016. 10.3390/molecules24102016 PMC6572696 31137782

[343] M. Sauane , R. V. Gopalkrishnan , and I. Lebedeva , “Mda‐7/IL‐24 Induces Apoptosis of Diverse Cancer Cell Lines Through JAK/STAT‐Independent Pathways,” Journal of Cellular Physiology 196, no. 2 (2003): 334–345. 10.1002/jcp.10309 12811827

[344] L. Corrales , S. M. McWhirter , T. W. Dubensky Jr , and T. F. Gajewski , “The Host STING Pathway at the Interface of Cancer and Immunity,” Journal of Clinical Investigation 126, no. 7 (2016): 2404–2411. 10.1172/JCI86892 PMC4922692 27367184

[345] L. Miao , J. Qi , and Q. Zhao , “Targeting the STING Pathway in Tumor‐Associated Macrophages Regulates Innate Immune Sensing of Gastric Cancer Cells,” Theranostics 10, no. 2 (2020): 498–515. 10.7150/thno.37745 PMC6929973 31903134

[346] G. Germano , R. Frapolli , and C. Belgiovine , “Role of Macrophage Targeting in the Antitumor Activity of Trabectedin,” Cancer Cell 23, no. 2 (2013): 249–262. 10.1016/j.ccr.2013.01.008 23410977

[347] T. Hourani , J. A. Holden , W. Li , J. C. Lenzo , S. Hadjigol , and N. M. O'Brien‐Simpson , “Tumor Associated Macrophages: Origin, Recruitment, Phenotypic Diversity, and Targeting,” Frontiers in Oncology 11 (2021): 788365. 10.3389/fonc.2021.788365 PMC8722774 34988021

[348] T. L. Rogers and I. Holen , “Tumour Macrophages as Potential Targets of Bisphosphonates,” Journal of Translational Medicine 9 (2011): 177. 10.1186/1479-5876-9-177 PMC3215187 22005011

[349] M. Cieslewicz , J. Tang , and J. L. Yu , “Targeted Delivery of Proapoptotic Peptides to Tumor‐Associated Macrophages Improves Survival,” Proceedings of the National Academy of Sciences 110, no. 40 (2013): 15919–15924. 10.1073/pnas.1312197110 PMC3791765 24046373

[350] C. Lee , S. S. Bae , H. Joo , and H. Bae , “Melittin Suppresses Tumor Progression by Regulating Tumor‐Associated Macrophages in a Lewis Lung Carcinoma Mouse Model,” Oncotarget 8, no. 33 (2017): 54951–54965. 10.18632/oncotarget.18627 PMC5589633 28903394

[351] L. Pang , Y. Pei , G. Uzunalli , H. Hyun , L. T. Lyle , and Y. Yeo , “Surface Modification of Polymeric Nanoparticles With M2pep Peptide for Drug Delivery to Tumor‐Associated Macrophages,” Pharmaceutical Research 36, no. 4 (2019): 65. 10.1007/s11095-019-2596-5 PMC6622458 30859335

[352] N. Xue , Q. Zhou , and M. Ji , “Chlorogenic Acid Inhibits Glioblastoma Growth through Repolarizating Macrophage From M2 to M1 Phenotype,” Scientific Reports 7 (2017): 39011. 10.1038/srep39011 PMC5206721 28045028

[353] L. Zhang , P. Shi , and P. Jin , “Ganodermanontriol Regulates Tumor‐Associated M2 Macrophage Polarization in Gastric Cancer,” Aging 16, no. 2 (2024): 1390–1398. 10.18632/aging.205434 PMC10866403 38244580

[354] C. Guiducci , A. P. Vicari , S. Sangaletti , G. Trinchieri , and M. P. Colombo , “Redirecting In Vivo Elicited Tumor Infiltrating Macrophages and Dendritic Cells Towards Tumor Rejection,” Cancer Research 65, no. 8 (2005): 3437–3446. 10.1158/0008-5472.CAN-04-4262 15833879

[355] H. Shime , M. Matsumoto , and H. Oshiumi , “Toll‐Like Receptor 3 Signaling Converts Tumor‐Supporting Myeloid Cells to Tumoricidal Effectors,” Proceedings of the National Academy of Sciences 109, no. 6 (2012): 2066–2071. 10.1073/pnas.1113099109 PMC3277567 22308357

[356] T. Reid , B. Oronsky , and S. Caroen , “Phase 1 Pilot Study of RRx‐001 + Nivolumab in Patients With Advanced Metastatic Cancer (PRIMETIME),” Frontiers in Immunology 14 (2023): 1104753. 10.3389/fimmu.2023.1104753 PMC10028591 36960054

[357] H. Wang , Y. Sun , and X. Zhou , “CD47/SIRPα Blocking Peptide Identification and Synergistic Effect With Irradiation for Cancer Immunotherapy,” Journal for ImmunoTherapy of Cancer 8, no. 2 (2020): e000905. 10.1136/jitc-2020-000905 PMC7537338 33020240

[358] M. N. Andersen , A. Etzerodt , and J. H. Graversen , “STAT3 Inhibition Specifically in Human Monocytes and Macrophages by CD163‐Targeted Corosolic Acid‐Containing Liposomes,” Cancer Immunology, Immunotherapy 68, no. 3 (2019): 489–502. 10.1007/s00262-019-02301-3 PMC11028169 30637473

[359] Y. Zhao , X. Lu , H. Huang , Y. Yao , H. Liu , and Y. Sun , “ *Dendrobium officinale* Polysaccharide Converts M2 Into M1 Subtype Macrophage Polarization via the STAT6/PPAR‐r and JAGGED1/NOTCH1 Signaling Pathways to Inhibit Gastric Cancer,” Molecules 28, no. 20 (2023): 7062. 10.3390/molecules28207062 PMC10609635 37894541

[360] C. B. Rodell , S. P. Arlauckas , and M. F. Cuccarese , “TLR7/8‐Agonist‐Loaded Nanoparticles Promote the Polarization of Tumour‐Associated Macrophages to Enhance Cancer Immunotherapy,” Nature Biomedical Engineering 2, no. 8 (2018): 578–588. 10.1038/s41551-018-0236-8 PMC6192054 31015631

[361] S. Mukherjee , R. Hussaini , and R. White , “TriCurin, a Synergistic Formulation of Curcumin, Resveratrol, and Epicatechin Gallate, Repolarizes Tumor‐Associated Macrophages and Triggers an Immune Response to Cause Suppression of HPV+ Tumors,” Cancer Immunology, Immunotherapy 67, no. 5 (2018): 761–774. 10.1007/s00262-018-2130-3 PMC11028238 29453519

[362] H. Jin , Y. He , and P. Zhao , “Targeting Lipid Metabolism to Overcome EMT‐Associated Drug Resistance via Integrin β3/FAK Pathway and Tumor‐Associated Macrophage Repolarization Using Legumain‐Activatable Delivery,” Theranostics 9, no. 1 (2019): 265–278. 10.7150/thno.27246 PMC6332796 30662566

[363] C. W. Shields , M. A. Evans , and L. L.‐W. Wang , “Cellular Backpacks for Macrophage Immunotherapy,” Science Advances 6, no. 18 (2020): eaaz6579. 10.1126/sciadv.aaz6579 PMC7190308 32494680

[364] S. Yang , X. Wang , and G. Contino , “Pancreatic Cancers Require Autophagy for Tumor Growth,” Genes & Development 25, no. 7 (2011): 717–729. 10.1101/gad.2016111 PMC3070934 21406549

[365] J. M. Jaynes , R. Sable , and M. Ronzetti , “Mannose Receptor (CD206) Activation in Tumor‐Associated Macrophages Enhances Adaptive and Innate Antitumor Immune Responses,” Science Translational Medicine 12, no. 530 (2020): eaax6337. 10.1126/scitranslmed.aax6337 PMC7832040 32051227

[366] D. Shae , K. W. Becker , and P. Christov , “Endosomolytic Polymersomes Increase the Activity of Cyclic Dinucleotide STING Agonists to Enhance Cancer Immunotherapy,” Nature Nanotechnology 14, no. 3 (2019): 269–278. 10.1038/s41565-018-0342-5 PMC6402974 30664751

[367] Q. Cao , X. Cheng , and R. Lv , “Nanoparticle‐Mediated CXCL12–CXCR4 Inhibition Reprograms Macrophages and Suppresses Gastric Carcinoma,” Advanced Science 12, no. 30 (2025): e00225. 10.1002/advs.202500225 PMC12376508 40536774

[368] M. Pan , F. Wang , and L. Nan , “αVEGFR2‐MICA Fusion Antibodies Enhance Immunotherapy Effect and Synergize With PD‐1 Blockade,” Cancer Immunology, Immunotherapy 72, no. 4 (2023): 969–984. 10.1007/s00262-022-03306-1 PMC10991987 36227341

[369] B. Chen , A. Gao , and B. Tu , “Metabolic Modulation via mTOR Pathway and Anti‐Angiogenesis Remodels Tumor Microenvironment Using PD‐L1‐Targeting Codelivery,” Biomaterials 255 (2020): 120187. 10.1016/j.biomaterials.2020.120187 32590192

[370] G. A. Manji , B. A. Van Tine , and S. M. Lee , “A Phase I Study of the Combination of Pexidartinib and Sirolimus to Target Tumor‐Associated Macrophages in Unresectable Sarcoma and Malignant Peripheral Nerve Sheath Tumors,” Clinical Cancer Research 27, no. 20 (2021): 5519–5527. 10.1158/1078-0432.CCR-21-1779 PMC8530953 34321280

[371] N. Cheng , X. Bai , Y. Shu , O. Ahmad , and P. Shen , “Targeting Tumor‐Associated Macrophages as an Antitumor Strategy,” Biochemical Pharmacology 183 (2021): 114354. 10.1016/j.bcp.2020.114354 33279498

[372] C. Ngambenjawong , H. H. Gustafson , J. M. Pineda , N. A. Kacherovsky , M. Cieslewicz , and S. H. Pun , “Serum Stability and Affinity Optimization of an M2 Macrophage‐Targeting Peptide (M2pep),” Theranostics 6, no. 9 (2016): 1403–1414. 10.7150/thno.15394 PMC4924508 27375788

[373] B. Kakoschky , T. Pleli , and C. Schmithals , “Selective Targeting of Tumor Associated Macrophages in Different Tumor Models,” PLoS ONE 13, no. 2 (2018): e0193015. 10.1371/journal.pone.0193015 PMC5814016 29447241

[374] A. Lepland , E. K. Asciutto , and A. Malfanti , “Targeting Pro‐Tumoral Macrophages in Early Primary and Metastatic Breast Tumors with the CD206‐Binding mUNO Peptide,” Molecular Pharmaceutics 17, no. 7 (2020): 2518–2531. 10.1021/acs.molpharmaceut.0c00226 32421341

[375] K. Li , W. Li , and S. Fu , “Organoid Models of Drug Resistant Gastric Adenosquamous Carcinoma: Recapitulating Tumor Features and Refining Precision Treatment,” Drug Resistance Updates 79 (2025): 101201. 10.1016/j.drup.2025.101201 39793300

[376] T. Liu , L. Wang , and P. Liang , “USP19 Suppresses Inflammation and Promotes M2‐Like Macrophage Polarization by Manipulating NLRP3 Function via Autophagy,” Cellular & Molecular Immunology 18, no. 10 (2021): 2431–2442. 10.1038/s41423-020-00567-7 PMC8484569 33097834

[377] Y. Pan , X. Hui , and R. L. C. Hoo , “Adipocyte‐Secreted Exosomal microRNA‐34a Inhibits M2 Macrophage Polarization to Promote Obesity‐Induced Adipose Inflammation,” Journal of Clinical Investigation 129, no. 2 (2019): 834–849. 10.1172/JCI123069 PMC6355214 30667374

[378] E. Van Overmeire , B. Stijlemans , F. Heymann , et al., “M‐CSF and GM‐CSF Receptor Signaling Differentially Regulate Monocyte Maturation and Macrophage Polarization in the Tumor Microenvironment,” Cancer Res 76, no. 1 (2016): 35–42. 10.1158/0008-5472.CAN-15-0869 26573801

[379] C. Anfray , A. Ummarino , F. T. Andón , and P. Allavena , “Current Strategies to Target Tumor‐Associated‐Macrophages to Improve Anti‐Tumor Immune Responses,” Cells 9, no. 1 (2019): 46. 10.3390/cells9010046 PMC7017001 31878087

[380] R. Wesolowski , N. Sharma , and L. Reebel , “Phase Ib Study of the Combination of Pexidartinib (PLX3397), a CSF‐1R Inhibitor, and Paclitaxel in Patients With Advanced Solid Tumors,” Therapeutic Advances in Medical Oncology 11 (2019): 1758835919854238. 10.1177/1758835919854238 PMC6589951 31258629

[381] D. Rückerl , S. J. Jenkins , N. N. Laqtom , et al., “Induction of IL‐4Rα‐Dependent MicroRNAs Identifies PI3K/Akt Signaling as Essential for IL‐4‐Driven Murine Macrophage Proliferation In Vivo,” Blood 120, no. 11 (2012): 2307–2316. 10.1182/blood-2012-02-408252 PMC3501641 22855601

[382] M. P. Boldin , K. D. Taganov , and D. S. Rao , “miR‐146a Is a Significant Brake on Autoimmunity, Myeloproliferation, and Cancer in Mice,” Journal of Experimental Medicine 208, no. 6 (2011): 1189–1201. 10.1084/jem.20101823 PMC3173243 21555486

[383] K. D. Taganov , M. P. Boldin , K. J. Chang , and D. Baltimore , “NF‐κB‐Dependent Induction of microRNA miR‐146, an Inhibitor Targeted to Signaling Proteins of Innate Immune Responses,” Proceedings of the National Academy of Sciences 103, no. 33 (2006): 12481–12486. 10.1073/pnas.0605298103 PMC1567904 16885212

[384] X. Cui , T. Qin , and Z. Zhao , “Pentraxin‐3 Inhibits Milky Spots Metastasis of Gastric Cancer by Inhibiting M2 Macrophage Polarization,” Journal of Cancer 12, no. 15 (2021): 4686–4697. 10.7150/jca.58698 PMC8210545 34149932

[385] H. Han , G. Ding , and S. Wang , “Long Non‐Coding RNA LOC339059 Attenuates IL‐6/STAT3‐Signaling‐Mediated PDL1 Expression and Macrophage M2 Polarization by Interacting With c‐Myc in Gastric Cancer,” Cancers 15, no. 22 (2023): 5313. 10.3390/cancers15225313 PMC10670112 38001573

[386] J. B. Wang , Y. X. Gao , and Y. H. Ye , “CDK5RAP3 Acts as a Tumour Suppressor in Gastric Cancer Through the Infiltration and Polarization of Tumour‐Associated Macrophages,” Cancer Gene Therapy 30, no. 1 (2023): 22–37. 10.1038/s41417-022-00515-9 PMC9842504 35999359

[387] P. Goossens , J. Rodriguez‐Vita , and A. Etzerodt , “Membrane Cholesterol Efflux Drives Tumor‐Associated Macrophage Reprogramming and Tumor Progression,” Cell Metabolism 29, no. 6 (2019): 1376–1389.e4. 10.1016/j.cmet.2019.02.016 30930171

[388] C. Wang , X. Zhu , and J. Wang , “TRIM32 Promotes Tumor Immune Evasion and Impedes Anti–PD‐1 Treatment by Inducing Immunosuppressive Macrophages in Gastric Cancer,” Journal of Translational Medicine 23, no. 1 (2025): 1187. 10.1186/s12967-025-06330-8 PMC12574041 41163195

[389] J. Xu , C. Weng , and Y. Zhang , “GPX4 Knockdown Suppresses M2 Macrophage Polarization in Gastric Cancer by Modulating Kynurenine Metabolism,” Theranostics 15, no. 12 (2025): 5826–5845. 10.7150/thno.108817 PMC12068284 40365295

[390] J. Zhang , Y. Dong , and S. Yu , “IL‐4/IL‐4R Axis Signaling Drives Resistance to Immunotherapy by Inducing the Upregulation of Fcγ Receptor IIB in M2 Macrophages,” Cell Death & Disease 15, no. 7 (2024): 500. 10.1038/s41419-024-06875-4 PMC11246528 39003253

[391] G. Zhao , L. Liu , and R. M. Peek , “Activation of Epidermal Growth Factor Receptor in Macrophages Mediates Feedback Inhibition of M2 Polarization and Gastrointestinal Tumor Cell Growth,” Journal of Biological Chemistry 291, no. 39 (2016): 20462–20472. 10.1074/jbc.M116.750182 PMC5034042 27507810

[392] A. Kuratani , M. Okamoto , and K. Kishida , “Platelet Factor 4–Induced T_H_1‐T_reg_ Polarization Suppresses Antitumor Immunity,” Science 386, no. 6724 (2024): eadn8608. 10.1126/science.adn8608 39571033

[393] A. Mantovani , F. Marchesi , A. Malesci , L. Laghi , and P. Allavena , “Tumour‐Associated Macrophages as Treatment Targets in Oncology,” Nature Reviews Clinical Oncology 14, no. 7 (2017): 399–416. 10.1038/nrclinonc.2016.217 PMC5480600 28117416

[394] A. Vidyarthi , N. Khan , and T. Agnihotri , “TLR‐3 Stimulation Skews M2 Macrophages to M1 Through IFN‐αβ Signaling and Restricts Tumor Progression,” Frontiers in Immunology 9 (2018): 1650. 10.3389/fimmu.2018.01650 PMC6060442 30072995

[395] Y. Xia , L. Rao , H. Yao , Z. Wang , P. Ning , and X. Chen , “Engineering Macrophages for Cancer Immunotherapy and Drug Delivery,” Advanced Materials 32, no. 40 (2020): e2002054. 10.1002/adma.202002054 32856350

[396] S. B. Willingham , J. P. Volkmer , and A. J. Gentles , “The CD47‐Signal Regulatory Protein Alpha (SIRPa) Interaction Is a Therapeutic Target for Human Solid Tumors,” Proceedings of the National Academy of Sciences 109, no. 17 (2012): 6662–6667. 10.1073/pnas.1121623109 PMC3340046 22451913

[397] H. D. Lum , I. N. Buhtoiarov , and B. E. Schmidt , “In Vivo CD40 Ligation Can Induce T Cell‐Independent Antitumor Effects That Involve Macrophages,” Journal of Leukocyte Biology 79, no. 6 (2006): 1181–1192. 10.1189/jlb.0405191 16565324

[398] C. Fu , L. Jiang , and S. Hao , “Activation of the IL‐4/STAT6 Signaling Pathway Promotes Lung Cancer Progression by Increasing M2 Myeloid Cells,” Frontiers in Immunology 10 (2019): 2638. 10.3389/fimmu.2019.02638 PMC6863933 31798581

[399] J. Wu , X. X. Zhang , and X. Zou , “The Effect of Jianpi Yangzheng Xiaozheng Decoction and Its Components on Gastric Cancer,” Journal of Ethnopharmacology 235 (2019): 56–64. 10.1016/j.jep.2019.02.003 30731181

[400] Y. Chen , J. Liu , and Y. Chen , “Jianpi Yangzheng Xiaozheng Decoction Alleviates Gastric Cancer Progression via Suppressing Exosomal PD‐L1,” Frontiers in Pharmacology 14 (2023): 1159829. 10.3389/fphar.2023.1159829 PMC10434994 37601051

[401] X. Wang , J. Li , and R. Qin , “Jianpi Yangzheng Xiaozheng Granule Induced Ferroptosis to Suppress Gastric Cancer Progression Through Reprogramming Lipid Metabolism via SCD1/Wnt/β‐Catenin Axis,” Frontiers in Molecular Biosciences 12 (2025): 1523494. 10.3389/fmolb.2025.1523494 PMC11893430 40070686

[402] A. Farooque , F. Afrin , J. S. Adhikari , and B. S. Dwarakanath , “Polarization of Macrophages Towards M1 Phenotype by a Combination of 2‐Deoxy‐D‐Glucose and Radiation: Implications for Tumor Therapy,” Immunobiology 221, no. 2 (2016): 269–281. 10.1016/j.imbio.2015.10.009 26597503

[403] F. Wang , S. Zhang , and I. Vuckovic , “Glycolytic Stimulation Is Not a Requirement for M2 Macrophage Differentiation,” Cell Metabolism 28, no. 3 (2018): 463–475.e4. 10.1016/j.cmet.2018.08.012 PMC6449248 30184486

[404] Y. X. Lin , Y. Wang , and S. Blake , “RNA Nanotechnology‐Mediated Cancer Immunotherapy,” Theranostics 10, no. 1 (2020): 281–299. 10.7150/thno.35568 PMC6929632 31903120

[405] L. Zhang , L. Tian , and X. Dai , “Pluripotent Stem Cell‐Derived CAR‐Macrophage Cells With Antigen‐Dependent Anti‐Cancer Cell Functions,” Journal of Hematology & Oncology 13, no. 1 (2020): 153. 10.1186/s13045-020-00983-2 PMC7656711 33176869

[406] M. Klichinsky , M. Ruella , and O. Shestova , “Human Chimeric Antigen Receptor Macrophages for Cancer Immunotherapy,” Nature Biotechnology 38, no. 8 (2020): 947–953. 10.1038/s41587-020-0462-y PMC7883632 32361713

[407] R. Noy and J. W. Pollard , “Tumor‐Associated Macrophages: From Mechanisms to Therapy,” Immunity 41, no. 1 (2014): 49–61. 10.1016/j.immuni.2014.06.010 PMC4137410 25035953

[408] S. Wang , Y. Yang , and P. Ma , “CAR‐Macrophage: An Extensive Immune Enhancer to Fight Cancer,” EBioMedicine 76 (2022): 103873. 10.1016/j.ebiom.2022.103873 PMC8844597 35152151

[409] S. Zinn , R. Vazquez‐Lombardi , C. Zimmermann , P. Sapra , L. Jermutus , and D. Christ , “Advances in Antibody‐Based Therapy in Oncology,” Nature Cancer 4 no. 2 (2023): 165–180. 10.1038/s43018-023-00516-z 36806801

[410] Y. Liu , L. Xiao , and M. Yang , “CAR‐Armored‐Cell Therapy in Solid Tumor Treatment,” Journal of Translational Medicine 22, no. 1 (2024): 1076. 10.1186/s12967-024-05903-3 PMC11603843 39609705

[411] Y. Shi , X. Li , Y. Dong , H. Yuan , Y. Wang , and R. Yang , “Exploring the Potential of CAR‐Macrophage Therapy,” Life Sciences 361 (2025): 123300. 10.1016/j.lfs.2024.123300 39643037

[412] Q. Zeng and C. M. Jewell , “Directing Toll‐Like Receptor Signaling in Macrophages to Enhance Tumor Immunotherapy,” Current Opinion in Biotechnology 60 (2019): 138–145. 10.1016/j.copbio.2019.01.010 PMC6717700 30831487

[413] B. Sarkar , S. P. Arlauckas , M. F. Cuccarese , C. S. Garris , R. Weissleder , and C. B. Rodell , “Host‐Functionalization of Macrin Nanoparticles to Enable Drug Loading and Control Tumor‐Associated Macrophage Phenotype,” Frontiers in Immunology 15 (2024): 1331480. 10.3389/fimmu.2024.1331480 PMC10965546 38545103

[414] P. Figueiredo , A. Lepland , and P. Scodeller , “Peptide‐Guided Resiquimod‐Loaded Lignin Nanoparticles Convert Tumor‐Associated Macrophages From M2 to M1 Phenotype for Enhanced Chemotherapy,” Acta Biomaterialia 133 (2021): 231–243. 10.1016/j.actbio.2020.09.038 33011297

[415] R. Goswami , A. Nabawy , and M. Jiang , “All‐Natural Gelatin‐Based Nanoemulsion Loaded With TLR 7/8 Agonist for Efficient Modulation of Macrophage Polarization for Immunotherapy,” Nanomaterials 14, no. 19 (2024): 1556. 10.3390/nano14191556 PMC11477480 39404283

[416] H. Y. Kim , R. Li , and T. S. C. Ng , “Quantitative Imaging of Tumor‐Associated Macrophages and Their Response to Therapy Using^64^ Cu‐Labeled Macrin,” ACS Nano 12, no. 12 (2018): 12015–12029. 10.1021/acsnano.8b04338 PMC6482841 30508377

[417] E. J. Keliher , J. Yoo , and M. Nahrendorf , “ ^89^Zr‐Labeled Dextran Nanoparticles Allow In Vivo Macrophage Imaging,” Bioconjugate Chemistry 22, no. 12 (2011): 2383–2389. 10.1021/bc200405d PMC3244512 22035047

[418] E. J. Keliher , Y.‐X. Ye , and G. R. Wojtkiewicz , “Polyglucose Nanoparticles With Renal Elimination and Macrophage Avidity Facilitate PET Imaging in Ischaemic Heart Disease,” Nature Communications 8 (2017): 14064. 10.1038/ncomms14064 PMC5241815 28091604

[419] V. Turco , K. Pfleiderer , and J. Hunger , “T Cell‐Independent Eradication of Experimental Glioma by Intravenous TLR7/8‐Agonist‐Loaded Nanoparticles,” Nature Communications 14, no. 1 (2023): 771. 10.1038/s41467-023-36321-6 PMC9922247 36774352

[420] C. B. Rodell , M. S. Ahmed , C. S. Garris , M. J. Pittet , and R. Weissleder , “Development of Adamantane‐Conjugated TLR7/8 Agonists for Supramolecular Delivery and Cancer Immunotherapy,” Theranostics 9, no. 26 (2019): 8426–8436. 10.7150/thno.35434 PMC6924259 31879528

[421] C. Anfray , F. Mainini , and E. Digifico , “Intratumoral Combination Therapy With Poly(I:C) and Resiquimod Synergistically Triggers Tumor‐Associated Macrophages for Effective Systemic Antitumoral Immunity,” Journal for ImmunoTherapy of Cancer 9, no. 9 (2021): e002408. 10.1136/jitc-2021-002408 PMC8449972 34531246

[422] Y. Guo , Y. Feng , X. Cui , Q. Wang , and X. Pan , “Autophagy Inhibition Induces the Repolarisation of Tumour‐Associated Macrophages and Enhances Chemosensitivity of Laryngeal Cancer Cells to Cisplatin in Mice,” Cancer Immunology, Immunotherapy 68, no. 12 (2019): 1909–1920. 10.1007/s00262-019-02415-8 PMC11028258 31641796

[423] M. G. Scott , E. Dullaghan , and N. Mookherjee , “An Anti‐Infective Peptide That Selectively Modulates the Innate Immune Response,” Nature Biotechnology 25, no. 4 (2007): 465–472. 10.1038/nbt1288 17384586

[424] Q. Xing , Y. Feng , and H. Sun , “Scavenger Receptor MARCO Contributes to Macrophage Phagocytosis and Clearance of Tumor Cells,” Experimental Cell Research 408, no. 2 (2021): 112862. 10.1016/j.yexcr.2021.112862 34626585

[425] J. Cao and C. Liu , “Mechanistic Studies of Tumor‐Associated Macrophage Immunotherapy,” Frontiers in Immunology 15 (2024): 1476565. 10.3389/fimmu.2024.1476565 PMC11472702 39403370

[426] Q. Wang , J. S. Bergholz , and L. Ding , “STING Agonism Reprograms Tumor‐Associated Macrophages and Overcomes Resistance to PARP Inhibition in BRCA1‐Deficient Models of Breast Cancer,” Nature Communications 13, no. 1 (2022): 3022. 10.1038/s41467-022-30568-1 PMC9156717 35641483

[427] L. Quero , A. N. Tiaden , and E. Hanser , “miR‐221‐3p Drives the Shift of M2‐Macrophages to a Pro‐Inflammatory Function by Suppressing JAK3/STAT3 Activation,” Frontiers in Immunology 10 (2019): 3087. 10.3389/fimmu.2019.03087 PMC6996464 32047494

[428] X. Wang , X. Jiao , and Y. Meng , “Methionine Enkephalin (MENK) Inhibits Human Gastric Cancer Through Regulating Tumor Associated Macrophages (TAMs) and PI3K/AKT/mTOR Signaling Pathway Inside Cancer Cells,” International Immunopharmacology 65 (2018): 312–322. 10.1016/j.intimp.2018.10.023 30343258

[429] E. M. Palmieri , A. Menga , and R. Martín‐Pérez , “Pharmacologic or Genetic Targeting of Glutamine Synthetase Skews Macrophages Toward an M1‐Like Phenotype and Inhibits Tumor Metastasis,” Cell Reports 20, no. 7 (2017): 1654–1666. 10.1016/j.celrep.2017.07.054 PMC5575233 28813676

[430] J. Park , S. E. Lee , and J. Hur , “M‐CSF From Cancer Cells Induces Fatty Acid Synthase and PPARβ/δ Activation in Tumor Myeloid Cells, Leading to Tumor Progression,” Cell Reports 10, no. 9 (2015): 1614–1625. 10.1016/j.celrep.2015.02.024 25753425

[431] H. Chen , Q. Ye , and J. Lv , “Evaluation of Trastuzumab Anti‐Tumor Efficacy and Its Correlation With HER‐2 Status in Patient‐Derived Gastric Adenocarcinoma Xenograft Models,” Pathology & Oncology Research 21, no. 4 (2015): 947–955. 10.1007/s12253-015-9909-8 PMC4550655 25749810

[432] T. Satoh , P. Barthélémy , and L. Nogova , “Phase 2 Study of Futibatinib in Patients With Gastric or Gastroesophageal Junction Cancer Harboring FGFR2 Amplifications,” European Journal of Cancer 218 (2025): 115262. 10.1016/j.ejca.2025.115262 39919334

[433] M. A. Shah , K. Shitara , and J. A. Ajani , “Zolbetuximab Plus CAPOX in CLDN18.2‐Positive Gastric or Gastroesophageal Junction Adenocarcinoma: The Randomized, Phase 3 GLOW Trial,” Nature Medicine 29, no. 8 (2023): 2133–2141. 10.1038/s41591-023-02465-7 PMC10427418 37524953

[434] J. Yuan , J. Gong , and Z. Peng , “Efficacy and Safety of Infigratinib in Patients With Refractory Advanced Gastric or Gastroesophageal Junction Adenocarcinoma Harboring FGFR2 Gene Amplification: A Single‐Arm, Multicenter Phase 2 Trial,” British Journal of Cancer 135, no. 6 (2026): 924–930. 10.1038/s41416-026-03511-0 PMC13534696 42337043

[435] G. Yeerkenbieke , X. Duan , and Y. Feng , “Dual Targeting of Chemoresistance and Ferroptosis in Gastric Cancer: DDR1/VEGFR2 Inhibitor K‐13 Synergizes With Docetaxel From Preclinical Models to Phase Ib/II Clinical Trial,” Cell Death & Differentiation (2026). 10.1038/s41418-026-01751-4 42128929

[436] J. Raimbourg , M. B. Abdelghani , and V. Boige , “Antiangiogenic Therapies in Gastric Cancer: Future Directions for 2026 and Beyond: An UNICANCER Gastrointestinal Group (UCGI) Perspective,” Cancer Treatment Reviews 148 (2026): 103177. 10.1016/j.ctrv.2026.103177 42385352

[437] J. H. Strickler , J. Raimbourg , and J. E. Cohen , “Telisotuzumab Adizutecan (ABBV‐400), a Novel c‐Met–Targeting Antibody–Drug Conjugate: First‐in‐Human Results in Advanced Gastric/Gastroesophageal Junction Cancer,” Clinical Cancer Research 32, no. 12 (2026): 2352–2361. 10.1158/1078-0432.CCR-25-3597 PMC13266338 41910595

[438] A. Gordon , C. Fong , and L. Satchwell , “Maintenance Rucaparib After First‐Line Platinum‐Based Chemotherapy in HER2‐Negative Advanced Oesophagogastric Adenocarcinoma: Results From the PLATFORM Study,” ESMO Open 11, no. 9 (2026): 108315. 10.1016/j.esmoop.2026.108315 PMC13486810 42575039

